# Synthesis and bioevaluation of new tacrine-cinnamic acid hybrids as cholinesterase inhibitors against Alzheimer’s disease

**DOI:** 10.1080/14756366.2017.1412314

**Published:** 2017-12-27

**Authors:** Yao Chen, Jie Zhu, Jun Mo, Hongyu Yang, Xueyang Jiang, Hongzhi Lin, Kai Gu, Yuqiong Pei, Liang Wu, Renxiang Tan, Jing Hou, Jingyi Chen, Yang Lv, Yaoyao Bian, Haopeng Sun

**Affiliations:** a School of Pharmacy, Nanjing University of Chinese Medicine, Nanjing, China;; b Jiangsu Collaborative Innovation Center of Chinese Medicinal Resources Industrialization, Nanjing University of Chinese Medicine, Nanjing, China;; c State Key Laboratory Cultivation Base for TCM Quality and Efficacy, Nanjing University of Chinese Medicine, Nanjing, China;; d Department of Medicinal Chemistry, China Pharmaceutical University, Nanjing, China;; e Key Laboratory of Biomedical Functional Materials, School of Science, China Pharmaceutical University, Nanjing, China;; f School of Nursing, Nanjing University of Chinese Medicine, Nanjing, China

**Keywords:** Cholinesterase inhibitor, multi-target ligand, tacrine hybrid, cinnamic acid

## Abstract

Small molecule cholinesterases inhibitor (ChEI) provides an effective therapeutic strategy to treat Alzheimer’s disease (AD). Currently, the discovery of new ChEI with multi-target effect is still of great importance. Herein, we report the synthesis, structure–activity relationship study and biological evaluation of a series of tacrine-cinnamic acid hybrids as new ChEIs. All target compounds are evaluated for their *in vitro* cholinesterase inhibitory activities. The representatives which show potent activity on cholinesterase, are evaluated for the amyloid β-protein self-aggregation inhibition and *in vivo* assays. The optimal compound **19**, **27**, and **30** (human AChE IC_50_ = 10.2 ± 1.2, 16.5 ± 1.7, and 15.3 ± 1.8 nM, respectively) show good performance in ameliorating the scopolamine-induced cognition impairment and preliminary safety in hepatotoxicity evaluation. These compounds deserve further evaluation for the development of new therapeutic agents against AD.

## Introduction

Alzheimer’s disease (AD) is one of the most prevalent forms of late-life mental failure in humans. About 6% of the population aged over 65 is affected by this disease^1^, and it is estimated that 70 million people will suffer from AD by 2050^2^. Therefore, discovery of efficient anti-AD agents is of great importance for drug developers^3^^,^^4^. So far, the mechanism for AD is still not clearly elucidated, but it is well-accepted that AD is a multifactorial syndrome deriving from a complex array of neurochemical factors^5^. Several hypotheses about AD pathogenesis are presented, such as cholinergic dysfunction^6^, amyloid cascade^7^, hyperphosphorylation of τ-protein^8^, cell cycle hypothesis^9^, and brain-derived neurotrophic factor hypothesis^10^, oxidative stress^11^, free radical formation^12^, metal dyshomeostasis^13^, and mitochondrial dysfunction^14^. These findings not only inspire the design of new anti-AD agents with diverse mechanisms, but also depict a more complex AD scenario.

So far, designing drugs targeting the cholinergic system is still the most successful therapeutic strategy against AD. Many studies have shown that the decline of acetylcholine (ACh) level results in the cognitive and memory deficits^15–17^. Therefore, recovering cholinergic function by inhibiting cholinesterases (ChEs), which are in charge of the hydrolysis of ACh, is beneficial for the treatment of AD^18^^,^^19^. There are two types of ChEs, namely, acetylcholinesterase (AChE) and butyrylcholinesterase (BuChE). Under normal condition, AChE is a dominant factor for ACh metabolism (80%), therefore, acetylcholinesterase inhibitors (AChEIs) can efficiently prevent the hydrolysis of ACh and provide promising therapeutic effects^20^. In recent decades, the function of BuChE in the development of AD is elucidated. In advanced AD, the level of AChE drops down to 90% compared to the healthy brain, leading to the loss of function of AChEIs^21^. While BuChE maintains the normal level or even up-regulated as a compensatory feedback for the metabolism of ACh. Inhibition of BuChE constitutes a promising target for clinical use, especially for progressed AD^22^. Therefore, inhibitors of both AChE and BuChE are expected to exert a potent therapeutic effect on AD. Unfortunately, current ChEs inhibitors in clinical use, such as donepezil and rivastigmine, only enable a palliative treatment^23^. Considering the multifactorial nature of AD, designing multi-target-directed ligands (MTDLs) that can simultaneously regulate multiple targets in the development of AD, has emerged as a new strategy^24–26^, and many of MTDLs have been proved to show promising pharmacological effects on AD^27–31^.

The enzymatic site of human AChE (*h*AChE) and BuChE (*h*BuChE) contains two binding sites: the catalytic active site (CAS) at the bottom and the peripheral anionic site (PAS) near the entrance of the gorge^32^^,^^33^. The two proteins share an almost 65% homologic amino acid sequences. Catalytic triads in CAS of *h*AChE and *h*BuChE consist of conserved amino acids: Ser203, His447, Glu334 in *h*AChE and Ser198, His438, Glu325 in *h*BuChE^34^. PAS of *h*AChE is proved to be closely related to both hydrolysis of ACh and neurotoxic cascade of AD through AChE-induced β-amyloid (Aβ) aggregation^35^. As a result, designing MTDLs targeting both CAS and PAS attracts the attentions of medicinal chemists throughout the world.

Herein, we report our efforts in the design, synthesis and bioevaluation of a series of tacrine-cinnamic acid hybrids as acetyl- and BuChE inhibitors against AD^36^^,^^37^. The target compounds are synthesised and evaluated for their ChEs inhibitory activities. The structure-activity relationship (SAR) of these compounds is summarised and discussed. To avoid cytotoxicity, the antiproliferative activity of the compounds are evaluated in PC-12 cells. Finally, the *in vivo* behavioural and hepatotoxic evaluations for the optimal compounds are performed. Information gathering from these experiments will guide our further optimisation of new MTDLs against AD.

## Experimental sections

### Chemistry

#### General experimental

^1^H-NMR and ^13 ^C-NMR spectra were determined by a Bruker Avance 300 MHz spectrometer at 300 K, with TMS as an internal standard. MS spectra were determined on a Mariner Mass spectrum (ESI) or a LC/MSD TOF HR-MS spectrum. Melting points were obtained using a Mel-TEMP II melting point apparatus. Thin-layer chromatography was carried out on silica gel GF/UV 254 supported by glass plate, and the chromatograms were performed on silica gel (200–300 mesh) visualised under UV light at 254 and 365 nm. Purity for final compounds was measured by HPLC with Agilent Technologies 1260 infinity C_18_ 4.60 mm ×150 mm column using a mixture of solvent methanol/water at the flow rate of 0.5 ml/min and peak detection at 254 nm under UV. All solvents were reagent grade without special instruction. When necessary, solvents were purified and dried. The vacuum distillation was performed by using a rotary evaporator at a reduced pressure of ca. 20 Torr. Organic solutions were dried over anhydrous sodium sulphate.

*N-(2-((1,2,3,4-tetrahydroanthracen-9-yl)amino)ethyl)cinnamamide (**9**)*. Yellow powder, yield: 39%, mp 55–57 °C. 86–88 °C^44^. ^1^H NMR (300 MHz, CDCl_3_): *δ* 7.96 (d, *J* = 8.5 Hz, 1H), 7.88 (d, *J* = 8.4 Hz, 1H), 7.66 (d, *J* = 15.6 Hz, 1H), 7.55–7.44 (m, 3H), 7.37–7.34 (m, 3H), 7.30 (d, *J* = 7.1 Hz, 1H), 6.44 (s, 1H), 6.39 (s, 1H), 3.70 (s, 4H), 3.01 (s, 2H), 2.70 (s, 2H), 1.86 (s, 4H). ^13 ^C NMR (500 MHz, CDCl_3_): *δ* 167.61, 157.94, 151.06, 146.58, 141.52, 134.68, 129.85, 128.87, 128.76, 127.85, 127.67, 123.86, 122.85, 120.37, 119.73, 115.85, 49.85, 40.68, 33.43, 25.01, 22.95, 22.54. HRMS (ESI) *m/z* calcd for C_24_H_25_N_3_O [M + H]^+^ 372.2065; found 372.2065. HPLC (70% methanol in water with 0.5% H_3_PO_4_): *t*_R_ = 3.95 min, 97.85%.

*N-(3-((1,2,3,4-tetrahydroanthracen-9-yl)amino)propyl)cinnamamide (**10**)*. Yellow powder, yield: 43%, mp 58–60 °C. 130–132 °C^44^. ^1^H NMR (300 MHz, CDCl_3_): *δ* 8.12 (d, *J* = 8.5 Hz, 1H), 8.01 (d, *J* = 8.4 Hz, 1H), 7.67 (d, *J* = 15.7 Hz, 1H), 7.60–7.45 (m, 3H), 7.43–7.30 (m, 4H), 6.99 (s, 1H), 6.57 (d, *J* = 15.7 Hz, 1H), 5.75 (s, 1H), 3.66 (s, 2H), 3.59–3.46 (m, 2H), 3.08 (s, 2H), 2.77 (s, 2H), 1.89 (s, 6H). ^13 ^C NMR (500 MHz, CDCl_3_): *δ* 167.27, 156.36, 152.20, 144.83, 141.03, 134.83, 129.69, 129.45, 128.80, 127.83, 126.03, 124.22, 123.19, 120.79, 119.13, 115.02, 44.93, 36.62, 32.37, 31.37, 24.95, 22.81, 22.18. HRMS (ESI) *m/z* calcd for C_25_H_27_N_3_O [M + H]^+^ 386.2223; found 386.2223. HPLC (70% methanol in water with 0.5% H_3_PO_4_): *t*_R_ = 4.04 min, 98.27%.

*N-(4-((1,2,3,4-tetrahydroacridin-9-yl)amino)butyl)cinnamamide (**11**)*. Yellow powder, yield: 36%, mp 73–75 °C. 155–157 °C^29^. ^1^H NMR (300 MHz, CDCl_3_): *δ* 7.91 (t, *J* = 9.1 Hz, 2H), 7.62 (d, *J* = 15.6 Hz, 1H), 7.54 (t, *J* = 7.6 Hz, 1H), 7.47 (d, *J* = 3.6 Hz, 1H), 7.45 (d, *J* = 2.1 Hz, 1H), 7.35–7.31 (m, 4H), 6.36 (d, *J* = 15.6 Hz, 1H), 6.02 (s, 1H), 3.97 (s, 1H), 3.49 (d, *J* = 5.4 Hz, 2H), 3.44–3.38 (m, 2H), 3.04 (s, 2H), 2.69 (s, 2H), 2.03 (s, 2H), 1.93–1.85 (m, 4H), 1.68 (s, 2H). ^13 ^C NMR (500 MHz, CDCl_3_): *δ* 166.20, 158.59, 150.56, 147.44, 140.90, 134.86, 129.65, 128.81, 128.65, 128.37, 127.76, 123.80, 122.75, 120.80, 120.37, 116.33, 48.94, 39.36, 34.01, 29.07, 27.24, 24.88, 23.04, 22.76. HRMS (ESI) *m/z* calcd for C_26_H_29_N_3_O [M + H]^+^ 400.2380; found 400.2380. HPLC (70% methanol in water with 0.5% H_3_PO_4_): *t*_R_ = 3.92 min, 99.02%.

*N-(6-((1,2,3,4-tetrahydroacridin-9-yl)amino)hexyl)cinnamamide (**12**)*. Yellow powder, yield: 52%, mp 53–55 °C. ^1^H NMR (300 MHz, CDCl_3_): *δ* 7.99 (d, *J* = 9.3 Hz, 1H), 7.94 (d, *J* = 8.1 Hz, 1H), 7.60–7.53 (m, 2H), 7.49–7.41 (m, 2H), 7.36 (t, *J* = 7.1 Hz, 1H), 7.04 (t, *J* = 8.6 Hz, 2H), 6.35 (d, *J* = 15.6 Hz, 1H), 6.07 (s, 1H), 3.53 (t, *J* = 7.2 Hz, 2H), 3.40–3.34 (m, 2H), 3.07 (s, 2H), 2.70 (s, 2H), 1.96–1.84 (m, 4H), 1.74–1.63 (m, 2H), 1.60–1.49 (m, 2H), 1.47–1.37 (m, 4H). ^13 ^C NMR (500 MHz, CDCl_3_): *δ* 166.06, 158.30, 150.93, 147.22, 140.78, 134.93, 129.60, 128.80, 128.49,128.42, 127.76, 123.71, 122.91, 120.90, 120.16, 115.83, 49.30, 39.55, 33.85, 31.63, 29.63, 26.65, 26.57, 24.82, 23.04, 22.74. HRMS (ESI) *m/z* calcd for C_28_H_33_N_3_O [M + H]^+^ 428.2694; found 428.2694. HPLC (70% methanol in water with 0.5% H_3_PO_4_): *t*_R_ = 4.97 min, 96.68%.

*N-(8-((1,2,3,4-tetrahydroacridin-9-yl)amino)hexyl)cinnamamide (**13**)*. Yellow powder, yield: 30%, mp 50–52 °C. ^1^H NMR (300 MHz, CDCl_3_): *δ* 7.98 (d, *J* = 6.0 Hz, 1H), 7.95 (d, *J* = 5.7 Hz, 1H), 7.60 (d, *J* = 15.7 Hz, 1H), 7.57–7.50 (m, 1H), 7.47–7.44 (m, 2H), 7.40–7.25 (m, 5H), 6.46 (d, *J* = 15.6 Hz, 1H), 6.23 (s, 1H), 3.51 (t, *J* = 7.0 Hz, 2H), 3.38–3.31(m, 2H), 3.07 (s, 2H), 2.68 (s, 2H), 1.89 (s, 4H), 1.71–1.59 (m, 2H), 1.56–1.47 (m, 2H), 1.29 (s, 8H). ^13 ^C NMR (500 MHz, CDCl_3_): *δ* 166.07, 157.62, 151.46, 146.48, 140.55, 135.01, 129.51, 128.80, 128.77, 127.74, 127.71, 123.79, 123.10, 121.13, 119.73, 115.25, 49.31, 39.72, 33.36, 31.65, 31.28, 29.61, 29.10, 26.79, 26.74, 24.69, 22.94, 22.56. HRMS (ESI) *m/z* calcd for C_30_H_37_N_3_O [M + H]^+^ 456.3006; found 456.3006. HPLC (70% methanol in water with 0.5% H_3_PO_4_): *t*_R_ = 7.73 min, 98.24%.

*(E)-N-(6-((1,2,3,4-tetrahydroacridin-9-yl)amino)hexyl)-3-(o-tolyl)acrylamide (**14**)*. Yellow powder, yield: 33%, mp 55–57 °C. ^1^H NMR (300 MHz, CDCl_3_): *δ* 8.00 (t, *J* = 7.6 Hz, 2H), 7.92 (d, *J* = 15.5 Hz, 1H), 7.57 (t, *J* = 7.5 Hz, 1H), 7.50 (d, *J* = 7.6 Hz, 1H), 7.37 (t, *J* = 7.6 Hz, 1H), 7.24 (d, *J* = 7.0 Hz, 1H), 7.19 (d, *J* = 7.6 Hz, 2H), 6.33 (d, *J* = 15.4 Hz, 1H), 5.94 (s, 1H), 3.57 (t, *J* = 7.1 Hz, 2H), 3.42–3.36 (m, 2H), 3.10 (s, 2H), 2.70 (s, 2H), 2.43 (s, 3H), 1.92 (s, 4H), 1.75–1.66 (m, 2H), 1.63–1.56 (m, 2H), 1.45 (s, 4H). ^13 ^C NMR (500 MHz, CDCl_3_): *δ* 166.20, 157.33, 151.58, 146.06, 138.52, 137.43, 133.97, 130.70, 129.32, 128.96, 127.32, 126.15, 126.09, 123.88, 123.11, 122.10, 119.57, 115.14, 49.11, 39.51, 33.05, 31.50, 29.58, 26.59, 26.49, 24.68, 22.88, 22.45, 19.82. HRMS (ESI) *m/z* calcd for C_29_H_35_N_3_O [M + H]^+^ 442.286; found 442.2862. HPLC (70% methanol in water with 0.5% H_3_PO_4_): *t*_R_ = 6.62 min, 95.09%.

*(E)-N-(6-((1,2,3,4-tetrahydroacridin-9-yl)amino)hexyl)-3-(m-tolyl)acrylamide (**15**)*. Yellow powder, yield: 27%, mp 53–55 °C. ^1^H NMR (300 MHz, CDCl_3_): *δ* 7.95 (t, *J* = 9.1 Hz, 2H), 7.59 (d, *J* = 11.7 Hz, 1H), 7.54 (d, *J* = 10.1 Hz, 1H), 7.36 (d, *J* = 7.8 Hz, 1H), 7.30 (d, *J* = 8.6 Hz, 2H), 7.23 (d, *J* = 8.1 Hz, 1H), 7.15 (d, *J* = 7.0 Hz, 1H), 6.37 (d, *J* = 15.6 Hz, 1H), 5.77 (s, 1H), 3.50 (t, *J* = 7.1 Hz, 2H), 3.34–3.33 (m, 2H), 3.07 (s, 2H), 2.70 (s, 2H), 2.35 (s, 3H), 1.91 (s, 4H), 1.73–1.62 (m, 2H), 1.60–1.52 (m, 2H), 1.40 (s, 4H). ^13 ^C NMR (500 MHz, CDCl_3_): *δ* 166.18, 157.95, 151.15, 140.87, 138.41, 134.88, 130.41, 128.67, 128.62, 128.49, 128.02, 124.88, 123.75, 122.98, 120.74, 119.94, 115.55, 49.22, 39.52, 33.54, 31.59, 29.62, 26.62, 26.54, 24.77, 22.98, 22.63, 21.33. HRMS (ESI) *m/z* calcd for C_29_H_35_N_3_O [M + H]^+^ 442.285; found 442.2858. HPLC (70% methanol in water with 0.5% H_3_PO_4_): *t*_R_ = 7.35 min, 95.17%.

*(E)-N-(6-((1,2,3,4-tetrahydroacridin-9-yl)amino)hexyl)-3-(p-tolyl)acrylamide (**16**)*. Yellow powder, yield: 33%, mp 52–53 °C. ^1^H NMR (300 MHz, CDCl_3_): *δ* 7.96–7.89 (m, 2H), 7.63–7.50 (m, 2H), 7.41–7.30 (m, 3H), 7.16 (d, *J* = 8.0 Hz, 2H), 6.32 (d, *J* = 15.6 Hz, 1H), 5.77 (s, 1H), 3.47 (t, *J* = 7.2 Hz, 2H), 3.39–3.33 (m, 2H), 3.06 (s, 2H), 2.71 (s, 2H), 2.35 (s, 3H), 1.91 (t, *J* = 3.2 Hz, 4H), 1.71–1.61 (m, 2H), 1.60–1.50 (m, 2H), 1.46–1.34 (m, 4H). ^13 ^C NMR (500 MHz, CDCl_3_): *δ* 166.26, 158.50, 150.79, 147.47, 140.73, 139.85, 132.16, 129.52, 128.66, 128.33, 127.73, 123.65, 122.87, 120.28, 119.85, 115.97, 49.34, 39.53, 34.01, 31.66, 29.66, 26.68, 26.60, 24.85, 23.08, 22.80, 21.40. HRMS (ESI) *m/z* calcd for C_29_H_35_N_3_O [M + H]^+^ 442.2853; found 442.2860. HPLC (70% methanol in water with 0.5% H_3_PO_4_): *t*_R_ = 6.42 min, 95.67%.

*(E)-3-(2-nitrophenyl)-N-(6-((1,2,3,4-tetrahydroacridin-9-yl)amino)hexyl)acrylamide (**17**)*. Yellow powder, yield: 23%, mp 62–63 °C. ^1^H NMR (300 MHz, CDCl_3_): *δ* 8.05–7.97 (m, 2H), 7.93 (t, *J* = 9.4 Hz, 2H), 7.66–7.56 (m, 2H), 7.55–7.48 (m, 2H), 7.35 (t, *J* = 7.4 Hz, 1H), 6.33 (d, *J* = 15.6 Hz, 1H), 6.03 (s, 1H), 3.50 (t, *J* = 7.1 Hz, 2H), 3.41–3.34 (m, 2H), 3.06 (s, 2H), 2.72 (s, 2H), 1.93 (s, 4H), 1.75–1.64 (m, 2H), 1.62–1.52 (m, 2H), 1.42 (s, 4H). ^13 ^C NMR (500 MHz, CDCl_3_): *δ* 165.02, 158.37, 150.87, 148.32, 147.28, 135.74, 133.33, 131.12, 129.71, 129.10, 128.48, 128.40, 126.33, 124.81, 123.68, 122.89, 120.18, 115.90, 49.29, 39.65, 33.91, 31.61, 29.58, 26.62, 26.54, 24.82, 23.05, 22.76. HRMS (ESI) *m/z* calcd for C_28_H_32_N_4_O_3_ [M + H]^+^ 473.2547; found 473.2546. HPLC (70% methanol in water with 0.5% H_3_PO_4_): *t*_R_ = 4.27 min, 95.56%.

*(E)-3-(3-nitrophenyl)-N-(6-((1,2,3,4-tetrahydroacridin-9-yl)amino)hexyl)acrylamide (**18**)*. Yellow powder, yield: 30%, mp 62–63 °C. ^1^H NMR (300 MHz, CDCl_3_): *δ* 8.34 (s, 1H), 8.19–8.16 (m, 1H), 7.96–7.88 (m, 2H), 7.73 (d, *J* = 7.7 Hz, 1H), 7.65 (d, *J* = 15.6 Hz, 1H), 7.57–7.50 (m, 2H), 7.38–7.30 (m, 1H), 6.52 (d, *J* = 15.6 Hz, 1H), 6.02 (s, 1H), 3.48 (t, *J* = 7.1 Hz, 2H), 3.42–3.36 (m, 2H), 3.05 (s, 2H), 2.70 (s, 2H), 1.91 (t, *J* = 3.0 Hz, 4H), 1.72–1.62 (m, 2H), 1.62–1.54 (m, 2H), 1.42–1.37 (m, 4H). ^13 ^C NMR (500 MHz, CDCl_3_): *δ* 165.17, 157.95, 151.13, 148.57, 146.81, 137.88, 136.80, 133.88, 129.83, 128.61, 127.94, 124.17, 123.76, 123.73, 123.00, 121.60, 119.92, 115.56, 49.20, 39.64, 33.56, 31.56, 29.48, 26.61, 26.51, 24.75, 22.97, 22.63. HRMS (ESI) *m/z* calcd for C_28_H_32_N_4_O_3_ [M + H]^+^ 473.2547; found 473.2554. HPLC (70% methanol in water with 0.5% H_3_PO_4_): *t*_R_ = 5.08 min, 95.08%.

*(E)-3-(4-nitrophenyl)-N-(6-((1,2,3,4-tetrahydroacridin-9-yl)amino)hexyl)acrylamide (**19**)*. Yellow powder, yield: 33%, mp 62–63 °C. ^1^H NMR (300 MHz, CDCl_3_): *δ* 8.22 (s, 1H), 8.19 (s, 1H), 7.97 (d, *J* = 8.3 Hz, 1H), 7.91 (d, *J* = 8.4 Hz, 1H), 7.66 (d, *J* = 15.6 Hz, 1H), 7.61 (s, 1H), 7.58 (s, 1H), 7.54 (d, *J* = 7.3 Hz, 1H), 7.35 (t, *J* = 7.6 Hz, 1H), 6.51 (d, *J* = 15.6 Hz, 1H), 6.11 (s, 1H), 3.50 (t, *J* = 7.1 Hz, 2H), 3.42–3.36 (m, 2H), 3.06 (s, 2H), 2.71 (s, 2H), 1.93 (s, 4H), 1.72–1.63 (m, 2H), 1.63–1.55 (m, 2H), 1.41 (s, 4H). ^13 ^C NMR (500 MHz, CDCl_3_): *δ* 164.86, 158.50, 150.79, 148.10, 147.42, 141.21, 138.22, 128.61, 128.40, 128.30, 125.02, 124.11, 123.69, 122.84, 120.24, 115.96, 49.29, 39.70, 33.98, 31.63, 29.54, 26.61, 26.54, 24.85, 23.07, 22.79. HRMS (ESI) *m/z* calcd for C_28_H_32_N_4_O_3_ [M + H]^+^ 473.2547; found 473.2549. HPLC (70% methanol in water with 0.5% H_3_PO_4_): *t*_R_ = 4.62 min, 95.83%.

*(E)-3-(4-chloro-3-nitrophenyl)-N-(6-((1,2,3,4-tetrahydroacridin-9-yl)amino)hexyl)acrylamide (**20**)*. Yellow powder, yield: 46%, mp 55–60 °C. ^1^H NMR (300 MHz, CDCl_3_): *δ* 7.96 (s, 1H), 7.93 (d, *J* = 4.4 Hz, 1H), 7.89 (s, 1H), 7.58 (d, *J* = 5.6 Hz, 1H), 7.54 (s, 2H), 7.47 (d, *J* = 10.8 Hz, 1H), 7.34 (t, *J* = 7.8 Hz, 1H), 6.44 (d, *J* = 15.6 Hz, 1H), 5.93 (s, 1H), 3.49 (t, *J* = 7.0 Hz, 2H), 3.41–3.34 (m, 2H), 3.06 (s, 2H), 2.70 (s, 2H), 1.92 (s, 4H), 1.72–1.62 (m, 2H), 1.59–1.49 (m, 2H), 1.40 (d, *J* = 3.2 Hz, 4H). ^13 ^C NMR (500 MHz, CDCl_3_): *δ* 164.83, 158.19, 150.97, 148.15, 147.07, 136.79, 135.21, 132.31, 132.11, 131.46, 128.52, 128.22, 127.36, 124.51, 123.70, 122.96, 120.05, 115.71, 49.23, 39.66, 33.76, 31.59, 29.48, 26.61, 26.51, 24.79, 23.01, 22.70. HRMS (ESI) *m/z* calcd for C_28_H_31_ClN_4_O_3_ [M + H]^+^ 507.216; found 507.2165. HPLC (70% methanol in water with 0.5% H_3_PO_4_): *t*_R_ = 6.78 min, 95.01%.

*(E)-N-(6-((1,2,3,4-tetrahydroacridin-9-yl)amino)hexyl)-3–(2-(trifluoromethyl)phenyl)acrylamide (**21**)*. Yellow powder, yield: 43%, mp 57–60 °C. ^1^H NMR (300 MHz, CDCl_3_): *δ* 8.00–7.91 (m, 3H), 7.69–7.61 (m, 2H), 7.59–7.47 (m, 2H), 7.42 (t, *J* = 7.4 Hz, 1H), 7.35 (t, *J* = 7.5 Hz, 1H), 6.39 (d, *J* = 15.4 Hz, 1H), 6.02 (s, 1H), 3.53 (t, *J* = 7.0 Hz, 2H), 3.40–3.34 (m, 2H), 3.07 (s, 2H), 2.70 (s, 2H), 1.90 (s, 4H), 1.70–1.64 (m, 2H), 1.61–1.51 (m, 2H), 1.41 (s, 4H). ^13 ^C NMR (500 MHz, CDCl_3_): *δ* 165.35, 156.95, 151.78, 145.68, 136.09, 134.12, 131.95, 129.11, 128.89, 128.47, 127.82, 126.94, 126.06, 126.02, 125.64, 123.93, 123.21, 119.36, 114.94, 48.96, 39.51, 32.86, 31.40, 29.46, 26.49, 26.37, 24.64, 22.82, 22.35. HRMS (ESI) *m/z* calcd for C_29_H_32_F_3_N_3_O [M + H]^+^ 496.258; found 496.2580. HPLC (70% methanol in water with 0.5% H_3_PO_4_): *t*_R_ = 7.58 min, 95.34%.

*(E)-N-(6-((1,2,3,4-tetrahydroacridin-9-yl)amino)hexyl)-3–(3-(trifluoromethyl)phenyl)acrylamide (**22**)*. Yellow powder, yield: 38%, mp 48–54 °C. ^1^H NMR (300 MHz, DMSO): *δ* 8.13 (s, 1H), 8.10 (d, *J* = 5.1 Hz, 1H), 7.91–7.83 (m, 1H), 7.78–7.68 (m, 2H), 7.68–7.59 (m, 1H), 7.53–7.46 (m, 2H), 7.34 (t, *J* = 7.4 Hz, 1H), 6.75 (d, *J* = 15.7 Hz, 1H), 5.45 (s, 1H), 3.41 (s, 2H), 3.15 (d, *J* = 5.9 Hz, 2H), 2.90 (s, 2H), 2.71 (s, 2H), 1.80 (s, 4H), 1.56 (s, 2H), 1.42 (s, 2H), 1.30 (s, 4H). ^13 ^C NMR (500 MHz, CDCl_3_): *δ* 165.43, 157.26, 151.60, 145.90, 139.03, 135.77, 131.14, 131.07, 129.36, 128.99, 127.21, 125.96, 123.98, 123.95, 123.88, 123.07, 122.86, 119.49, 115.01, 49.04, 39.55, 32.90, 31.50, 29.54, 26.53, 26.43, 24.67, 22.86, 22.40. HRMS (ESI) *m/z* calcd for C_29_H_32_F_3_N_3_O [M + H]^+^ 496.258; found 496.2580. HPLC (70% methanol in water with 0.5% H_3_PO_4_): *t*_R_ = 11.99 min, 96.20%.

*(E)-N-(6-((1,2,3,4-tetrahydroacridin-9-yl)amino)hexyl)-3–(4-(trifluoromethyl)phenyl)acrylamide (**23**)*. Yellow powder, yield: 44%, mp 58–62 °C. ^1^H NMR (300 MHz, CDCl_3_): *δ* 7.97 (d, *J* = 8.7 Hz, 1H), 7.93 (d, *J* = 8.6 Hz, 1H), 7.63 (d, *J* = 19.4 Hz, 3H), 7.58–7.53 (m, 3H), 7.36 (t, *J* = 7.6 Hz, 1H), 6.47 (d, *J* = 15.6 Hz, 1H), 6.01 (s, 1H), 3.51 (t, *J* = 7.0 Hz, 2H), 3.42–3.36 (m, 2H), 3.08 (s, 2H), 2.72 (s, 2H), 1.93 (s, 4H), 1.68 (t, *J* = 6.8 Hz, 2H), 1.64–1.57 (m, 2H), 1.43 (s, 4H). ^13 ^C NMR (500 MHz, CDCl_3_): *δ* 165.61, 158.16, 151.04, 147.06, 138.66, 138.48, 128.53, 128.07, 127.80, 125.65, 125.62, 123.77, 123.70, 123.01, 120.05, 115.65, 49.20, 39.60, 33.68, 31.56, 29.50, 26.65, 26.52, 24.75, 22.96, 22.64. HRMS (ESI) *m/z* calcd for C_29_H_32_F_3_N_3_O [M + H]^+^ 496.2571; found 496.2571. HPLC (70% methanol in water with 0.5% H_3_PO_4_): *t*_R_ = 8.34 min, 95.08%.

*(E)-3-(4-fluorophenyl)-N-(6-((1,2,3,4-tetrahydroacridin-9-yl)amino)hexyl)acrylamide (**24**)*. Yellow powder, yield: 48%, mp 55–56 °C. ^1^H NMR (300 MHz, CDCl_3_): *δ* 7.97 (d, *J* = 8.4 Hz, 1H), 7.93 (d, *J* = 8.5 Hz, 1H), 7.63 (d, *J* = 15.6 Hz, 1H), 7.56 (t, *J* = 8.1 Hz, 1H), 7.50 (d, *J* = 3.9 Hz, 1H), 7.47 (d, *J* = 1.9 Hz, 1H), 7.40–7.32 (m, 4H), 6.41 (d, *J* = 15.6 Hz, 1H), 5.93 (s, 1H), 3.50 (t, *J* = 7.1 Hz, 2H), 3.41–3.35 (m, 2H), 3.07 (s, 2H), 2.71 (s, 2H), 1.92 (s, 4H), 1.73–1.63 (m, 2H), 1.62–1.51 (m, 2H), 1.42 (s, 4H). ^13 ^C NMR (500 MHz, CDCl_3_): *δ* 165.91, 164.50, 162.51, 151.32, 146.54, 139.61, 131.13, 129.57, 129.50, 128.80, 127.77, 123.84, 123.01, 120.60, 119.81, 115.98, 115.81, 115.44, 49.18, 39.53, 33.37, 31.55, 29.60, 26.57, 26.50, 24.73, 22.94, 22.56. HRMS (ESI) *m/z* calcd for C_28_H_32_FN_3_O_3_ [M + H]^+^ 446.2602; found 446.2601. HPLC (70% methanol in water with 0.5% H_3_PO_4_): *t*_R_ = 5.50 min, 94.98%.

*(E)-3-(2-chlorophenyl)-N-(6-((1,2,3,4-tetrahydroacridin-9-yl)amino)hexyl)acrylamide (**25**)*. Yellow powder, yield: 51%, mp 53–55 °C. ^1^H NMR (300 MHz, CDCl_3_): *δ* 7.98 (t, *J* = 15.4 Hz, 3H), 7.56 (t, *J* = 7.1 Hz, 2H), 7.41–7.33 (m, 2H), 7.28–7.20 (m, 2H), 6.44 (d, *J* = 15.6 Hz, 1H), 6.08 (s, 1H), 3.53 (t, *J* = 6.7 Hz, 2H), 3.39 (d, *J* = 6.2 Hz, 2H), 3.08 (s, 2H), 2.71 (s, 2H), 1.92 (s, 4H), 1.69 (s, 2H), 1.59 (s, 2H), 1.43 (s, 4H). ^13 ^C NMR (500 MHz, CDCl_3_): *δ* 165.61, 158.24, 150.96, 147.14, 136.63, 134.68, 133.24, 130.36, 130.13, 128.48, 128.32, 127.53, 126.93, 123.82, 123.70, 122.92, 120.10, 115.78, 49.28, 39.60, 33.79, 31.61, 29.57, 26.63, 26.55, 24.80, 23.03, 22.72. HRMS (ESI) *m/z* calcd for C_28_H_32_ClN_3_O [M + H]^+^ 462.231; found 462.2314. HPLC (70% methanol in water with 0.5% H_3_PO_4_): *t*_R_ = 6.43 min, 96.83%.

*(E)-3-(3-chlorophenyl)-N-(6-((1,2,3,4-tetrahydroacridin-9-yl)amino)hexyl)acrylamide (**26**)*. Yellow powder, yield: 32%, mp 55–59 °C. ^1^H NMR (300 MHz, CDCl_3_): *δ* 8.00 (d, *J* = 4.0 Hz, 1H), 7.97 (d, *J* = 4.0 Hz, 1H), 7.58 (d, *J* = 5.4 Hz, 1H), 7.51 (d, *J* = 15.9 Hz, 2H), 7.38 (d, *J* = 7.5 Hz, 1H), 7.33 (d, *J* = 7.4 Hz, 3H), 6.41 (d, *J* = 15.6 Hz, 1H), 5.89 (s, 1H), 3.55 (t, *J* = 7.0 Hz, 2H), 3.37 (d, *J* = 6.6 Hz, 2H), 3.10 (s, 2H), 2.71 (s, 2H), 1.93 (s, 4H), 1.75–1.65 (m, 2H), 1.63–1.53 (m, 2H), 1.44 (s, 4H). ^13 ^C NMR (500 MHz, CDCl_3_): *δ* 165.64, 157.32, 151.53, 146.03, 139.08, 136.84, 134.73, 130.03, 129.39, 128.94, 127.37, 127.30, 126.11, 123.86, 123.11, 122.49, 119.55, 115.09, 49.06, 39.54, 33.04, 31.50, 29.53, 26.56, 26.46, 24.69, 22.88, 22.44. HRMS (ESI) *m/z* calcd for C_28_H_32_ClN_3_O [M + H]^+^ 462.231; found 462.2316. HPLC (70% methanol in water with 0.5% H_3_PO_4_): *t*_R_ = 7.31 min, 95.25%.

*(E)-3-(4-chlorophenyl)-N-(6-((1,2,3,4-tetrahydroacridin-9-yl)amino)hexyl)acrylamide (**27**)*. Yellow powder, yield: 39%, mp 56–57 °C. ^1^H NMR (300 MHz, CDCl_3_): *δ* 7.99 (d, *J* = 8.5 Hz, 1H), 7.94 (d, *J* = 9.3 Hz, 1H), 7.60–7.52 (m, 2H), 7.42–7.34 (m, 3H), 7.34–7.29 (m, 2H), 6.41 (d, *J* = 15.6 Hz, 1H), 6.19 (s, 1H), 3.53 (t, *J* = 7.2 Hz, 2H), 3.40–3.33 (m, 2H), 3.07 (s, 2H), 2.69 (s, 2H), 1.91 (s, 4H), 1.72–1.62 (m, 2H), 1.62–1.52 (m, 2H), 1.42–1.38 (m, 4H). ^13 ^C NMR (500 MHz, CDCl_3_): *δ* 165.69, 157.61, 151.37, 139.51, 139.45, 135.44, 133.43, 130.46, 129.06, 128.92, 128.84, 128.54, 127.76, 123.85, 123.01, 121.38, 119.72, 115.34, 49.15, 39.53, 33.27, 31.53, 29.58, 26.55, 26.48, 24.71, 22.93, 22.53. HRMS (ESI) *m/z* calcd for C_28_H_32_ClN_3_O [M + H]^+^ 462.2307; found 462.2306. HPLC (70% methanol in water with 0.5% H_3_PO_4_): *t*_R_ = 8.09 min, 95.22%.

*(E)-3-(4-bromophenyl)-N-(6-((1,2,3,4-tetrahydroacridin-9-yl)amino)hexyl)acrylamide (**28**)*. Yellow powder, yield: 59%, mp 60–61 °C. ^1^H NMR (300 MHz, CDCl_3_): *δ* 7.97 (d, *J* = 8.5 Hz, 1H), 7.93 (d, *J* = 8.5 Hz, 1H), 7.61–7.53 (m, 2H), 7.50 (d, *J* = 8.4 Hz, 2H), 7.36 (t, *J* = 7.3 Hz, 3H), 6.37 (d, *J* = 15.6 Hz, 1H), 5.76 (s, 1H), 3.51 (t, *J* = 7.1 Hz, 2H), 3.41–3.35 (m, 2H), 3.08 (s, 2H), 2.72 (s, 2H), 1.93 (s, 4H), 1.74–1.63 (m, 2H), 1.62–1.50 (m, 2H), 1.42 (s, 4H). ^13 ^C NMR (500 MHz, CDCl_3_): *δ* 165.66, 158.36, 150.86, 147.27, 139.57, 133.83, 132.02, 129.16, 128.50, 128.43, 123.72, 123.70, 122.87, 121.47, 120.17, 115.88, 49.30, 39.59, 33.89, 31.63, 29.60, 26.63, 26.56, 24.83, 23.05, 22.76. HRMS (ESI) *m/z* calcd for C_28_H_32_BrN_3_O [M + H]^+^ 506.1802; found 506.1807. HPLC (70% methanol in water with 0.5% H_3_PO_4_): *t*_R_ = 8.28 min, 97.37%.

*(E)-3-(4-cyanophenyl)-N-(6-((1,2,3,4-tetrahydroacridin-9-yl)amino)hexyl)acrylamide (**29**)*. Yellow powder, yield: 23%, mp 55–57 °C. ^1^H NMR (300 MHz, CDCl_3_): *δ* 7.97 (d, *J* = 8.3 Hz, 1H), 7.92 (d, *J* = 8.4 Hz, 1H), 7.71 (s, 1H), 7.65 (d, *J* = 7.3 Hz, 1H), 7.58 (d, *J* = 5.1 Hz, 2H), 7.53 (s, 2H), 7.49–7.39 (m, 1H), 7.39–7.29 (m, 1H), 6.49 (d, *J* = 15.6 Hz, 1H), 6.36 (s, 1H), 3.51 (d, *J* = 6.2 Hz, 2H), 3.37 (d, *J* = 5.8 Hz, 2H), 3.05 (s, 2H), 2.68 (s, 2H), 1.89 (s, 4H), 1.67 (s, 2H), 1.57 (s, 2H), 1.40 (s, 4H). ^13 ^C NMR (500 MHz, CDCl_3_): *δ* 165.22, 157.51, 151.44, 146.28, 138.01, 136.32, 132.45, 132.05, 130.67, 129.71, 128.89, 127.46, 123.85, 123.71, 123.12, 119.65, 118.36, 115.26, 113.07, 49.11, 39.60, 33.22, 31.50, 29.47, 26.56, 26.46, 24.70, 22.90, 22.50. HRMS (ESI) *m/z* calcd for C_29_H_32_N_4_O [M + H]^+^ 453.2648; found 453.2648. HPLC (70% methanol in water with 0.5% H_3_PO_4_): *t*_R_ = 3.57 min, 96.58%.

*(E)-3-(4-methoxyphenyl)-N-(6-((1,2,3,4-tetrahydroacridin-9-yl)amino)hexyl)acrylamide (**30**)*. Yellow powder, yield: 26%, mp 53–54 °C. ^1^H NMR (300 MHz, CDCl_3_): *δ* 7.96–7.88(m, 2H), 7.59–7.51 (m, 2H), 7.42 (d, *J* = 8.7 Hz, 2H), 7.38–7.29 (m, 1H), 6.87 (d, *J* = 8.7 Hz, 2H), 6.24 (d, *J* = 15.5 Hz, 1H), 5.79 (s, 1H), 3.82 (s, 3H), 3.47 (t, J = 7.1 Hz, 2H), 3.39–3.32 (m, 2H), 3.05 (s, 2H), 2.70 (s, 2H), 1.91 (t, *J* = 3.0 Hz, 4H), 1.70–1.61 (m, 2H), 1.58–1.49 (m, 2H), 1.44–1.34 (m, 4H). ^13 ^C NMR (500 MHz, CDCl_3_): *δ* 166.38, 160.81, 158.53, 150.74, 147.51, 140.35, 129.27, 128.70, 128.29, 127.62, 123.63, 122.86, 120.30, 118.52, 115.99, 114.22, 55.35, 49.34, 39.51, 34.07, 31.65, 29.67, 26.68, 26.60, 24.85, 23.08, 22.81. HRMS (ESI) *m/z* calcd for C_29_H_35_N_3_O_2_ [M + H]^+^ 458.2802; found 462.2805. HPLC (70% methanol in water with 0.5% H_3_PO_4_): *t*_R_ = 4.42 min, 95.51%.

*(E)-N-(6-((1,2,3,4-tetrahydroacridin-9-yl)amino)hexyl)-3–(2,3,4-trimethoxyphenyl)acrylamide (**31**)*. Yellow powder, yield: 43%, mp 56–60 °C. ^1^H NMR (300 MHz, CDCl_3_): *δ* 8.21 (d, *J* = 8.5 Hz, 1H), 8.13 (d, *J* = 8.2 Hz, 1H), 7.74 (d, *J* = 15.8 Hz, 1H), 7.58 (t, *J* = 7.7 Hz, 1H), 7.37 (t, *J* = 7.7 Hz, 1H), 7.20 (t, *J* = 6.1 Hz, 1H), 6.54 (d, *J* = 15.8 Hz, 1H), 6.32 (s, 1H), 3.89–3.85 (m, 9H), 3.74 (t, *J* = 7.0 Hz, 2H), 3.40–3.34 (m, 2H), 3.17 (t, *J* = 5.4 Hz, 2H), 2.65 (t, *J* = 5.7 Hz, 2H), 1.86 (d, *J* = 4.3 Hz, 4H), 1.79–1.67 (m, 2H), 1.64–1.52 (m, 2H), 1.43 (s, 4H). ^13 ^C NMR (500 MHz, CDCl_3_): *δ* 166.71, 156.74, 154.85, 153.02, 151.92, 145.31, 142.41, 135.51, 129.16, 126.66, 123.92, 123.19, 123.10, 122.01, 120.37, 119.21, 114.66, 107.59, 61.29, 60.88, 56.04, 48.93, 39.40, 32.52, 31.43, 29.60, 26.52, 26.42, 24.64, 22.80, 22.27. HRMS (ESI) *m/z* calcd for C_31_H_39_N_3_O_4_ [M + H]^+^ 518.301; found 518.3019. HPLC (70% methanol in water with 0.5% H_3_PO_4_): *t*_R_ = 4.44 min, 95.59%.

*(E)-N-(6-((1,2,3,4-tetrahydroacridin-9-yl)amino)hexyl)-3–(3,4,5-trimethoxyphenyl)acrylamide (**32**)*. Yellow powder, yield: 29%, mp 58–60 °C. ^1^H NMR (300 MHz, CDCl_3_): *δ* 7.96 (d, *J* = 8.5 Hz, 1H), 7.92 (d, *J* = 8.5 Hz, 1H), 7.57 (d, *J* = 4.8 Hz, 1H), 7.52 (d, *J* = 5.1 Hz, 1H), 7.35 (t, *J* = 7.5 Hz, 1H), 6.71 (s, 2H), 6.34 (d, *J* = 15.5 Hz, 1H), 5.97 (d, *J* = 5.4 Hz, 1H), 3.87 (d, *J* = 4.7 Hz, 9H), 3.50 (t, *J* = 7.1 Hz, 2H), 3.40–3.34 (m, 2H), 3.06 (s, 2H), 2.71 (s, 2H), 1.92 (s, 4H), 1.72–1.63 (m, 2H), 1.60–1.52 (m, 2H), 1.41 (s, 4H). ^13 ^C NMR (500 MHz, CDCl_3_): *δ* 166.02, 158.19, 153.38, 150.95, 147.08, 140.62, 139.54, 130.54, 128.48, 128.26, 123.69, 122.91, 120.36, 120.07, 115.73, 104.98, 60.93, 56.12, 49.24, 39.52, 33.74, 31.60, 29.62, 26.63, 26.55, 24.79, 23.01, 22.70. HRMS (ESI) *m/z* calcd for C_31_H_39_N_3_O_4_ [M + H]^+^ 518.3019; found 518.3019. HPLC (70% methanol in water with 0.5% H_3_PO_4_): *t*_R_ = 4.00 min, 96.23%.

*(E)-3-(3-hydroxyphenyl)-N-(6-((1,2,3,4-tetrahydroacridin-9-yl)amino)hexyl)acrylamide (**33**)*. Yellow powder, yield: 13%, mp 96–100 °C. ^1^H NMR (300 MHz, CDCl_3_): *δ* 8.00 (d, *J* = 7.8 Hz, 1H), 7.95 (d, *J* = 8.9 Hz, 1H), 7.60–7.52 (m, 1H), 7.45 (s, 1H), 7.41–7.35 (m, 1H), 7.19 (t, *J* = 7.9 Hz, 1H), 6.90 (d, *J* = 7.9 Hz, 2H), 6.80 (s, 1H), 5.88 (d, *J* = 15.4 Hz, 1H), 5.58 (s, 1H), 3.60–3.53 (m, 2H), 3.34–3.31 (m, 2H), 3.08 (s, 2H), 2.73 (s, 2H), 1.91 (s, 4H), 1.75–1.66 (m, 2H), 1.59–1.52 (m, 2H), 1.49 –1.36 (m, 4H). ^13 ^C NMR (500 MHz, CDCl_3_): *δ* 158.38, 141.02, 129.94, 128.98, 127.90, 123.93, 122.74, 120.46, 119.88, 119.75, 117.64, 48.91, 39.19, 33.14, 31.05, 29.73, 26.09, 26.01, 24.94, 22.92, 22.52. HRMS (ESI) *m/z* calcd for C_28_H_33_N_3_O_2_ [M + H]^+^ 444.265; found 444.2657. HPLC (70% methanol in water with 0.5% H_3_PO_4_): *t*_R_ = 2.45 min, 95.06%.

*(E)-3-(4-hydroxyphenyl)-N-(6-((1,2,3,4-tetrahydroacridin-9-yl)amino)hexyl)acrylamide (**34**)*. Yellow powder, yield: 15%, mp 108–110 °C. ^1^H NMR (300 MHz, CDCl_3_): *δ* 8.15 (s, 1H), 7.97–7.89 (m, 2H), 7.57–7.49 (m, 1H), 7.37–7.26 (m, 2H), 6.84 (d, *J* = 8.5 Hz, 1H), 6.14 (d, *J* = 11.6 Hz, 1H), 5.76 (s, 1H), 3.50 (t, *J* = 6.9 Hz, 2H), 3.40–3.14 (m, 2H), 3.05 (s, 2H), 2.70 (s, 2H), 2.05–1.74 (m, 4H), 1.72–1.60 (m, 2H), 1.57–1.48 (m, 2H), 1.45–1.29 (m, 4H). ^13 ^C NMR (500 MHz, CDCl_3_): *δ* 161.23, 158.42, 158.31, 150.95, 150.84, 147.26, 147.15, 129.50, 128.50, 128.35, 123.72, 122.88, 120.12, 116.25, 115.80, 49.28, 37.98, 33.74, 31.65, 29.50, 26.52, 26.50, 24.81, 23.03, 22.72. HRMS (ESI) *m/z* calcd for C_28_H_33_N_3_O_2_ [M + H]^+^ 444.2649; found 444.2649. HPLC (70% methanol in water with 0.5% H_3_PO_4_): *t*_R_ = 3.00 min, 95.43%.

*(E)-3-(4-phenoxyphenyl)-N-(6-((1,2,3,4-tetrahydroacridin-9-yl)amino)hexyl)acrylamide (**35**)*. Yellow powder, yield: 28%, mp 47–49 °C. ^1^H NMR (300 MHz, CDCl_3_): *δ* 7.99 (t, *J* = 7.8 Hz, 2H), 7.60–7.54 (m, 2H), 7.44 (d, *J* = 5.4 Hz, 3H), 7.42 (d, *J* = 1.9 Hz, 2H), 7.41 (d, *J* = 0.8 Hz, 1H), 7.39–7.38 (m, 1H), 7.36 (d, *J* = 1.6 Hz, 1H), 6.95 (d, *J* = 8.8 Hz, 2H), 6.29 (d, *J* = 15.5 Hz, 1H), 5.92 (d, *J* = 6.0 Hz, 1H), 5.09 (s, 2H), 3.54 (t, *J* = 7.1 Hz, 2H), 3.41–3.34 (m, 2H), 3.09 (s, 2H), 2.70 (s, 2H), 1.92 (t, *J* = 2.9 Hz, 4H), 1.69 (m, 2H), 1.60–1.54 (m, 2H), 1.43–1.39 (m, 4H). ^13 ^C NMR (500 MHz, CDCl_3_): *δ* 166.35, 160.06, 158.41, 150.90, 147.32, 140.47, 136.59, 129.33, 128.68, 128.51, 128.44, 128.15, 127.85, 127.48, 123.70, 122.90, 120.21, 118.53, 115.89, 115.17, 70.10, 49.33, 39.53, 33.89, 31.65, 29.68, 26.65, 26.59, 24.84, 23.06, 22.77. HRMS (ESI) *m/z* calcd for C_35_H_39_N_3_O_2_ [M + H]^+^ 534.3119; found 534.3119. HPLC (70% methanol in water with 0.5% H_3_PO_4_): *t*_R_ = 11.98 min, 97.02%.

*(E)-3-(4-benzyl-3-methoxyphenyl)-N-(6-((1,2,3,4-tetrahydroacridin-9-yl)amino)hexyl)acrylamide (**36**)*. Yellow powder, yield: 33%, mp 57–64 °C. ^1^H NMR (300 MHz, CDCl_3_): *δ* 8.11 (d, *J* = 8.3 Hz, 1H), 8.04 (d, *J* = 8.6 Hz, 1H), 7.62–7.48 (m, 3H), 7.46–7.31 (m, 6H), 7.12–6.99 (m, 2H), 6.86 (t, *J* = 5.7 Hz, 1H), 6.30 (d, *J* = 15.5 Hz, 1H), 5.86 (s, 1H), 5.17 (s, 2H), 3.89 (s, 3H), 3.64 (t, *J* = 6.9 Hz, 2H), 3.41–3.29 (m, 2H), 3.14 (s, 2H), 2.66 (s, 2H), 1.89 (s, 4H), 1.77–1.63 (m, 2H), 1.62–1.49 (m, 2H), 1.42 (s, 4H). ^13 ^C NMR (500 MHz, CDCl_3_): *δ* 166.29, 157.31, 151.55, 149.72, 149.67, 140.49, 136.71, 128.96, 128.62, 128.36, 128.00, 127.35, 127.26, 123.88, 123.09, 121.64, 119.55, 119.03, 115.14, 113.64, 110.47, 77.33, 56.03, 49.08, 45.99, 39.43, 31.49, 29.63, 26.54, 26.46, 24.68, 22.89, 22.45. HRMS (ESI) *m/z* calcd for C_36_H_41_N_3_O_3_ [M + H]^+^ 564.3221; found 564.3216. HPLC (70% methanol in water with 0.5% H_3_PO_4_): *t*_R_ = 9.66 min, 96.84%.

*(E)-3-(4-(4-chlorophenoxy)-3-methoxyphenyl)-N-(6-((1,2,3,4-tetrahydroacridin-9-yl)amino)hexyl)acrylamide (**37**)*. Yellow powder, yield: 17%, mp 68–70 °C. ^1^H NMR (300 MHz, CDCl_3_): *δ* 7.96 (t, *J* = 9.2 Hz, 2H), 7.59–7.46 (m, 2H), 7.33 (d, *J* = 8.6 Hz, 6H), 7.07–6.95 (m, 2H), 6.81 (d, *J* = 8.0 Hz, 1H), 6.28 (d, *J* = 15.3 Hz, 1H), 5.85 (s, 1H), 5.12 (s, 2H), 3.88 (s, 3H), 3.53 (t, *J* = 6.3 Hz, 2H), 3.38–3.32 (m, 2H), 3.06 (s, 2H), 2.68 (s, 2H), 1.90 (s, 4H), 1.66 (d, *J* = 5.5 Hz, 2H), 1.56 (d, *J* = 4.3 Hz, 2H), 1.41 (d, *J* = 22.8 Hz, 4H). ^13 ^C NMR (500 MHz, CDCl_3_): *δ* 166.17, 149.79, 149.43, 140.58, 135.25, 133.85, 128.98, 128.83, 128.77, 128.64, 127.40, 124.00, 123.91, 123.05, 121.59, 119.12, 119.05, 113.76, 110.49, 70.25, 56.02, 49.10, 39.45, 31.46, 30.23, 29.72, 26.52, 26.46, 24.68, 22.89, 22.45. HRMS (ESI) *m/z* calcd for C_36_H_40_ClN_3_O_3_ [M + H]^+^ 598.2834; found 598.2834. HPLC (70% methanol in water with 0.5% H_3_PO_4_): *t*_R_ = 16.25 min, 95.53%.

*(E)-3-(3-methoxy-4-(p-tolyloxy)phenyl)-N-(6-((1,2,3,4-tetrahydroacridin-9-yl)amino)hexyl)acrylamide (**38**)*. Yellow powder, yield: 52%, mp 63–65 °C. ^1^H NMR (300 MHz, CDCl_3_): *δ* 8.00 (d, *J* = 3.8 Hz, 1H), 7.97 (d, *J* = 3.9 Hz, 1H), 7.59–7.53 (m, 1H), 7.53–7.48 (m, 1H), 7.34 (d, *J* = 9.6 Hz, 1H), 7.30 (d, *J* = 7.9 Hz, 2H), 7.19–7.09 (m, 2H), 7.03–6.94 (m, 2H), 6.82 (d, *J* = 8.2 Hz, 1H), 6.30 (d, *J* = 15.5 Hz, 1H), 6.04 (t, *J* = 5.6 Hz, 1H), 5.12 (s, 2H), 3.86 (s, 3H), 3.56 (t, *J* = 7.1 Hz, 2H), 3.37–3.30 (m, 2H), 3.08 (s, 2H), 2.65 (s, 2H), 2.34 (s, 3H), 1.87 (s, 4H), 1.71–1.62 (m, 2H), 1.58–1.48 (m, 2H), 1.39 (d, *J* = 7.0 Hz, 4H). ^13 ^C NMR (500 MHz, CDCl_3_): *δ* 166.35, 156.69, 151.97, 149.73, 149.71, 140.44, 137.76, 133.68, 129.30, 129.27, 129.25, 128.29, 127.37, 126.58, 123.97, 123.20, 121.67, 119.17, 119.04, 114.59, 113.61, 110.44, 70.86, 56.01, 48.88, 39.40, 32.44, 31.44, 29.60, 26.49, 26.41, 24.65, 22.79, 22.24, 21.21. HRMS (ESI) *m/z* calcd for C_37_H_43_N_3_O_3_ [M + H]^+^ 578.3385; found 578.3385. HPLC (70% methanol in water with 0.5% H_3_PO_4_): *t*_R_ = 14.16 min, 96.28%.

### Biological determinations

#### In vitro inhibitory evaluations on AChE and BuChE

AChE (EC 3.1.1.7, Type VI-S, from electric eel, C3389; from human, C1682) and BuChE (EC 3.1.1.8, from equine serum, C0663; from human, B4186), 5,5'-dithiobis (2-nitrobenzoic acid) (DTNB, D218200), acetylthiocholine iodide (ATC, A5751), and butyrylthiocholine iodide (BTC, B3253) were obtained from Sigma-Aldrich (St. Louis, MO).

The inhibitory effects of the synthesised compounds in this paper were evaluated according to our previously reported method^28^. Briefly, AChE/BuChE stock solution was diluted to give 2.5 units/mL (for eeAChE, eqBuChE, and huAChE) or 0.5 units/mL for huBuChE. ATC/BTC iodide solution (0.075 M) was prepared in deionised water. DTNB solution (0.01 M) was prepared using water containing 0.15% (w/v) sodium bicarbonate. The assay buffer was prepared as follows: potassium dihydrogen phosphate (1.36 g, 10 mmol) was dissolved in 100 ml of water. The pH of the solution was then adjusted to 8.0 ± 0.1 with KOH. Stock solutions of the test samples were dissolved in ethanol to give a final concentration of 10^−4 ^M when diluted to the final volume of 3.32 ml. For each compound, a dilution series of at least five different concentrations (normally 10^−5^–10^−9 ^M) were prepared.

For measurement, a cuvette containing 3.0 ml of phosphate buffer, 100 µL of AChE or BuChE, 100 µL of DTNB, and 100 µL of the test compound solution were added in sequence. The reaction was initiated after adding 20 µL of ATC or BTC, and the solution was mixed immediately. Two minutes (eeAChE and eqBuChE) or 15 min (huAChE and huBuChE) after ATC or BTC addition, the absorption was measured at 25 °C (eeAChE and eqBuChE) or 37 °C (huAChE and huBuChE) at 412 nm, using a Shimadzu 160 spectrophotometer. For the reference value, 100 µL of water replaced the test compound solution. For the blank control, additionally 100 µL of water replaced the enzyme solution. The measurement for each concentration was assayed in triplicate. GraphPad Prism 5 was used for data processing. The inhibition curve was fitted by plotting percentage enzyme activity (100% for the reference) versus logarithm of test compound concentration. The IC_50_ values were calculated according to the curve, and the data were shown in mean ± SEM.

#### Kinetic studies of AChE inhibition

Kinetic studies were performed according to methods reported previously^45^. The concentrations of the substrate ATC or BTC were prepared as 25, 50, 90, 150, 226, and 452 µM. Different concentrations of compound **19** with 0, 1, 2, 4, 10 nM, were also prepared by stock solution. For measurement, the enzymatic reaction was extended to 20 min (huAChE and huBuChE) before the determination of the absorption. Graph Pad Prism 5 was applied for data processing. *V*_max_ and *K*_m_ values of the Michaelis–Menten kinetics were determined by nonlinear regression from substrate-velocity curves, while Lineweaver–Burk plots were fitted by using linear regression method.

#### Inhibition of self-induced Aβ_1–42_ aggregation

Inhibitory effects of the compounds on self-induced Aβ_1–42_ aggregation were evaluated using a Thioflavin T (ThT)-(T3516, Sigma-Aldrich, St. Louis, MO) binding assay according to the previously reported method^43^. Briefly, aliquots of 2.0 µL of Aβ_1–42_ (AS-64129–05 Anaspec Inc.) containing 2 mg/mL HFIP (1,1,1,3,3,3-hexafluoro-2-propanol, 52517, Sigma-Aldrich, St. Louis, MO) were stocked in DMSO. They were then diluted to the final concentration of 500 µM with 0.215 M sodium phosphate buffer (pH 8.0). Test compounds were dissolved in DMSO and then diluted by buffer to give a final concentration of 25 µM. The Aβ_1–42_ and the test sample solutions were incubated for 24 h at the room temperature. After the incubation, the test samples were diluted to a final volume of 150 µL with 50 mM glycine-NaOH buffer (pH 8.5) containing 5 mM Thioflavin T. Fluorescence intensity was read (excitation wavelength 450 nm, emission wavelength 485 nm) on a SpectraMax Paradigm Multimode Reader (Molecular Device).

The inhibitory rate of Aβ_1–42_ self-induced aggregation was calculated according to the following equation: (1-IFi/IFc) × 100%. IFi and IFc were the fluorescence intensities in the presence and absence of inhibitors, respectively, after subtracting the background fluorescence of the 5 mM Thioflavin T solution. Each measurement was repeated in triplicate. The inhibitory rate of the test compound was shown in mean ± SD.

#### Behavioural studies

Behavioural studies were carried out according to the method reported previously^46^. Briefly, the adult male ICR mice (8–10 weeks old, weight 20–25 g) were obtained from the Yangzhou University Medical Center (Yangzhou, China). Scopolamine hydrobromide was purchased from Aladdin Reagents (H1507073, Shanghai, China). Tacrine that was synthesised in our lab with >95% purity was used as the positive control.

The mice were separated into six groups as follows: (i) vehicle as a blank control group, (ii) scopolamine as a model group, (iii) tacrine plus scopolamine as a positive control, (iv) compound **19** plus scopolamine as a test group, (v) compound **27** plus scopolamine as a test group; and (vi) compound **30** plus scopolamine as a test group. Each group contains six mice. Tacrine and the synthesised compounds were orally administered (20 µmol/kg body weight) to mice in groups (iii), (iv), (v), and (vi), 30 min before the ip administration of scopolamine (1 mg/kg) or saline for 10 consecutive days.

The cognitive function of the mice was evaluated by the Morris water maze test, determined by analysis-management system (Panlab SMART 3.0, America), according to the method previously described^43^. The maze was placed in a lit room with visual cues at 25 °C. An escape platform (10 cm diameter) was at the centre of one quadrant of the circular pool (120 cm diameter, 60 cm height) with a depth of 40 cm water. For measurement of the cognitive function, each mouse included 5 days of learning and memory training, followed by a probe trial on day 6. The starting positions faced to the pool wall and were pseudorandomised for each trial. For the cognitive evaluation, each mouse was individually evaluated on both visible-platform (days 1–2) and hidden-platform (days 3–5) versions of the water maze. All mice received nonspatial pretraining during the first two training days, which prepared them for the subsequent spatial learning test. During the two days, mice were trained to find the platform that was labelled by a small flag (5 cm tall). The hidden-platform version was used to determine the retention of memory to find the platform. During the hidden-platform training trials, the escape platform was placed 1 cm below the surface of the water. On each day, the animal was subjected to two trials, each of which lasted for 90 s. The time for the mouse to find the platform (a successful escape) was recorded. If a mouse failed to reach the platform within 90 s, the test was terminated and the animal was gently navigated to the platform by hand. Whether a mouse was successful or failed to reach the platform within 90 s, it was kept on the platform for 30 s. On the last day (day 6), the platform was removed from its location and the animals were given a probe trial in which they had 90 s to search for the platform. The time taken to reach the missing platform and the number of times the animals crossed the platform location were recorded.

Data for the time of escape latency, the trajectory travelled, and the number of platform location crossings were recorded by Panlab SMART 3.0 and processed by Graphpad Prism 5.

#### Hepatotoxicity studies

Hepatotoxicity was evaluated according to the method previously described^47^ by using adult male ICR mice (8–10 weeks old, weighing 20–25 g) obtained from the Yangzhou University Medicine Centre (Yangzhou, China). Tacrine and the test compounds were dissolved in a sodium carboxymethyl cellulose (CMC-Na) solution (0.5 g CMC-Na in 100 ml distilled water). Concentration of 3 mg/100 g body wt. of tacrine, corresponding to 151.5 µmol/kg body wt., was administered intragastrically (ig). Equimolar dose of test compounds to that of tacrine was administered ig. 8, 22, and 36 h after the administration, heparinised serum was collected from the retrobulbar plexus and subjected to hepatotoxicity evaluation. The activity of AST and ALT, two indicators of liver damage, was determined using corresponding assay kit (EF551 and EF550 for ALT, EH027, and EF548 for AST, Wako, Japan). The data were processed by Biochemical Analyzer (HITACHI 7020, Japan).

One hour after the collection of retrobulbar blood, mice were sacrificed and livers were harvested for morphological studies by using immunohistochemical method. Two 3 mm sections of each liver extending from the hilus to the margin of the left lateral lobe were isolated by Ultra-Thin Semiautomatic Microtome (Leica RM2245, Germany) and immediately placed in 10% buffered formaldehyde, fixed for two days, and embedded together in one paraffin block by using Paraffin Embedding Station (Leica EG1150H, Germany). Subsequently, 5 µm sections were prepared from these paraffin blocks. They were deparaffinated and stained with hematoxylin and eosin or by means of the periodic acid-Schiff procedure for glycogen.

### Molecular docking studies

The molecular docking was completed by CDOCKER module implemented in Discovery Studio^48^. The co-crystal structures of huAChE and huBuChE with small molecular ligands were downloaded from Protein Data Bank (PDB, ID: 4EY7, 4TPK). The structures were initially processed by “Prepare Protein” module in DS to give the structures suitable for docking. Missed sidechains of the proteins were added and the water molecules were removed, then the structures were protonated at pH 7.4. “Prepare Ligands” module in DS was applied for the structural preparation of the test compounds, which were then protonated at pH 7.4. The resulted molecules were subsequently minimised by “Minimise Ligands” module. The “Smart Minimiser” algorithm was used to carry out the minimisation, with max steps set to 2000, RMS Gradient set to 0.01. Other parameters were set as default.

For molecular docking, the binding site was defined as a site sphere (in 10 Å radius) around the original ligands in the co-crystal structures. The simulated annealing parameters were set as follows: heating steps and cooling steps were set to 2000 and 5000, respectively, while heating and cooling temperature were set to 700, and 310, respectively. Other parameters were kept as default. Ten top-ranked conformations for each docked compound were retained for binding pattern analysis, which were visualised and depicted in DS.

## Results and discussions

### Compound design and chemistry

The cinnamic acid is a naturally originated compound with diverse biological activity. We notice that several derivatives of cinnamic acid, such as ferulic acid, caffeic acid, are reported to benefit the treatment of AD for many reasons. More importantly, we observe that the cinnamic acid moiety can serve as a good scaffold to insert into the narrow groove of the AChE active site, forming intermolecular interactions with residues in the PAS. Therefore, we consider that cinnamic acid moiety is a good fragment for designing CAS-PAS dual site ChEs inhibitor. However, as a PAS binder, the structural modification of this moiety is not fully analysed previously. Herein, we designed a series of tacrine-cinnamic acid hybrids, and discussed the SAR for these compounds as dual site ChEs inhibitors.

**Scheme 1.**
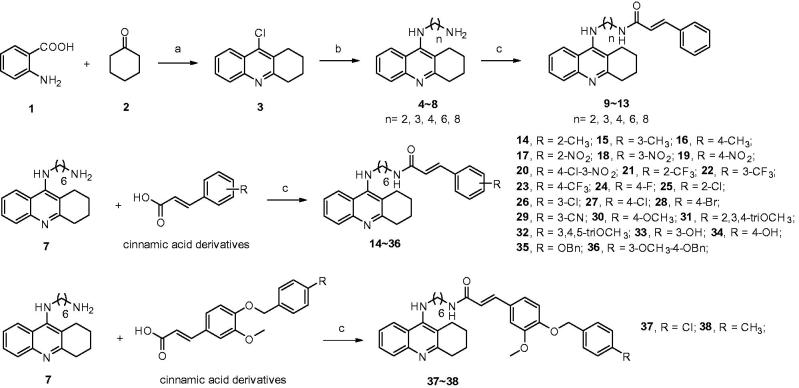
Synthetic route of tacrine-cinnamic acid hybrids. Reagents and conditions: (a) POCl_3_, reflux, 3 h; (b) pentanol, NH_2_(CH_2_)_n_NH_2_, NaI, reflux, 18 h; (c) PyBop, DIPEA, anhydrous CH_2_Cl_2_, room temp, 24 h.

The synthesis of the tacrine-cinnamic acid hybrids is described in Scheme 1. Anthranilic acid **1** was condensed with cyclohexanone **2** to yield chloro acridine **3**^38^^,^^39^. Treatment of **3** with different diamine led to **4**–**8**, which were condensed with cinnamic acid to result in the target compounds **9**–**13**. Compound **7** was condensed with different cinnamic acid analogues to obtain target molecule **14**–**38**.

### ChEs inhibitory activity and SAR analysis

The inhibitory effects on ChEs of all the target compounds were determined according to Ellman’s method^40^. AChE from electrophorus electricus (eeAChE) and BuChE from equine serum (eqBuChE) were used in the assays. The activity was quantified by IC_50_ values (Table 1). All the compounds exhibited potent inhibitory activities on ChEs, with IC_50_ values in the nanomolar range. We firstly evaluated the optimal linker between tacrine^41^^,^^42^ and cinnamic acid moiety, by introducing different diamines (compound **9**–**13**). When *n* = 2–4, compounds showed low activities on AChE, but when the diamine linker was extended to six or eight carbon atoms, compounds showed much improved activities (AChE IC_50_ = 26.5 ± 10.7 and 22.8 ± 9.2 nM for **12** and **13**, respectively). We also observed that compound **10** and **11** exhibited high selectivity on BuChE (selective index =28.7 and 71.17, respectively), indicating that they may serve as lead compounds for the discovery of selective BuChE inhibitors. As **12** showed comparable activity to **13**, while its molecular weight was lower than **13**, the hexamethylendiamine was considered to be the optimal linker for further structural modifications.

Next, we examined the optimal substituents on cinnamic acid moiety. Methyl substitution at *meta*- or *ortho*- position of cinnamic acid (**14** and **15**, respectively) led to the reduced activities compared to compound **12**, but the *para*-substituted compound **16** showed comparable activity to **12**. We further examined the inductive effects of the substituents on the ChEs inhibitory activity. The nitro group was introduced to act as an electron-withdrawing group. Data showed that the nitro group could remarkably improve the activity of the compounds. The *para*-substituted **19** was the most potent compound (AChE IC_50_ = 2.7 ± 0.4 nM, BuChE IC_50_ = 6.5 ± 0.6 nM). When the nitro group was replaced by -CF_3_, compounds were less potent than **12**, except for the para-substituted compound **23**. Interestingly, the effects of methyl, nitro and -CF_3_ substitution on ChEs activity showed a consistent manner: for AChE, the activity was *para*- > *meta*- > *ortho*-, while it showed an opposite manner on BuChE. The results indicated that the *para*-position was optimal for the design of potent AChE inhibitor.

We subsequently designed compounds with halogen substitution. When substituted by Cl (**25**–**27**), the impact was very similar to the above-mentioned results. When substituted by different halogens, the activities on AChE and BuChE were 4-Cl^27^ > 4-Br^28^ > 4-F^24^.

Next, we synthesised several methoxyl-substituted analogues to evaluate the impact of electro-donating effect on activity. For mono-substituted compound **30**, the activities on both AChE and BuChE were obviously improved (AChE IC_50_ = 3.7 ± 1.5 nM, BuChE IC_50_ = 22.5 ± 5.9 nM) compared **12**. For multi-substituted compounds (**31** and **32**), the 3,4,5-triOCH_3_ was much more active than the 2,3,4-triOCH_3_.

We subsequently introduced the hydroxy group (**33**–**34**) to the cinnamic acid moiety. Such substitution was favourable for AChE inhibition, the two compounds exhibited a good selectivity on AChE. We next introduced benzyloxy group at the *para*-position of the cinnamic acid moiety^35^. Compared to **12**, the activities of the three compounds on AChE were remarkably reduced, while the BuChE activities were maintained. In addition, we synthesised three derivatives with 3-OMe-4-OBn substitution (**36**–**38**) on the cinnamic acid moiety, they also exhibited much reduced activities against ChEs. As the binding site of BuChE was larger than AChE, we inferred that the large benzyloxy group can be tolerated by BuChE, while it was repelled by AChE.

To further validated the inhibitory activities of the target compounds on human ChEs, the active representatives, **19**, **27**, and **30**, were selected for validation (Table 1). **19** exhibited huAChE IC_50_ = 10.2 ± 1.2 nM, huBuChE IC_50_ = 6.3 ± 0.3 nM; **27** exhibited huAChE IC_50_ = 16.5 ± 1.7 nM, huBuChE IC_50_ = 5.7 ± 0.3 nM. **30** exhibited huAChE IC_50_ = 15.3 ± 1.8 nM, huBuChE IC_50_ = 8.0 ± 1.1 nM. The activities were very similar to that in the eeAChE or eqBuChE assays. Therefore, we concluded that the synthesised compounds can efficiently inhibit the activities of human ChEs, further confirmed their activities as ChEs inhibitors.

### Kinetic study of huAChE and huBuChE inhibition

**Figure 1.**
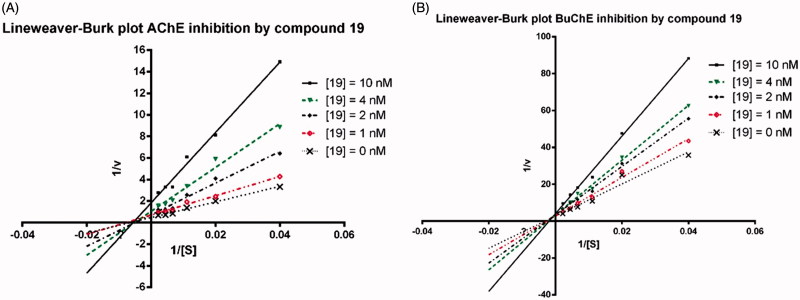
Lineweaver–Burk plots resulting from subvelocity curves of huAChE and huBuChE activity with different substrate concentrations (25–450 μM) in the absence and presence of 1, 2, 4, 10 nM of **19**.

Next, we selected compound **19**, which exhibited the very potent ChEs inhibitory activity, to analyse its binding manner with huAChE and huBuChE by using kinetic studies as described previously^43^. Lineweaver–Burk reciprocal plots were applied to describe the type of inhibition by **19**. For AChE inhibition as shown in Figure 1(A), when increase the concentration of **19** (1, 2, 4, and 10 nM), both the slopes and the intercepts were increased, indicating a decreased *V*_max_ and a higher *K*_m_. The intersection point located at the *Y*-axis, suggesting a noncompetitive binding manner of **19** on huAChE. For BuChE inhibition (Figure 1(B)), the intersection point located at the second quadrant, indicating a mixed-type inhibition of **19** on huBuChE. The detailed values of *V*_max_ and *K*_m_ at different concentrations in the kinetic studies are summarised in Table 2.

**Table 1.** AChE and BuChE inhibitory effects (shown as IC_50_ values), and selectivity index (SI) of the synthesised compounds.


Compound	*R*	IC_50_ (nM) ± SEM^a^		SI^d^
		AChE^b^	BuChE^c^	
**9**	–	328.2 ± 121.7	69.1 ± 27.3	0.2
**10**	–	100.3 ± 5.2	3.5 ± 0.4	0.03
**11**	–	209.3 ± 76.0	2.9 ± 0.4	0.01
**12**	–	26.5 ± 10.7	34.4 ± 16.6	1.3
**13**	–	22.8 ± 9.2	7.8 ± 4.7	0.3
**14**	2-CH_3_	36.0 ± 12.3	6.0 ± 1.5	0.2
**15**	3-CH_3_	53.1 ± 6.9	37.3 ± 7.2	0.7
**16**	4-CH_3_	22.0 ± 3.7	54.4 ± 11.7	2.5
**17**	2-NO_2_	17.5 ± 3.7	3.6 ± 0.3	0.2
**18**	3-NO_2_	3.6 ± 0.5	6.8 ± 0.6	1.9
**19**	4-NO_2_	2.7 ± 0.4 10.2 ± 1.2^d^	6.5 ± 0.6 6.3 ± 0.3^e^	2.40.6
**20**	4-Cl-3-NO_2_	6.2 ± 2.0	11.1 ± 2.2	1.8
**21**	2-CF_3_	41.7 ± 8.5	2.4 ± 0.6	0.04
**22**	3-CF_3_	61.3 ± 16.7	81.1 ± 15.7	1.3
**23**	4-CF_3_	8.1 ± 0.3	16.4 ± 1.8	2.0
**24**	4-F	15.4 ± 3.3	65.9 ± 21.4	4.3
**25**	2-Cl	14.2 ± 3.2	8.7 ± 2.7	0.6
**26**	3-Cl	16.2 ± 6.5	9.2 ± 2.5	0.6
**27**	4-Cl	6.9 ± 1.2 16.5 ± 1.7^d^	12.9 ± 1.75.7 ± 0.3^d^	1.90.3
**28**	4-Br	19.1 ± 2.0	29.9 ± 1.8	1.6
**29**	3-CN	5.1 ± 0.8	68.5 ± 13.6	13.4
**30**	4-OCH_3_	3.7 ± 1.5 15.3 ± 1.8^d^	22.5 ± 5.9 8.0 ± 1.1^e^	6.10.5
**31**	2,3,4-tri-OCH_3_	24.1 ± 8.1	8.1 ± 2.0	0.3
**32**	3,4,5-tri-OCH_3_	6.4 ± 2.2	3.4 ± 0.4	0.5
**33**	3-OH	6.4 ± 2.1	25.1 ± 6.5	3.9
**34**	4-OH	2.2 ± 0.3	43.5 ± 9.3	19.8
**35**	4-OBn	128.0 ± 32.3	29.0 ± 11.7	0.2
**36**	3-OMe-4-OBn	86.3 ± 30.0	29.8 ± 7.3	0.3
**37**	Cl	104.3 ± 26.4	22.6 ± 7.1	4.610.2
**38**	-CH_3_	127.8 ± 13.3	58.4 ± 7.5	0.5
Tacrine	–	69.8 ± 11.1	10.6 ± 1.1	0.2

a Concentration of the compound required for 50% inactivation of ChEs, data were shown in mean ± SEM of three experiments.

b AChE (EC 3.1.1.7) from electric eel.

c BuChE (EC 3.1.1.8) from horse serum.

d AChE (EC 3.1.1.7) from human.

e BuChE (EC 3.1.1.8) from human.

^f^Selectivity index (SI) = BuChE IC_50_/AChE IC_50_.

**Table 2.** The *V*_max_ and *K*_m_ values for compound **19** in kinetic studies.

Concentration (nM)	*V*_max_ (μM/min)	*K*_m_ (μM)	R square
**19** on AChE^a^			
0	2.0 ± 0.2	131.2 ± 34.0	0.98
1	1.4 ± 0.1	115.9 ± 28.6	0.98
2	1.2 ± 0.2	141.4 ± 60.8	0.94
4	0.9 ± 0.1	169.2 ± 45.8	0.98
10	0.5 ± 0.1	136.1 ± 40.6	0.97
**19** on BuChE^b^			
0	0.47 ± 0.05	420.6 ± 84.6	0.99
1	0.40 ± 0.03	406.1 ± 55.9	0.99
2	0.25 ± 0.01	299.6 ± 40.8	0.99
4	0.44 ± 0.08	741.4 ± 208.2	0.99
10	0.19 ± 0.01	355.1 ± 47.8	0.99

Data are shown in mean ± SD of three experiments.

a AChE (EC 3.1.1.7) from human.

b BuChE (EC 3.1.1.8) from human.

### Binding mode analysis by molecular modelling

**Figure 2.**
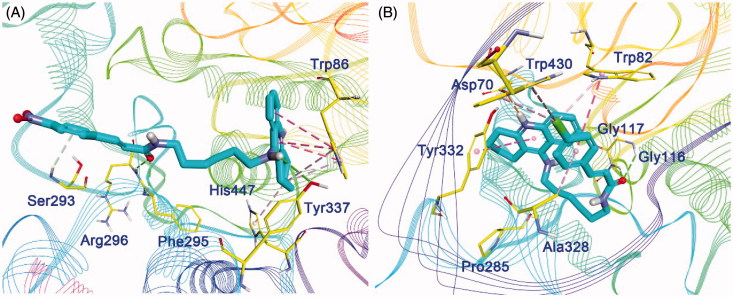
Binding mode prediction of the representative compounds with ChEs. Visualisation of **19** on huAChE (A, PDB id: 4EY7), and visualisation of **27** on huBuChE (B, PDB id: 4TPK) were shown in the figure. Compounds were shown in stick mode coloured in blue. Key residues were labelled as thin stick mode in yellow. Intermolecular interactions were described as dot lines in different colours according to the type of the interaction: green, hydrogen bond; light green, hydrophobic contact; purple, π–π stacking; pink, π-alkyl contact.

**Figure 3.**
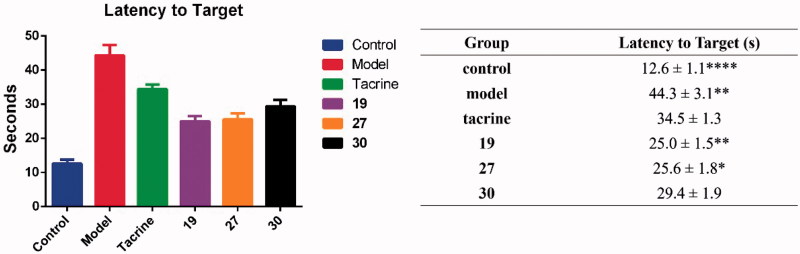
Effects of oral administration of tacrine (15 mg/kg), **19**, **27**, and **30** (15 mg/kg) on scopolamine-induced cognitive impairment in ICR mice determined by the Morris water maze test. Tacrine (15 mg/kg) was used as positive control. Data are presented as the mean ± SEM (*n* = 6; *p < .05, **p < .01, ****p < .0001 vs. tacrine group).

To further investigate the binding pattern of the synthesised compounds with ChEs, we next performed molecular docking studies by using Discovery Studio (DS) (version 3.0, BIOVIA) (Figure 2). Bind mode of huAChE-**19** and huBuChE-**27** were analysed. The two compounds were selected as representatives because they exhibited very potent activity on AChE or BuChE. As shown in Figure 3(A), **19** simultaneously occupied both the CAS and PAS of AChE. The 1,2,3,4-tetrahydroacridin core located at the CAS, and formed π–π stacking interactions with Trp86 and His447. The -NH- group on 1,2,3,4-tetrahydroacridin ring interacted with the backbone of Tyr337 through a hydrogen bond. The carbonyl group of the cinnamic acid moiety formed two hydrogen bonds with the sidechain of Phe295 and Arg296. Such polar interactions stabilised the binding pattern, resulting in the location of cinnamic acid moiety in the PAS site. The phenyl ring interacted with multiple residues through van der Waals interactions, such as Trp286, Leu289, Ser293, and Val294.

Compared to the linear conformation of **19** when it bound to huAChE, compound **27** exhibited a U-shaped conformation (Figure 3(B)), which was obviously different from **19**. Different from the narrow and long binding site of AChE, BuChE contains a much larger and broader binding site, therefore, the U-shaped conformation of **27** can better occupy the active site of BuChE. Such phenomenon also indicated the importance of the flexible linker in this series of compounds. The protonated nitrogen atom on 1,2,3,4-tetrahydroacridin ring formed a salt bridge with the sidechain of Asp70. The tricyclic ring also interacted with the sidechain of Tyr332 through π–π stacking contact. The oxygen atom of amide moiety formed two hydrogen-bonds with Gly116 and Gly117. For the cinnamic acid moiety, the phenyl ring interacted with Trp82 and Ala328 through π–π stacking and π-alkyl contact, respectively. The chlorine atom formed additional π-alkyl contacts with Trp430, which further improved the binding affinity of **27**. Multiple van der Waals contacts were observed between compound **27** and different residues such as Ser120, Leu286, Trp231, Phe398, and His438, providing strong binding affinity.

### Inhibition of self-induced Aβ_1–42_ aggregation by selected compounds

Compound **19**, **27,** and **30** that showed potent activity on ChEs inhibition were further evaluated for their inhibitory capacity on self-induced Aβ_1–42_ aggregation based on a thioflavin T-based fluorometric assay. Under the concentration of 25 µM, the three compounds inhibited the aggregation of Aβ_1–42_ with the inhibitory rate 31.82, 42.22, and 34.57%, respectively (Table 3). 25 µM of Resveratrol was used a reference compound, which showed 30.36% inhibitory rate. The results suggested that these ChEs inhibitors had the multi-target potential in the treatment of AD. Therefore, the *in vivo* activities of the three compounds were further evaluated.

**Table 3.** Inhibition of self-induced Aβ_1–42_ aggregation and anti-proliferative activities of the synthesised compounds.

Inhibitory rate of compounds (25 μM) on self-induced Aβ_1-42_ aggregation (%)				
Compound	**19**	**27**	**30**	Resveratrol
Inhibitory rate	31.82	42.22	34.57	30.36

### Behavioural studies

**Figure 4.**
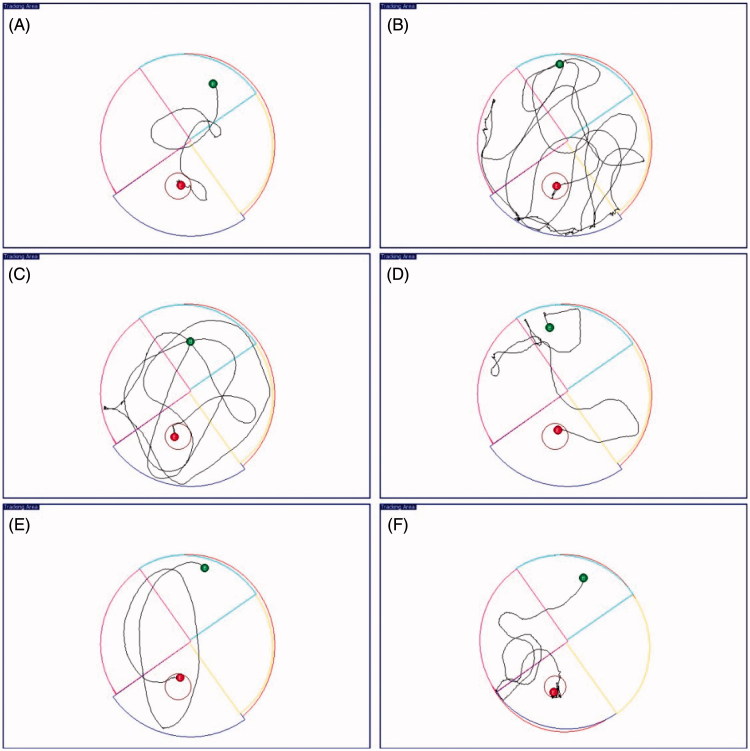
The trajectories of mice in control (A), model (B), tacrine (C), **19** (D), **27** (E), and **30** (F) group in the Morris water maze test.

To further investigate the ability of the synthesised compound to ameliorate the cognitive ability, three representatives, **19**, **27**, and **30**, were selected for *in vivo* behavioural analysis by using a Morris water maze test in scopolamine-induced cognition-impaired adult ICR mice (male mice, 8–10 weeks old, weight 20–25 g). Tacrine (30 mg/kg body weight) was used as positive control. After the treatment of the test compounds, the mean escape latency values of all the groups were shown in Figure 3 (the latency values of the mice in the training process are shown in Table S1). It was clear that administration of scopolamine led to a remarkable delay of the latency to target (12.6 ± 1.1 s vs. 44.3 ± 3.1 s) as compared to the control group. Tacrine reduced this time to 34.5 ± 1.3 s. All of the three synthesised compounds exhibited much improved cognitive function in the ICR mice, as the time of latency to target obviously reduced compared to tacrine. Among them, compound **19** and **27** showed comparable performance (25.0 ± 1.5 s, **p < .01, 25.6 ± 1.8 s, *p < .05, respectively), while **30** was slightly less positive (29.4 ± 1.9 s). The data of latency to target were also supported by the analysis of the trajectories of the mice in each group. For the mice in scopolamine model group (Figure 4(B)), the trajectory was very long and disordered as compared to the control group (Figure 4(A)). The performance of tacrine group was improved, but still much longer than the control group (Figure 4(C)). For mice treated with **19**, **27**, and **30** (Figure 4(D–F)), they showed much shortened distances as compared to tacrine group, with a similar orientation and distance to that of the normal mice. The results indicated that the cognitive function of mice in these three groups were much recovered.

The results confirmed that the synthesised compounds were *in vivo* active and had the potential as anti-AD agents through ameliorating the cognitive function. Besides, it also indicated that the electron-withdrawing groups might play significant role for the activity, as the nitro as R group showed the best performance. Halogen substitution at this position was also acceptable, while electron-donating groups reduced the activity of the compounds.

### Hepatotoxicity studies

**Figure 5.**
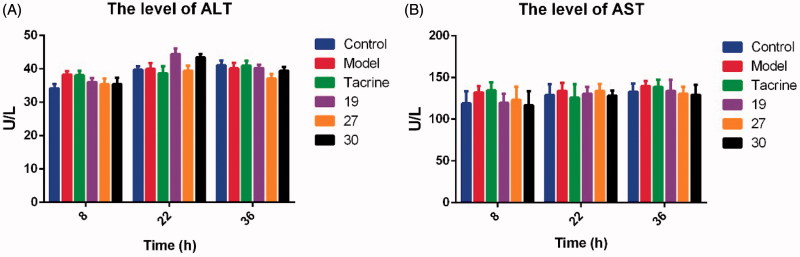
The determination of ALT (A) and AST (B) level after the treatment of test compounds. Values are expressed as mean ± SEM (*n* = 6).

**Figure 6.**
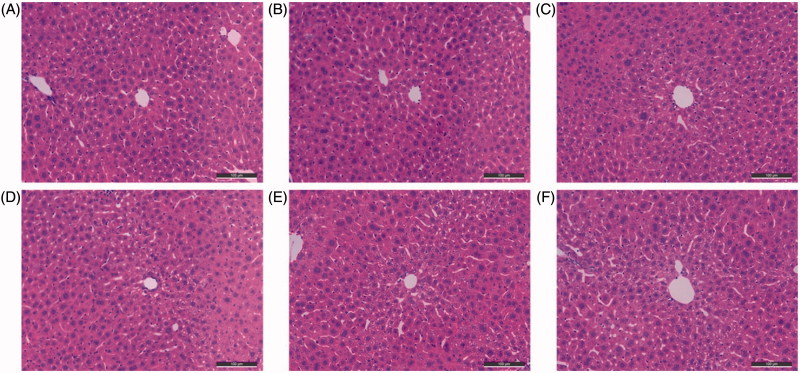
The histomorphological appearance of livers of male mice after treatment with the solvent only (control, A), or 36 h after administration of scopolamine (B), tacrine (C), **19** (D), **27** (E) and **30** (F). HE staining, original magnification ×200.

For preliminary safety evaluation, we next investigated the possible drug-induced hepatotoxicity of **19**, **27**, and **30** by determining the levels of alanine aminotransferase (ALT) and aspartate aminotransferase (AST), two known markers of liver damage (Table 4 and Figure 5). Heparinised serum was collected after the treatment of the compounds at 8, 22, and 36 h. Generally, all the three synthesised compounds did not show remarkable hepatotoxicity, as the levels of ALT and AST were comparable to those from the control group at the three time points. Therefore, these compounds showed preliminary safety as expected. In addition, the morphologic results of the three compounds by immunohistochemical staining were in accordance with the ALT and AST data. Treatment of tacrine (Figure 6(C)), **19** (Figure 6(D)), **27** (Figure 6(E)) or **30** (Figure 6(F)) did not cause remarkable morphologic changes in liver compared to the control group (Figure 6(A)). Taken together, our synthesised compounds were safe for further development.

**Table 4.** The determination of ALT and AST level (U/L) after the administration of test compounds.

Group	8 h	22 h	36 h
	ALT (U/L)		
**Control**	31.2 ± 3.2	39.7 ± 2.6	41.1 ± 3.4
Model	38.3 ± 2.5	39.9 ± 4.4	40.1 ± 4.1
Tacrine	38.0 ± 3.5	38.7 ± 5.2	40.9 ± 3.5
**19**	36.0 ± 2.9	44.4 ± 3.9	40.2 ± 2.5
**27**	35.4 ± 4.2	39.4 ± 3.7	37.1 ± 3.5
**30**	35.4 ± 4.7	43.4 ± 2.5	39.4 ± 2.7
	AST (U/L)		
Control	118.9 ± 14.4	128.9 ± 13.1	132.9 ± 9.9
Model	132.2 ± 7.6	134.1 ± 9.6	139.7 ± 5.9
Tacrine	134.5 ± 9.9	125.8 ± 16.2	138.7 ± 8.5
**19**	119.6 ± 10.8	130.6 ± 8.2	133.9 ± 13.5
**27**	123.3 ± 15.6	133.9 ± 8.4	130.6 ± 8.3
**30**	116.7 ± 16.9	128.3 ± 5.9	129.2 ± 12.1

Tacrine (30 mg/kg) was used as the reference compound. Values were expressed as the Mean ± SD (*n* = 6).

## Conclusions

In the present study, with the aim to identify multi-target directed compounds as new anti-AD agents, a series of tacrine-cinnamic acid hybrids were synthesised. Most of the compounds potently inhibited both AChE and BuChE, with the IC_50_ values in the nanomolar range. The SAR study indicated that the optimal linker between the tacrine and cinnamic acid moiety was the six-carbon alkyl chain. Extension the length of the linker, or introduction of bulky group such as benzyl group to the cinnamic acid moiety, resulted in much reduced ChEs inhibitory effects. Our findings further provide structural information in designing potent ChEIs. The binding manner of compound **19** to huAChE and huBuChE was also analysed by kinetic and molecular docking studies. The representatives, compound **19**, **27**, and **30**, effectively inhibited the self-induced Aβ_1–42_ aggregation. Subsequent *in vivo* evaluation of the three compounds showed that they remarkably reduced the scopolamine-induced cognitive impairment in the Morris water maze test. In addition, the compounds exhibited preliminary safety in hepatotoxicity studies, without improving the level of ALT and AST. Our findings enlarge the SAR of tacrine-based hybrids, and provide promising lead compounds for further optimisation of new therapeutic agents on AD.

## Supplementary Material

IENZ_1412314_Supplementary_Material.pdf
