# Supplementary material for: Synthesis and bioevaluation of new tacrine-cinnamic acid hybrids as cholinesterase inhibitors against Alzheimer’s disease
Source: J Enzyme Inhib Med Chem. 2017 Dec 27;33(1):290–302. doi: 10.1080/14756366.2017.1412314 (PMC7011792; doi:10.1080/14756366.2017.1412314)
Supplement: IENZ_1412314_Supplementary_Material.pdf [file IENZ_A_1412314_SM0766.pdf]

Synthesis and Bioevaluation of New Tacrine-Cinnamic Acid Hybrids as Cholinesterase Inhibitors against Alzheimer's Disease

Yao Chen [a,d,e], Jie Zhu [b], Jun Mo [b], Hongyu Yang [b], Xueyang Jiang [f], Hongzhi Lin [b], Kai Gu [b], Yuqiong Pei [a], Liang Wu [a], Renxiang Tan [e], Jing Hou [c], Jingyi Chen [c], Yang Lv [a], Yaoyao Bian [c,*], Haopeng Sun [b,*]

[a] *School of Pharmacy, Nanjing University of Chinese Medicine, Nanjing, 210023, China;*

[b] *Department of Medicinal Chemistry, China Pharmaceutical University, Nanjing, 210009, China;*

[c] *School of Nursing, Nanjing University of Chinese Medicine, Nanjing, 210023, China;*

[d] *Jiangsu Collaborative Innovation Center of Chinese Medicinal Resources Industrialization, Nanjing University of Chinese Medicine, Nanjing, 210023, China;*

[e] *State Key Laboratory Cultivation Base for TCM Quality and Efficacy, Nanjing University of Chinese Medicine, Nanjing, 210023, China;*

[f] *Key Laboratory of Biomedical Functional Materials, School of Science, China Pharmaceutical University, Nanjing 211198, China*

Corresponding author

Haopeng Sun: Tel: +86-25-85863169, E-mail: sunhaopeng@163.com;

Yaoyao Bian: Tel: +86-15952007562, E-mail: 1691@163.com

9 ^{1}H-NMR spectrum

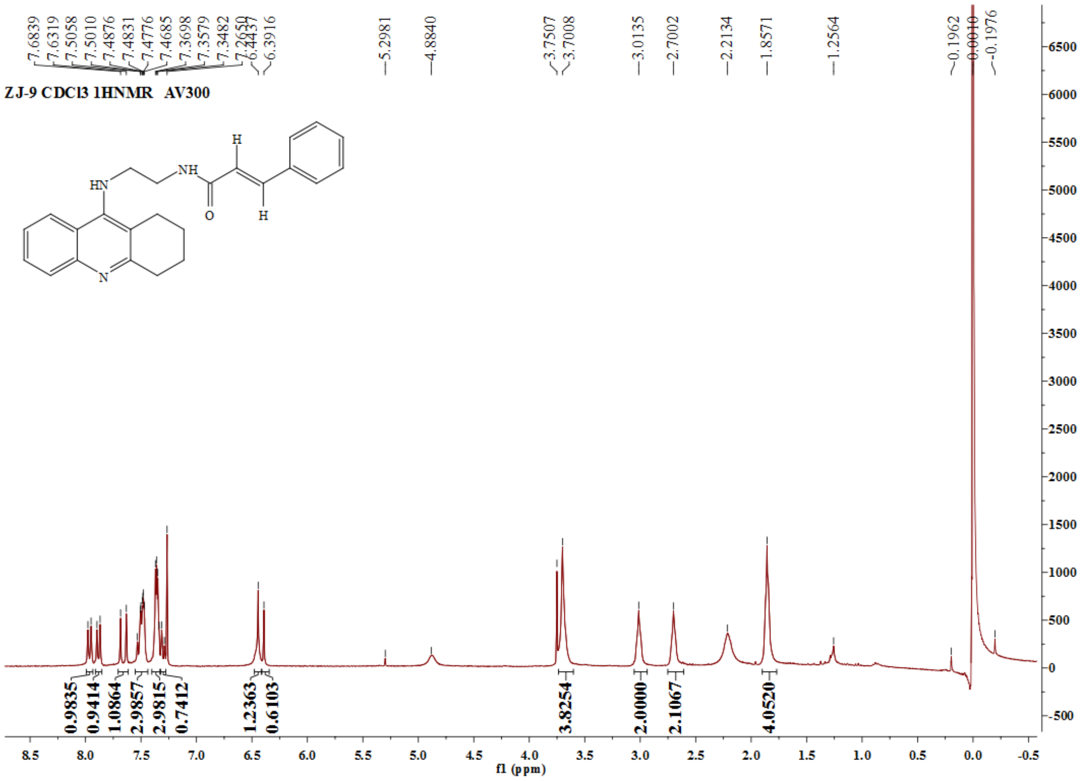

9 ^{13}C-NMR spectrum

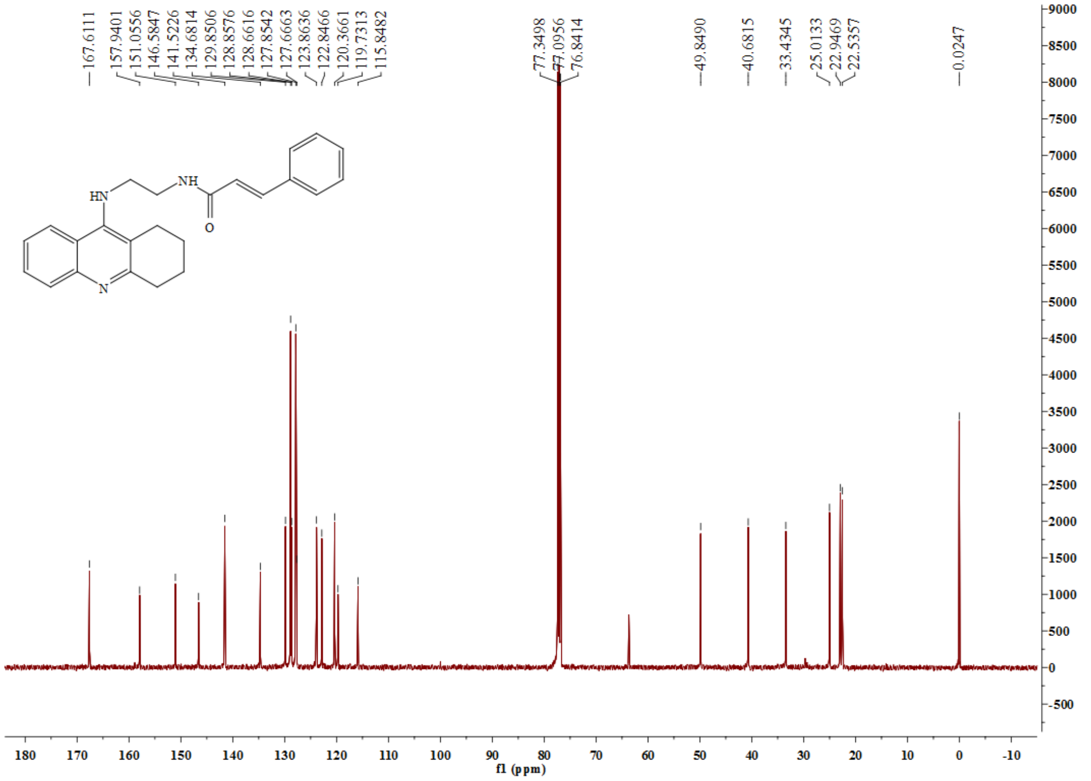

10 ^{1}H-NMR spectrum

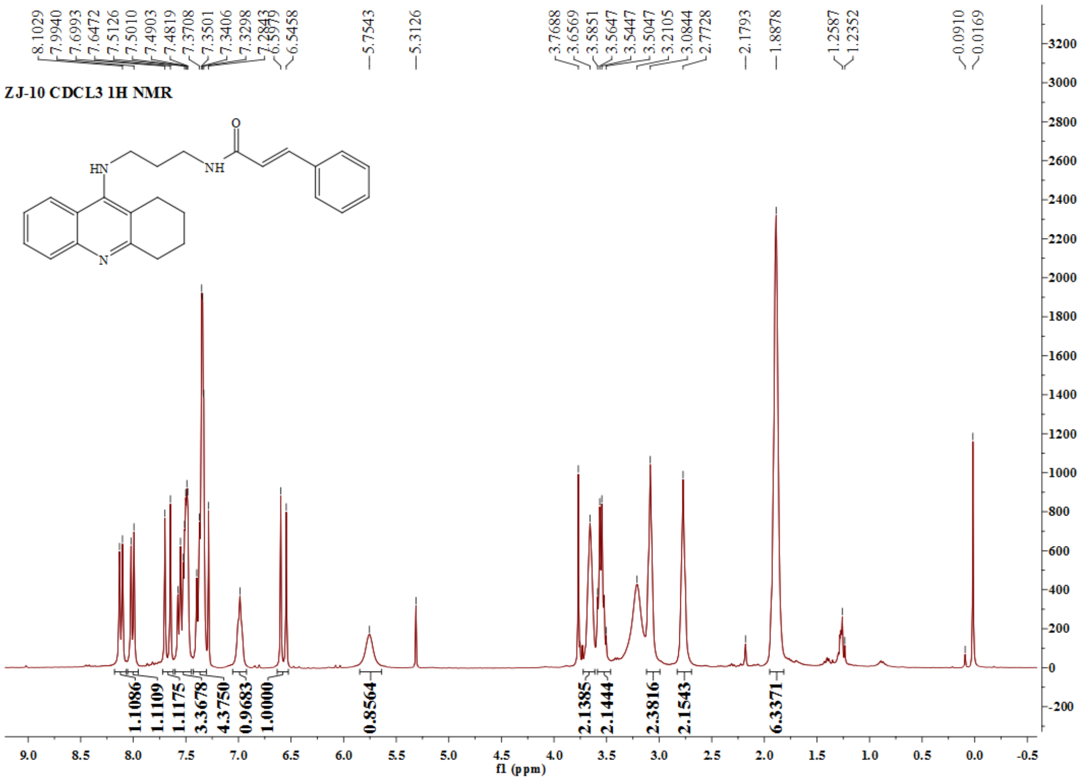

10 ^{13}C-NMR spectrum

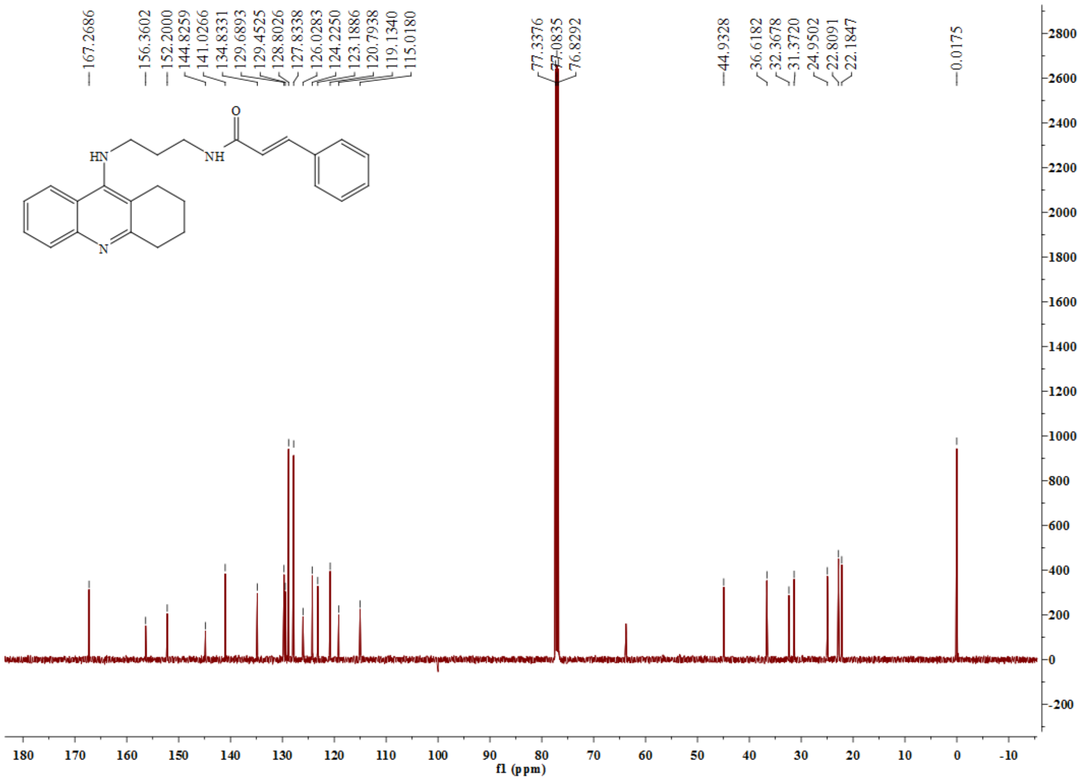

11 ^{1}H-NMR spectrum

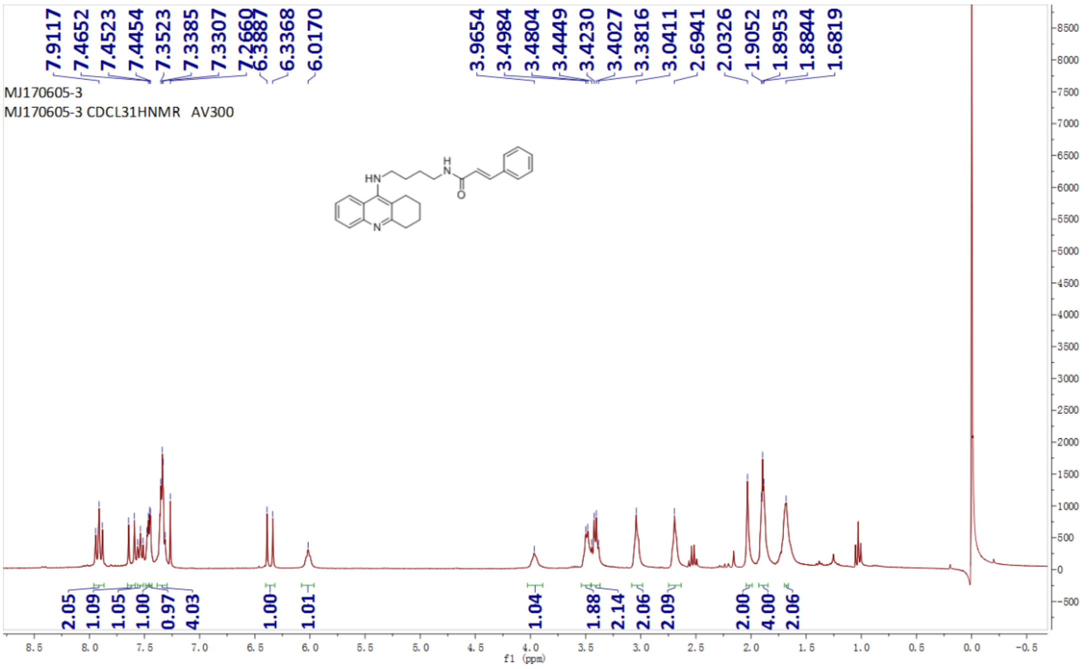

11 ^{13}C-NMR spectrum

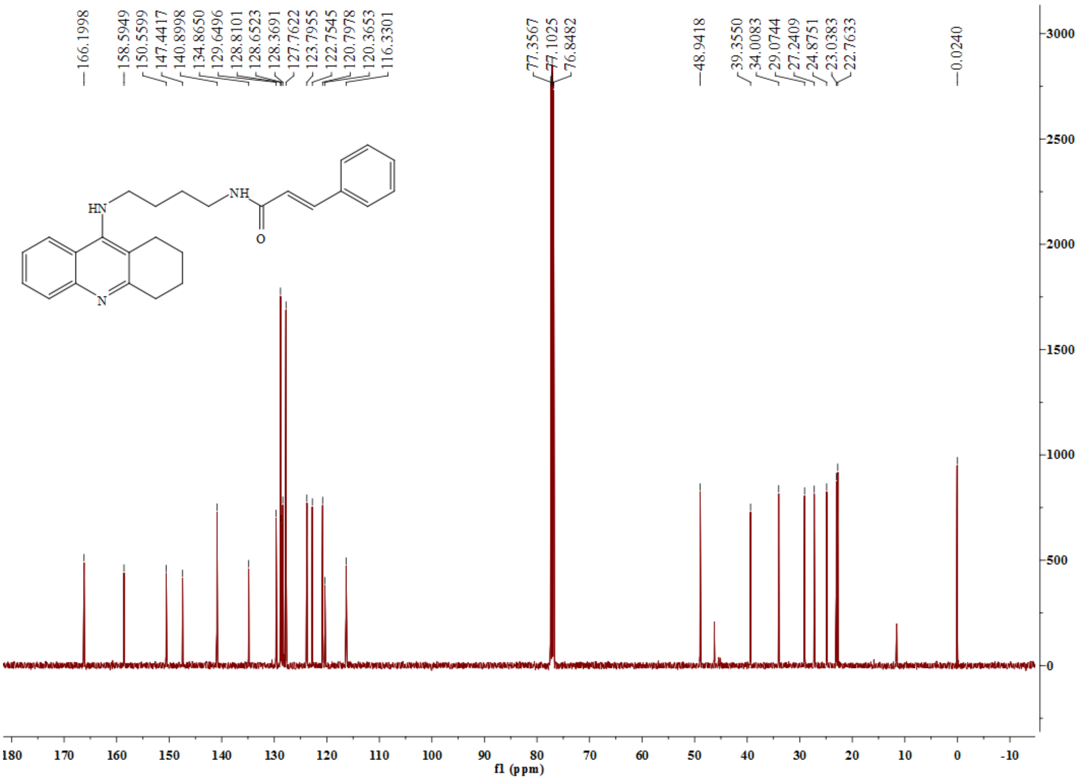

12 ^{1}H-NMR spectrum

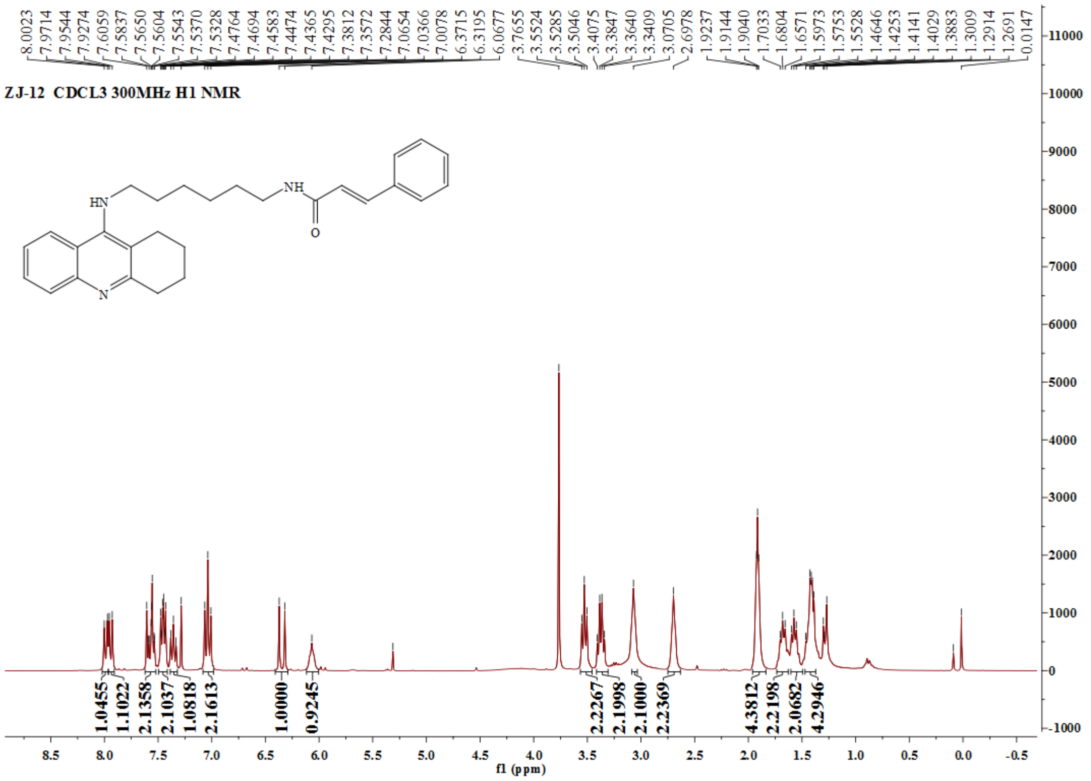

12 ^{13}C-NMR spectrum

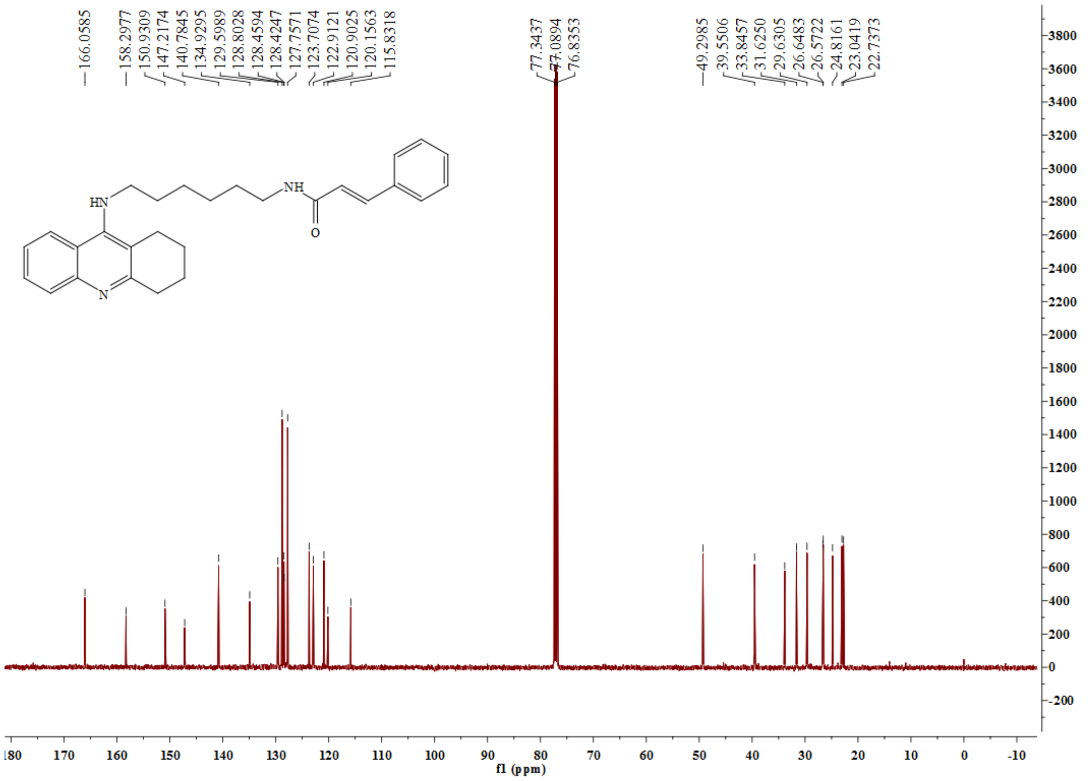

13 ^{1}H-NMR spectrum

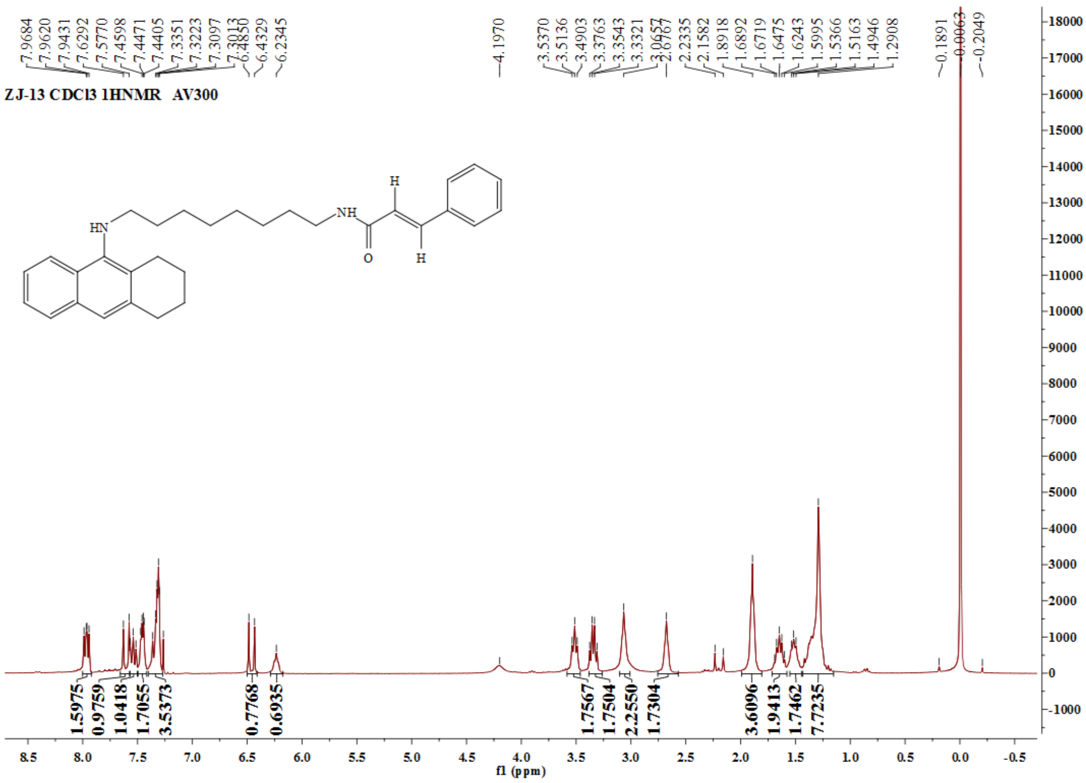

13 ^{13}C-NMR spectrum

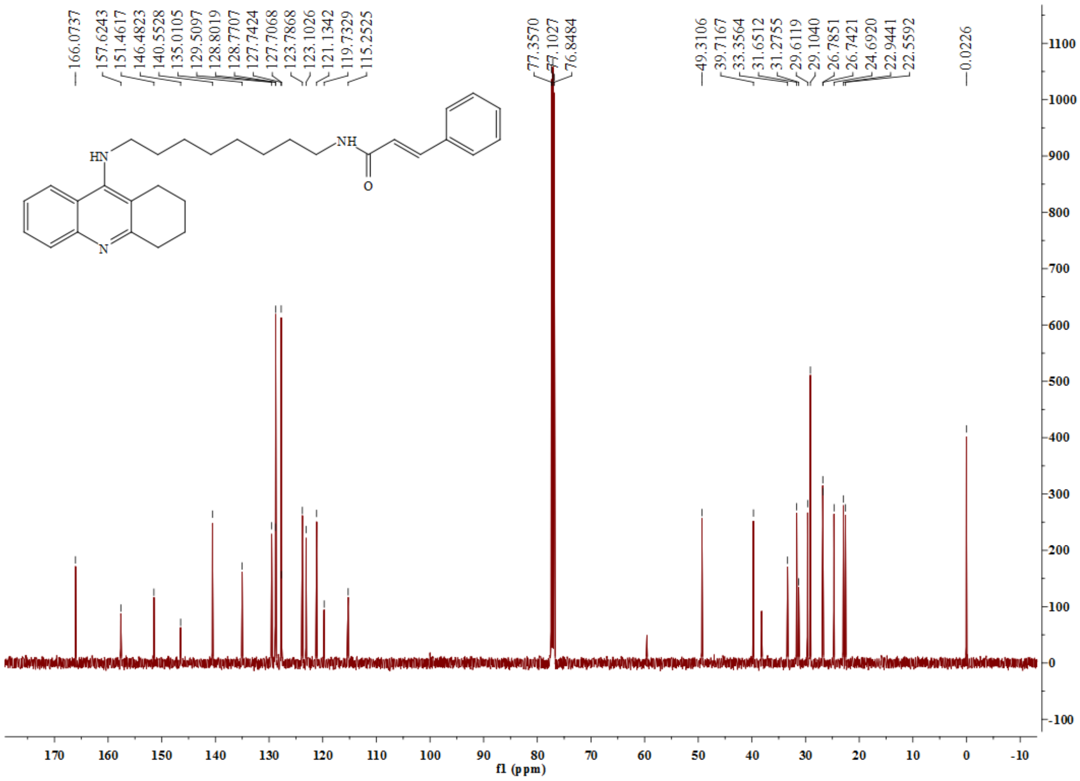

14 ^{1}H-NMR spectrum

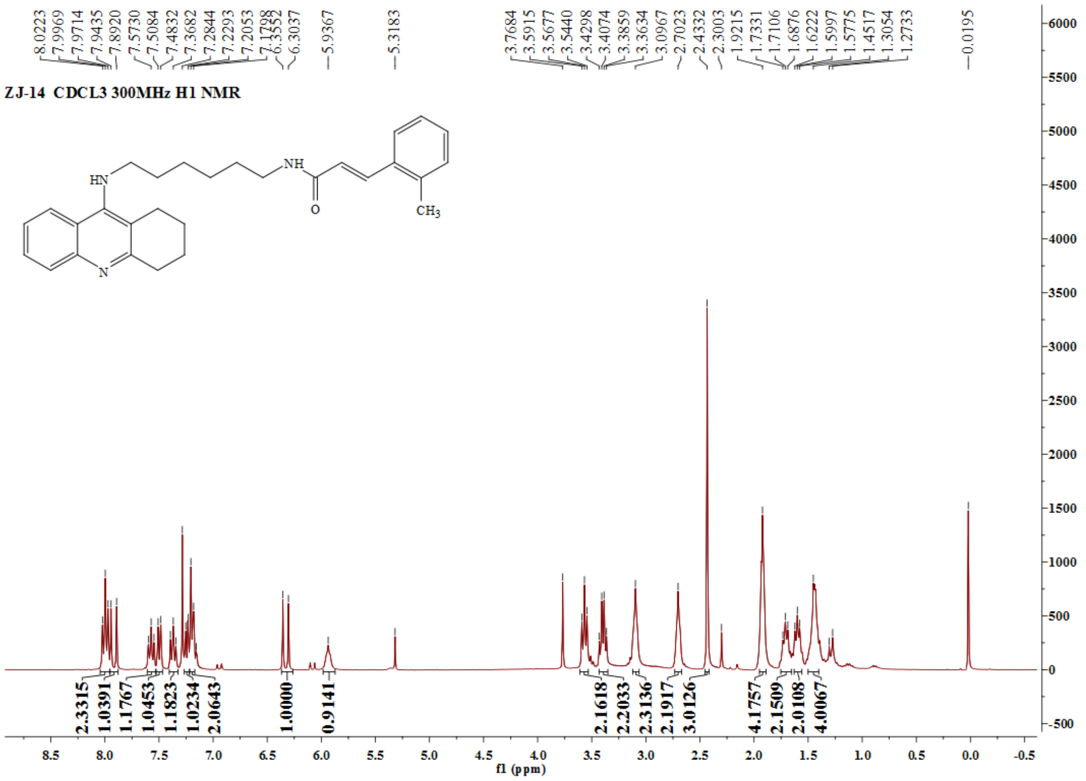

14 ^{13}C-NMR spectrum

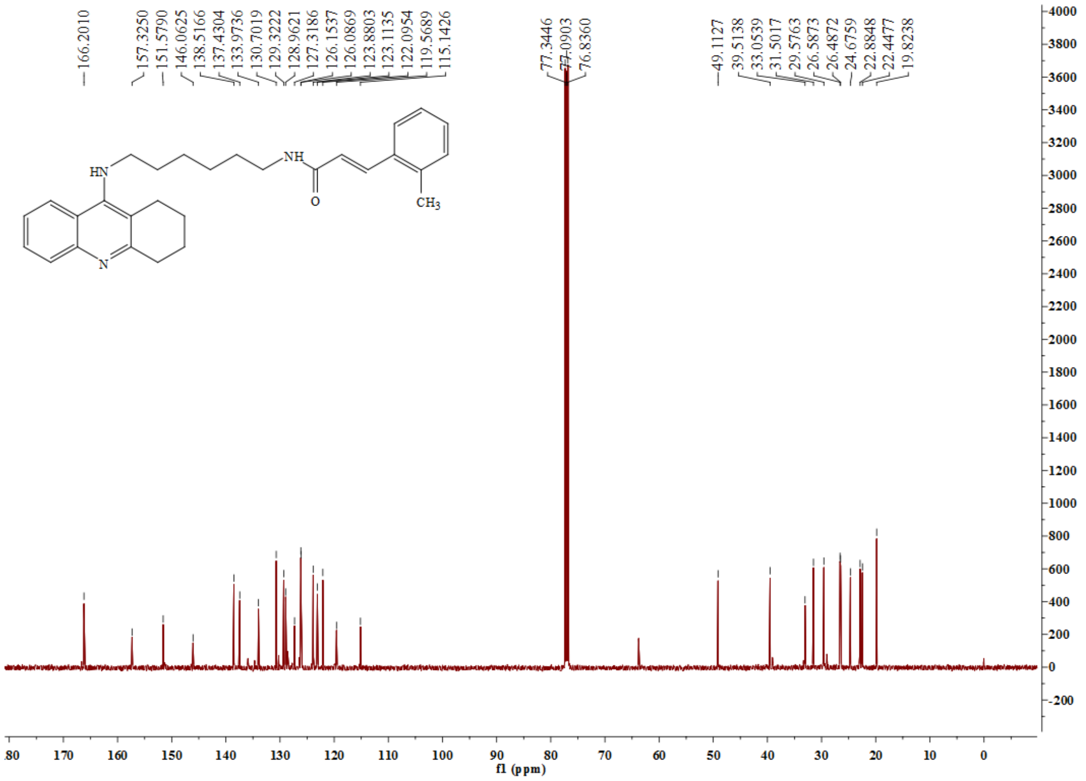

15 ^{1}H-NMR spectrum

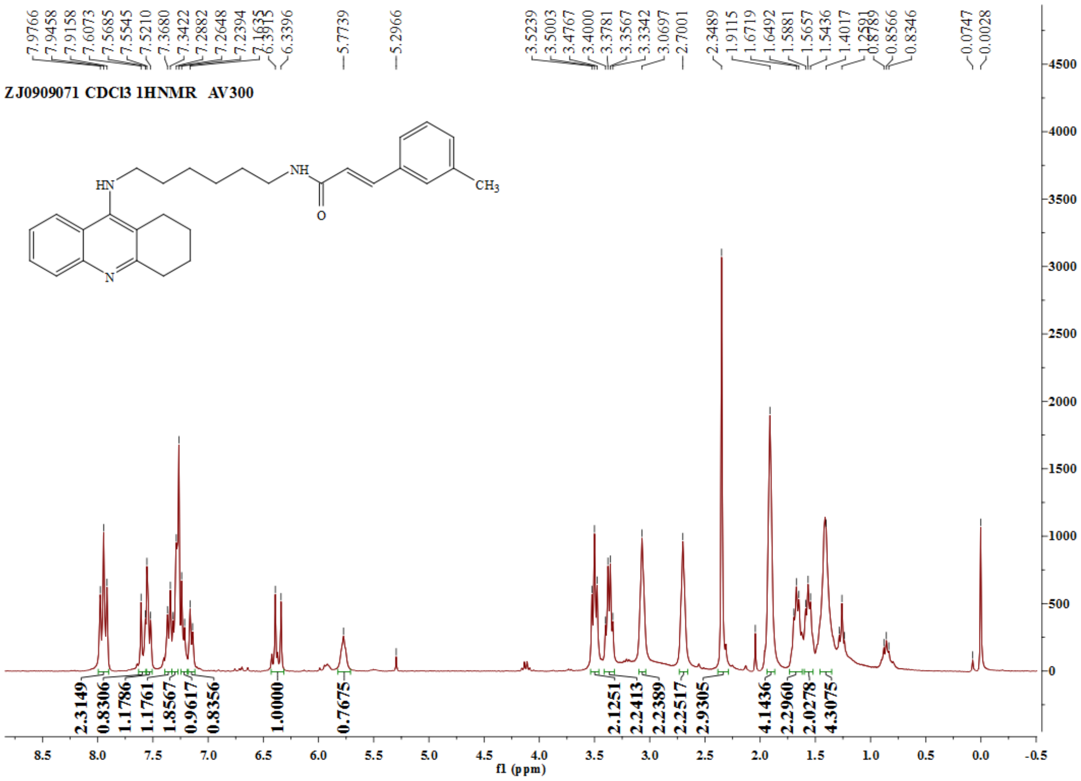

15 ^{13}C-NMR spectrum

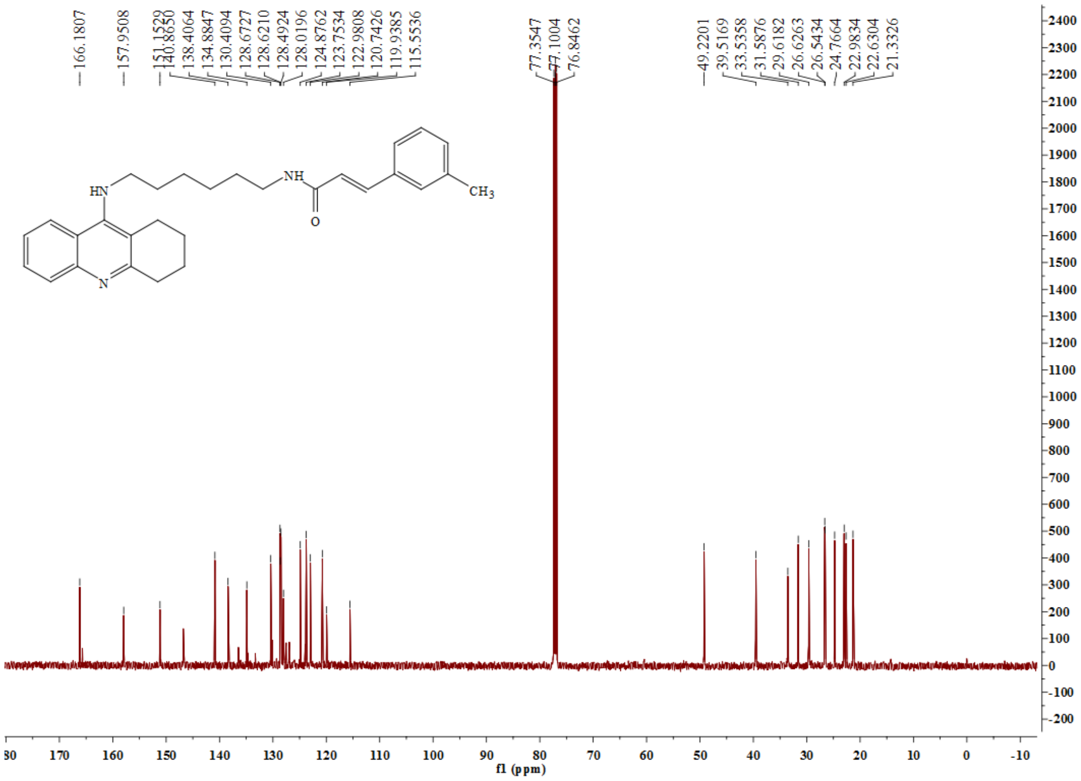

16 ^{1}H-NMR spectrum

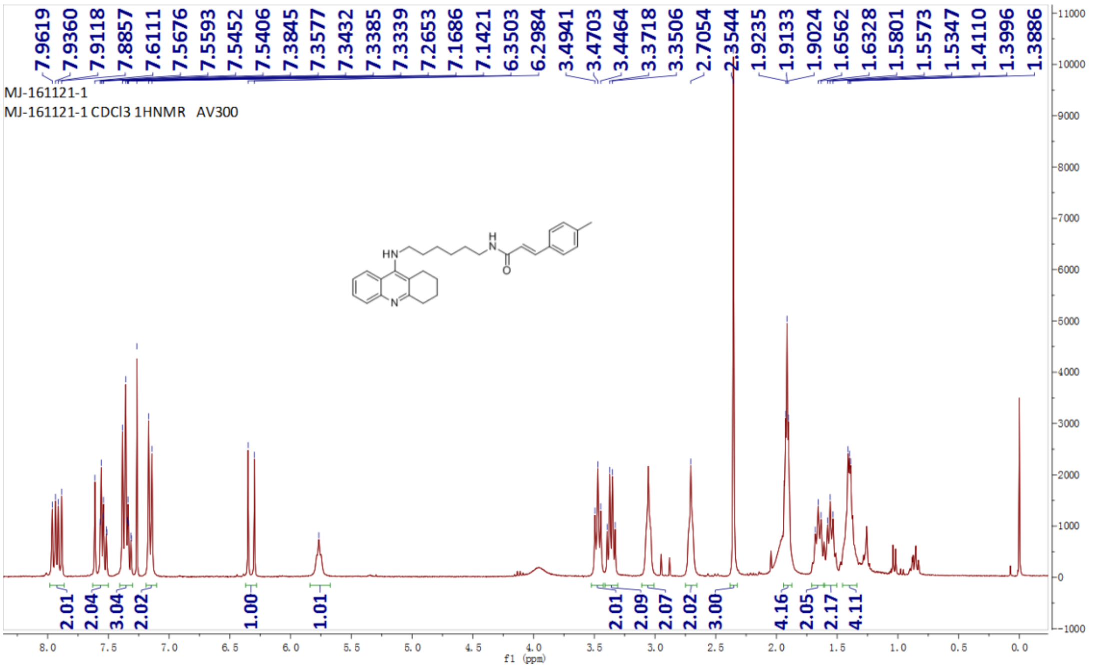

16 ^{13}C-NMR spectrum

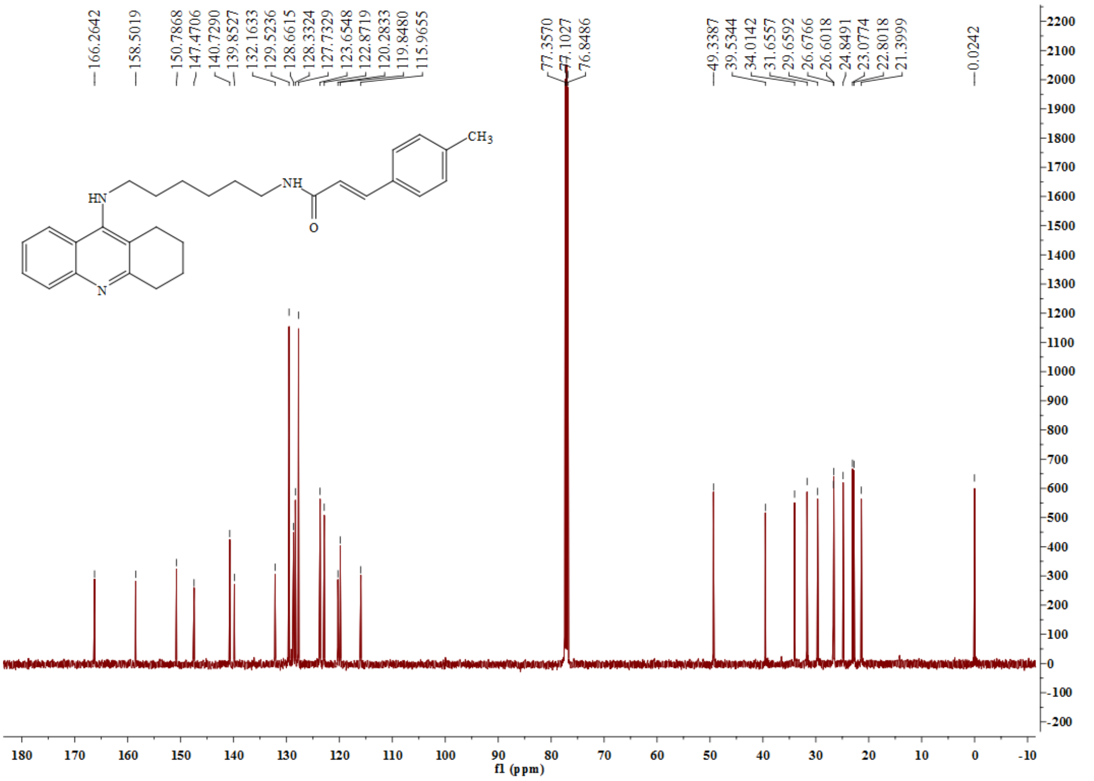

17 ^{1}H-NMR spectrum

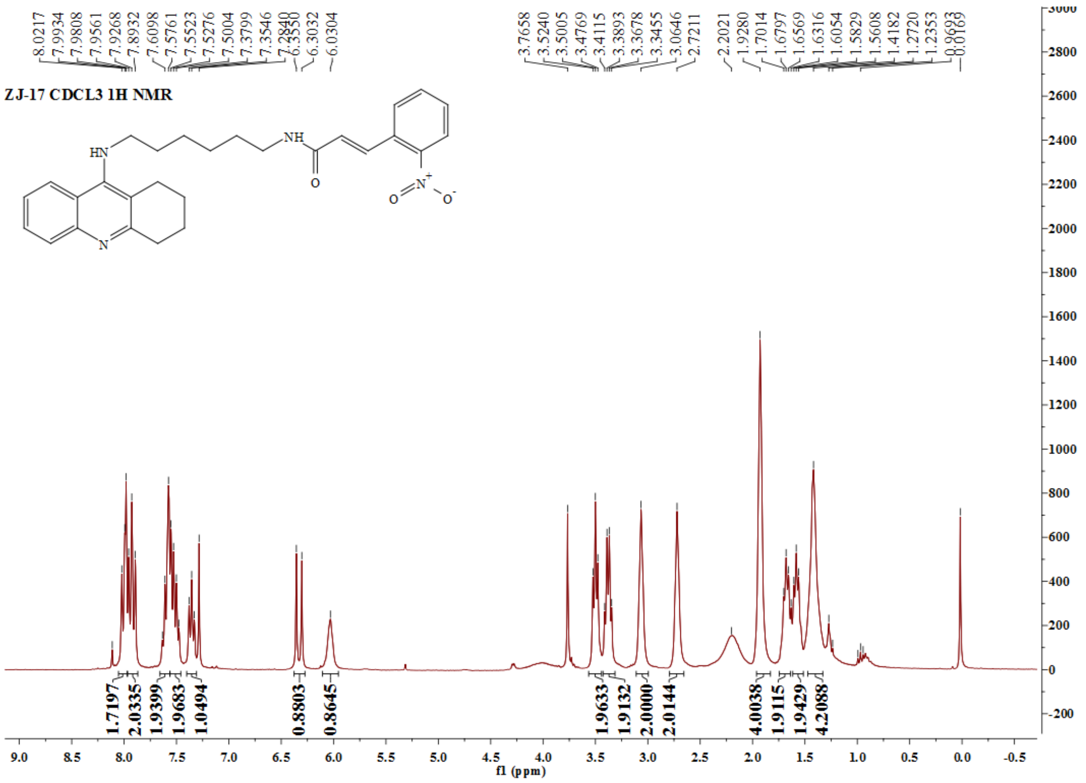

17 ^{13}C-NMR spectrum

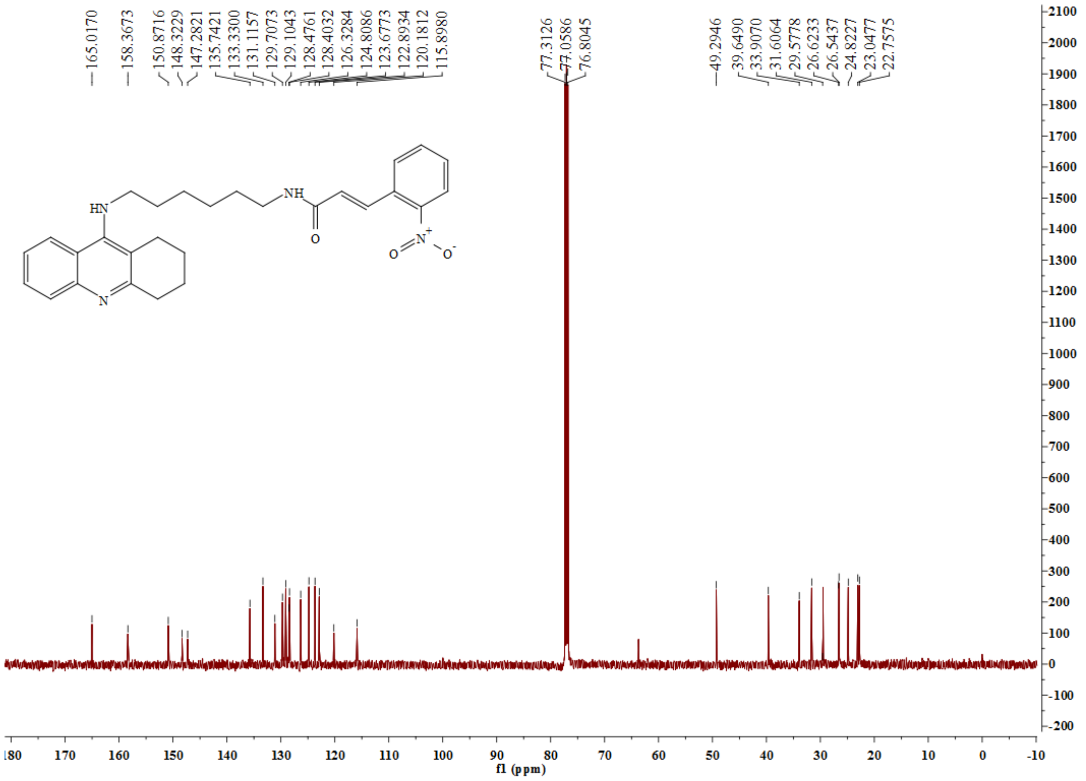

18 ^{1}H-NMR spectrum

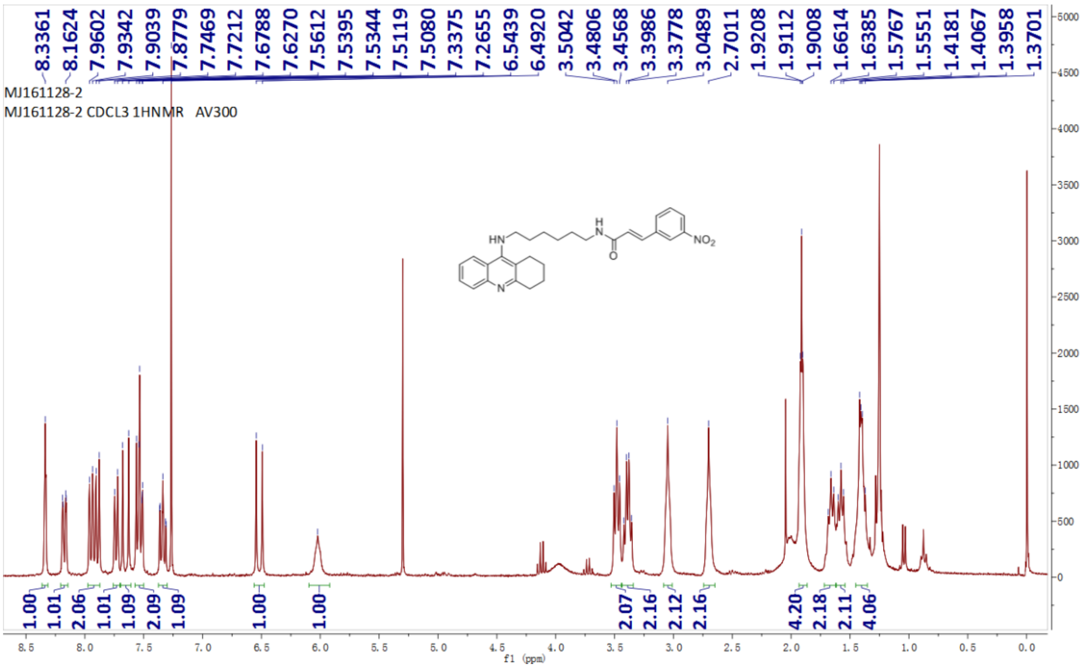

18 ^{13}C-NMR spectrum

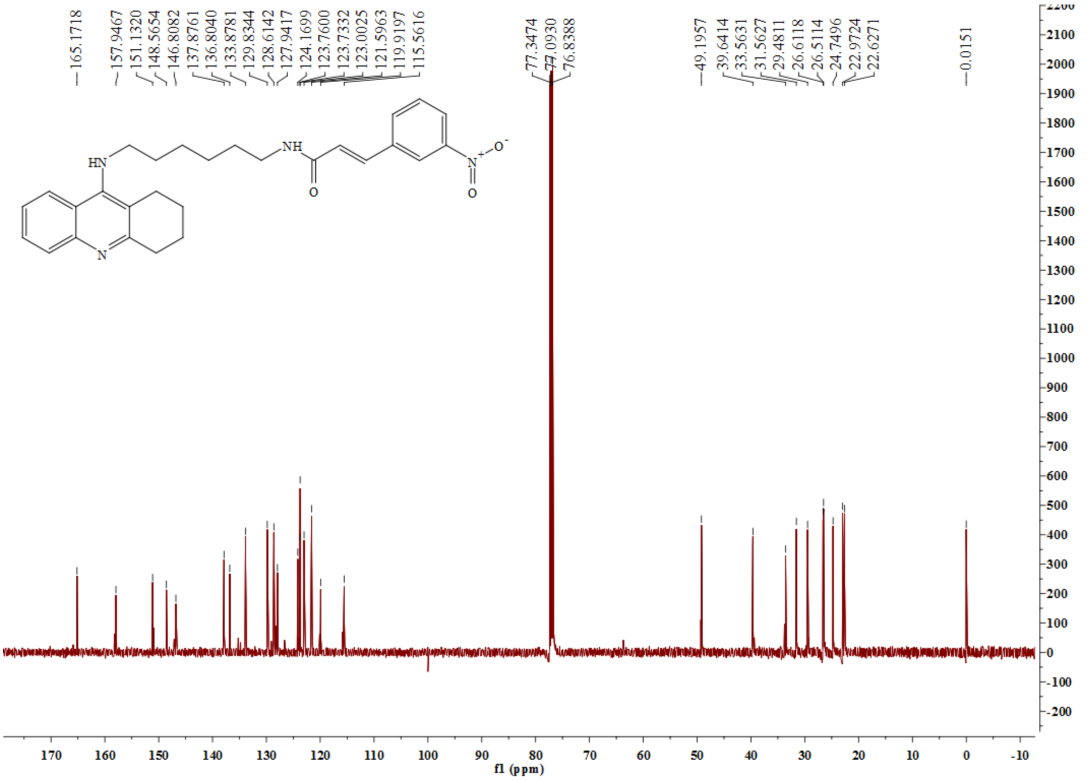

19 ^{1}H-NMR spectrum

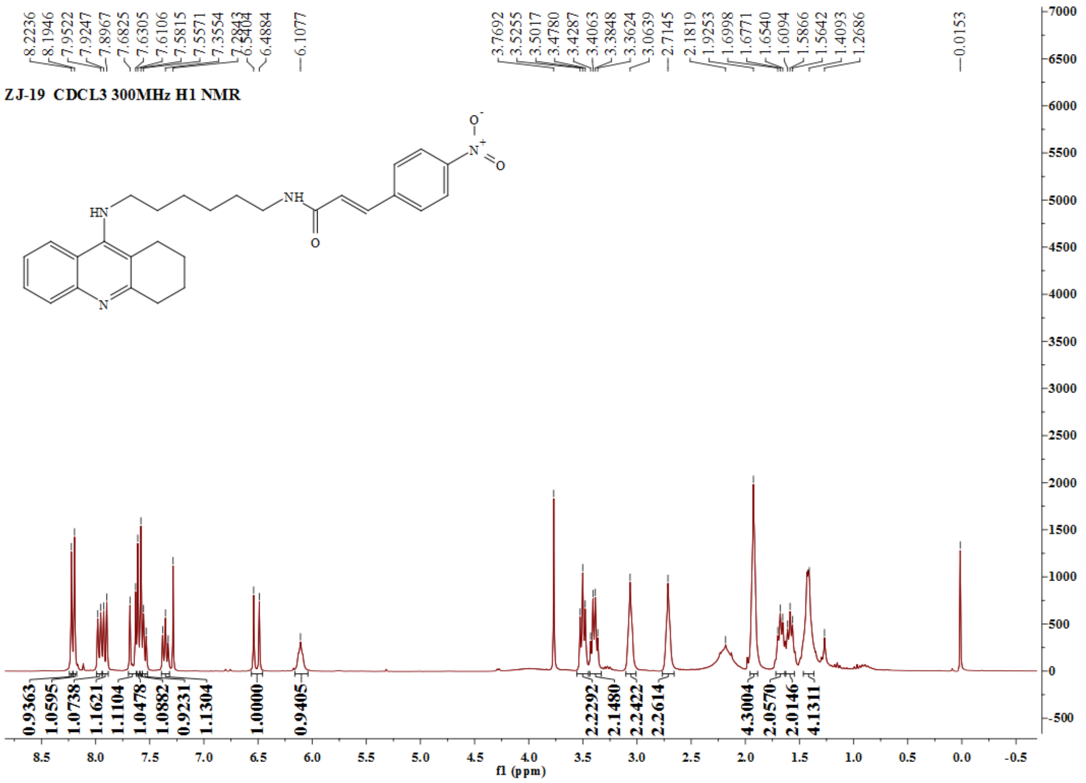

19 ^{13}C-NMR spectrum

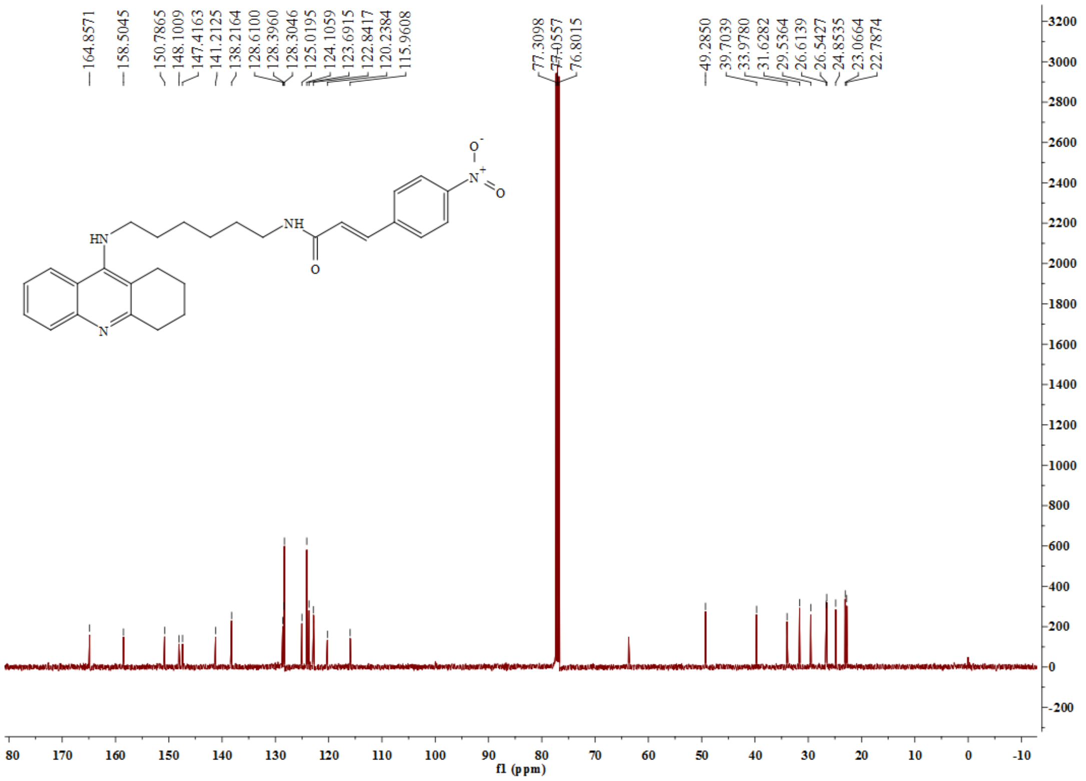

20 ^{1}H-NMR spectrum

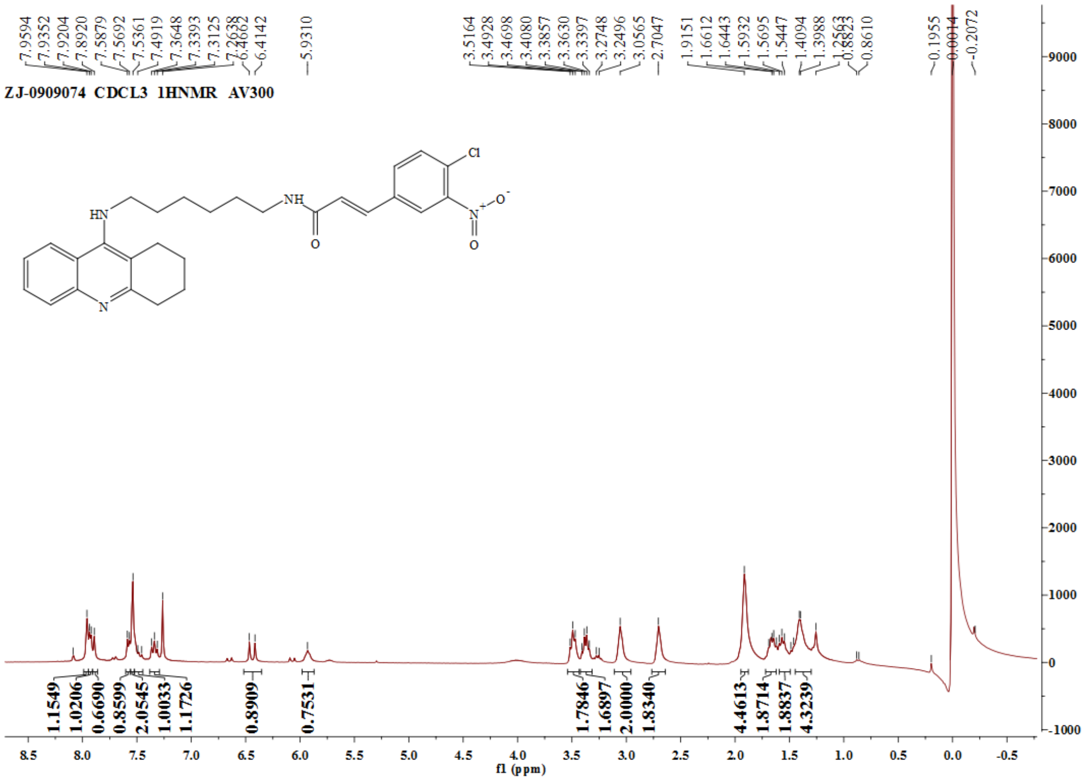

20 ^{13}C-NMR spectrum

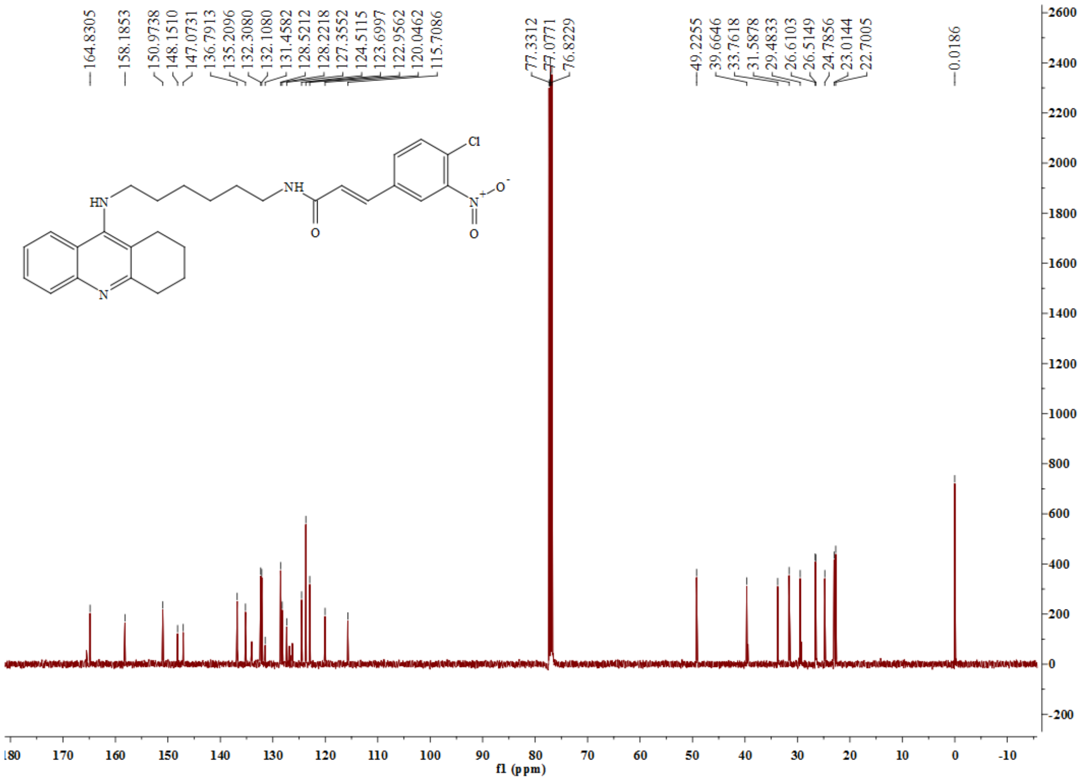

21 ^{1}H-NMR spectrum

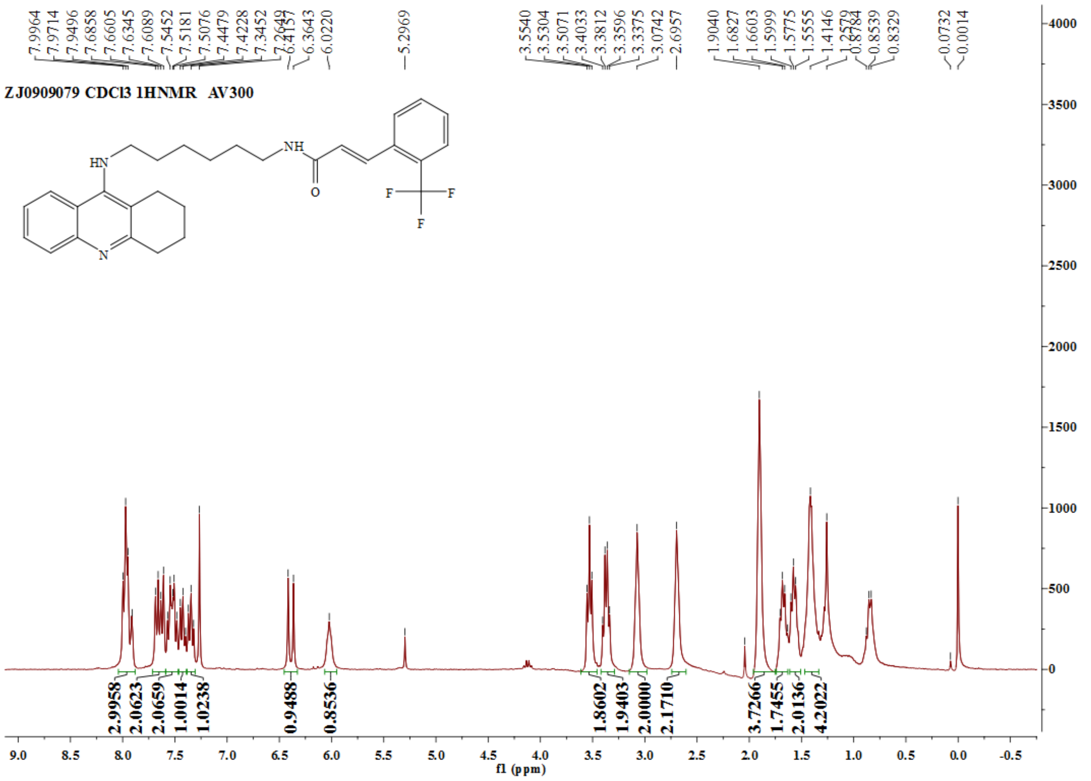

21 ^{13}C-NMR spectrum

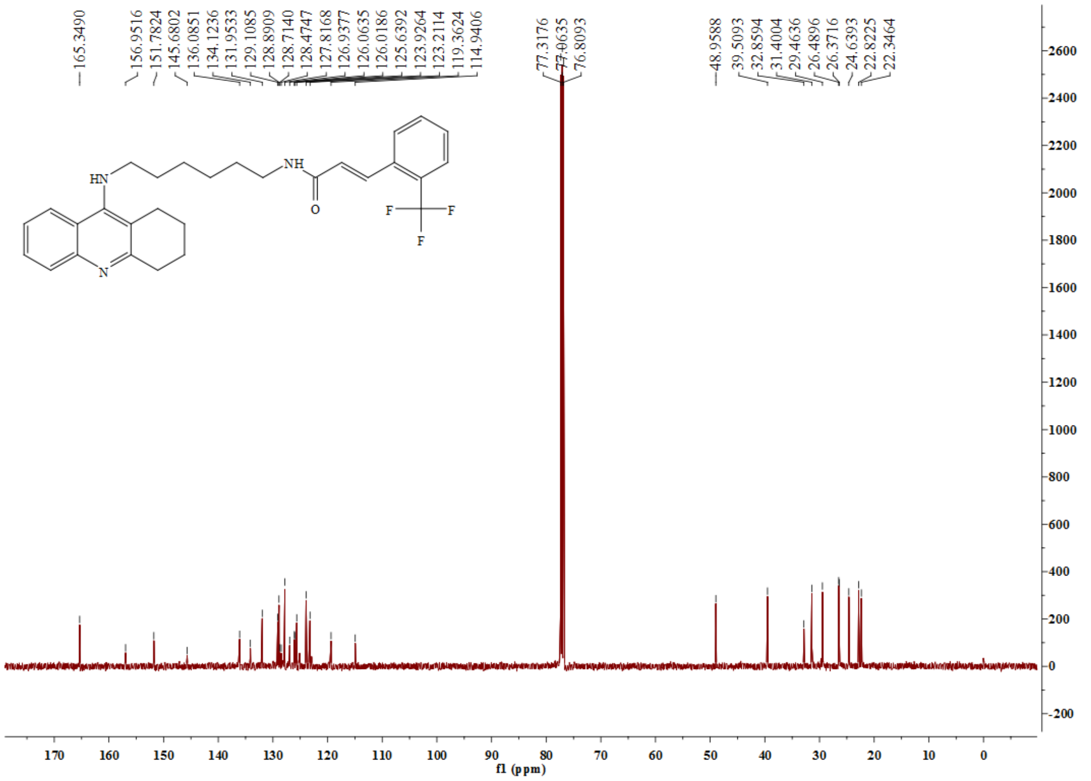

22 ^{1}H-NMR spectrum

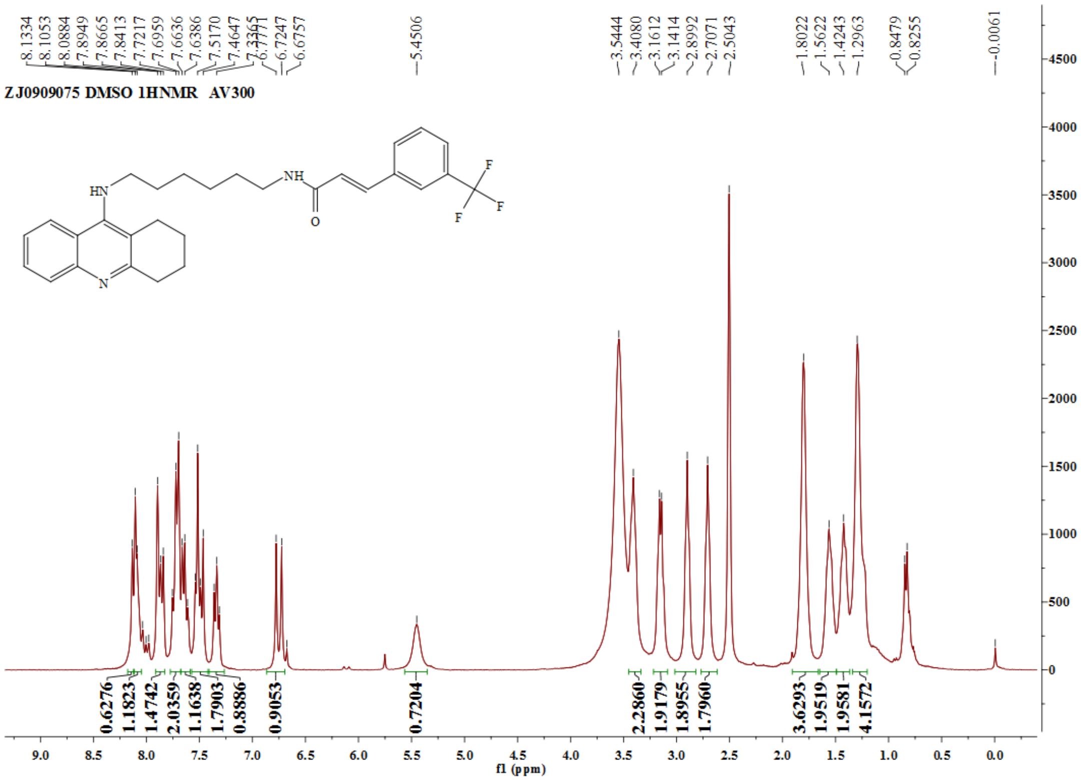

22 ^{13}C-NMR spectrum

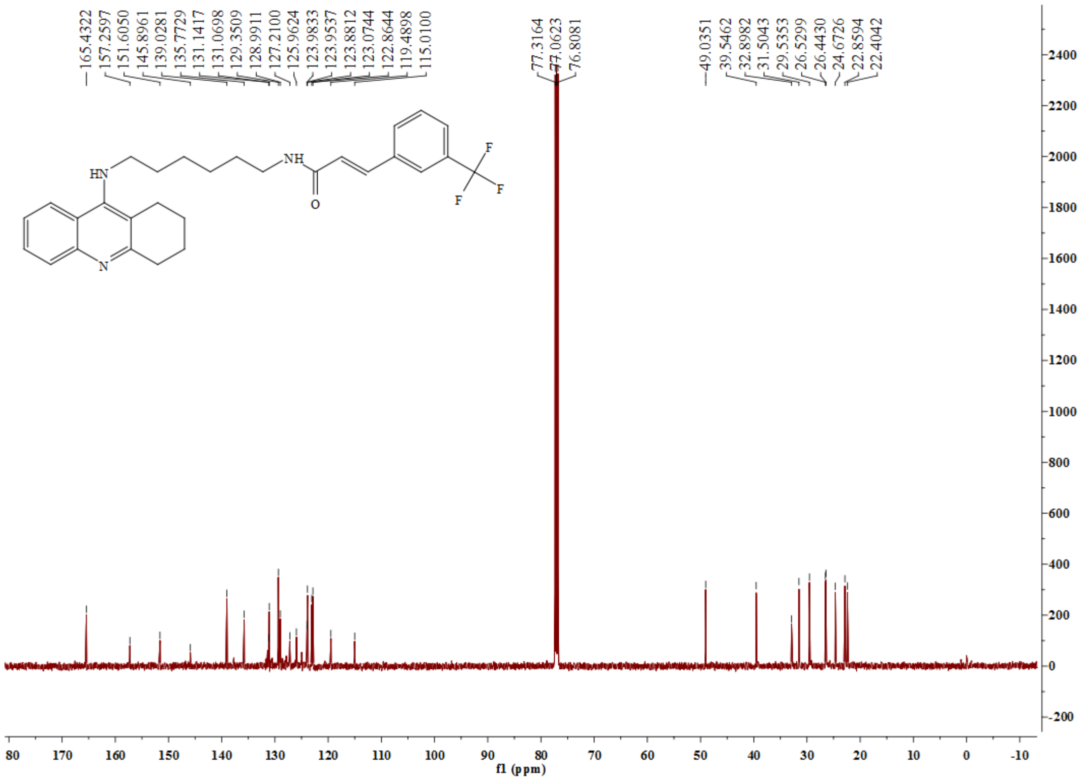

23 ^{1}H-NMR spectrum

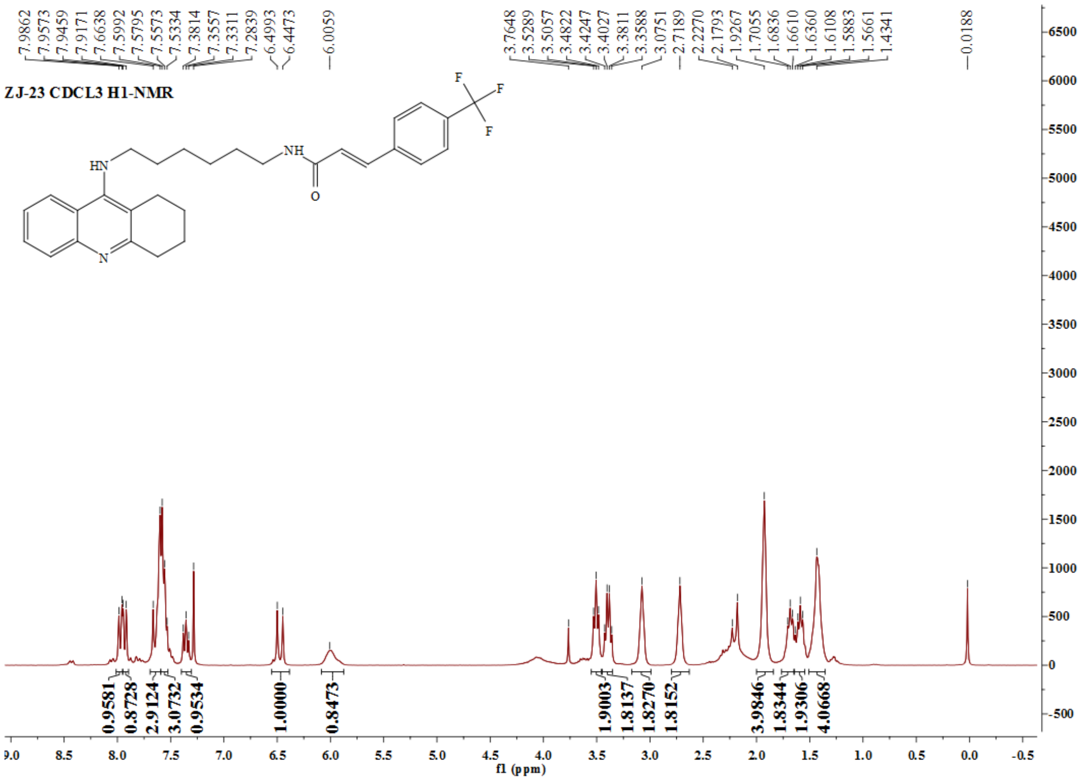

23 ^{13}C-NMR spectrum

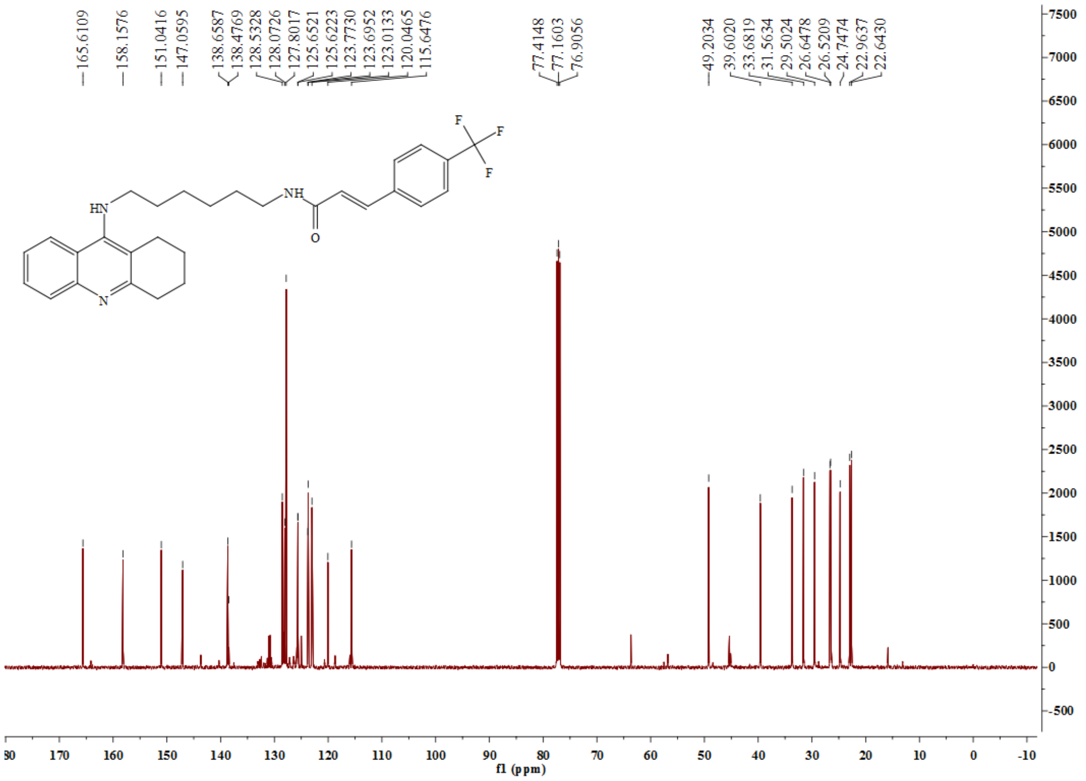

24 ^{1}H-NMR spectrum

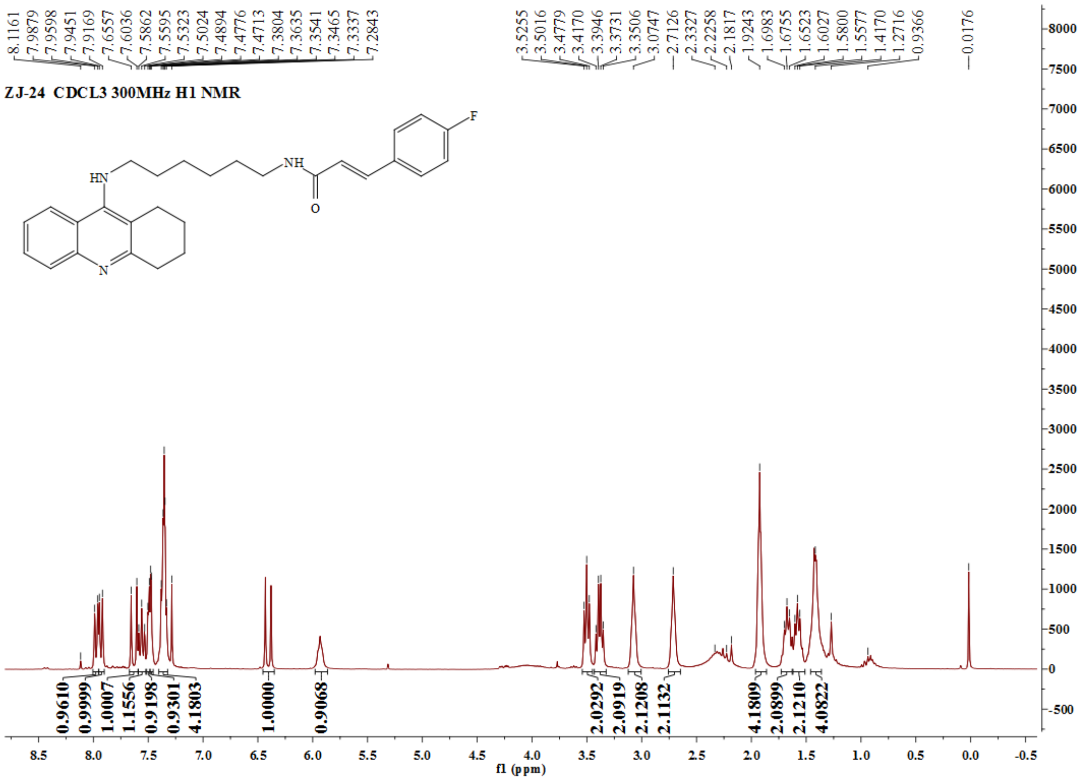

24 ^{13}C-NMR spectrum

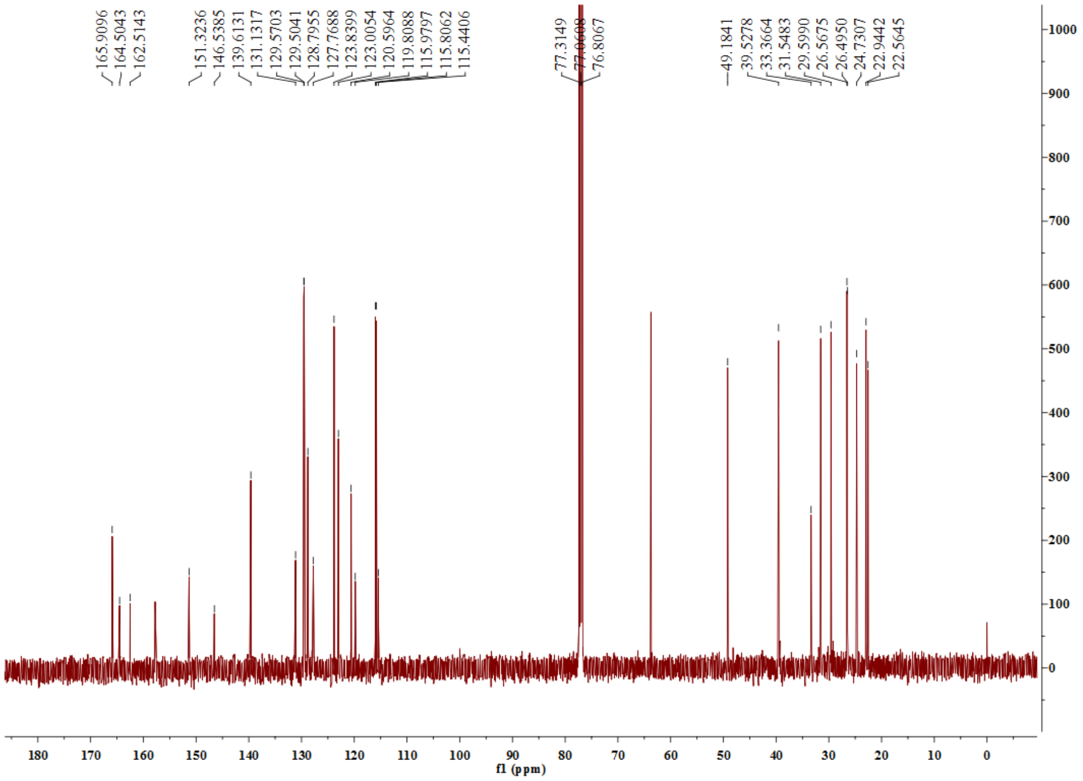

25 ^{1}H-NMR spectrum

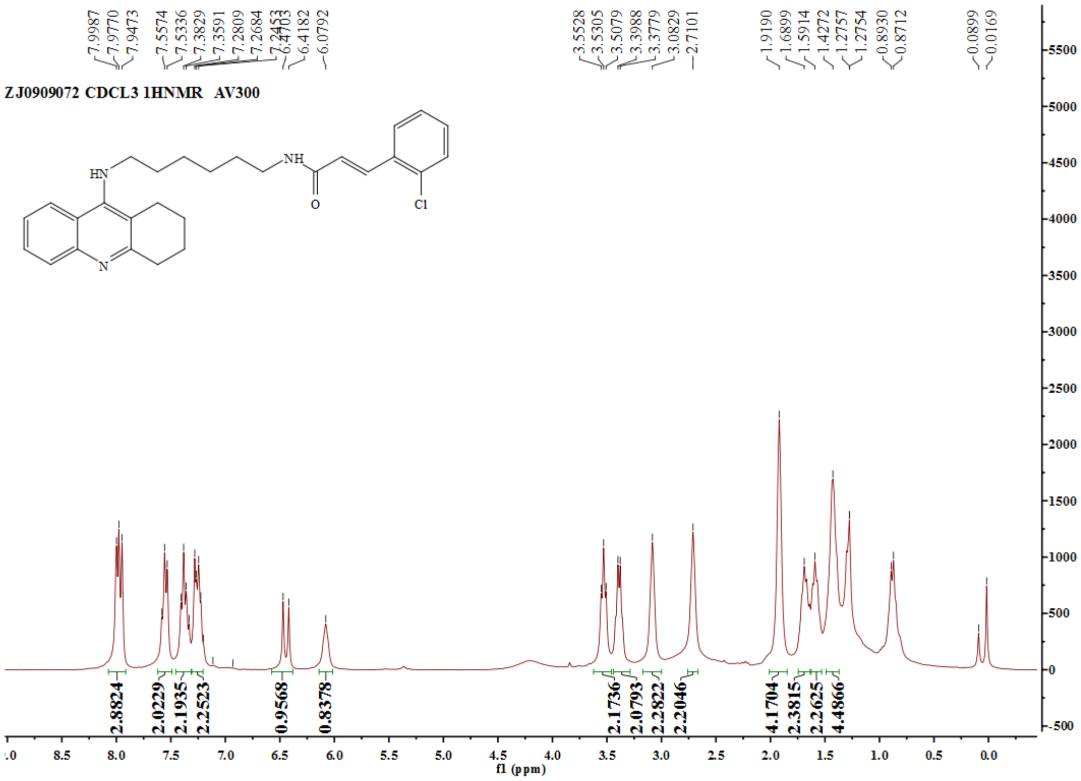

25 ^{13}C-NMR spectrum

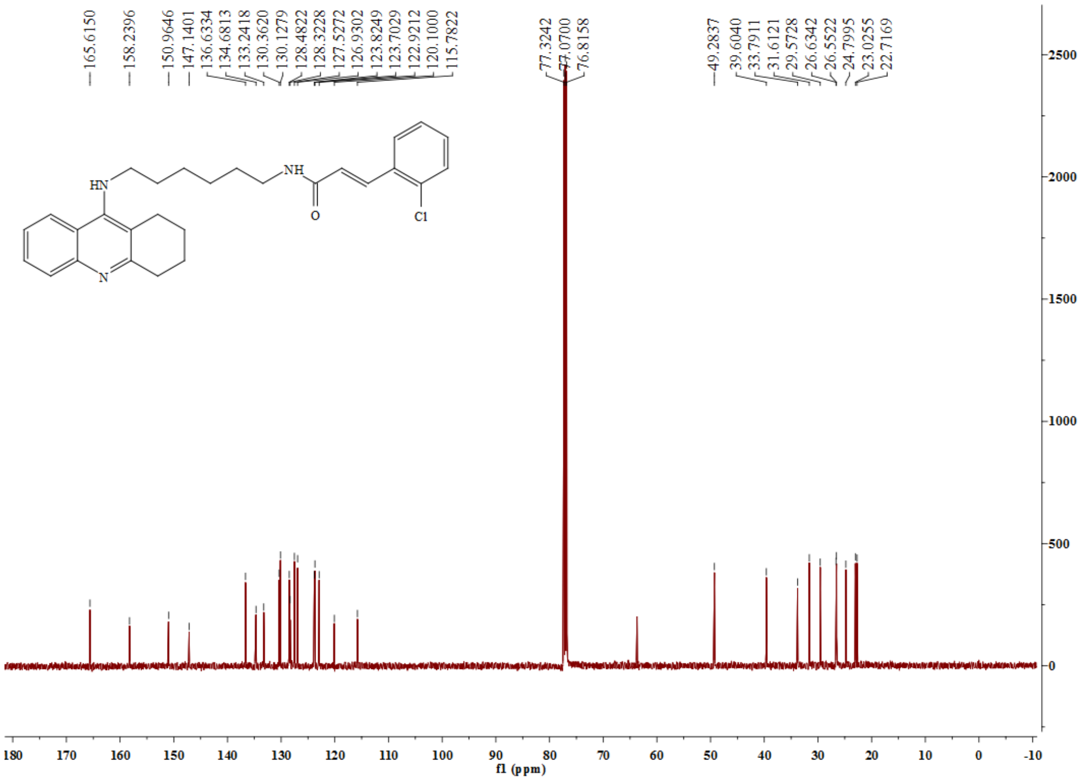

26 ^{1}H-NMR spectrum

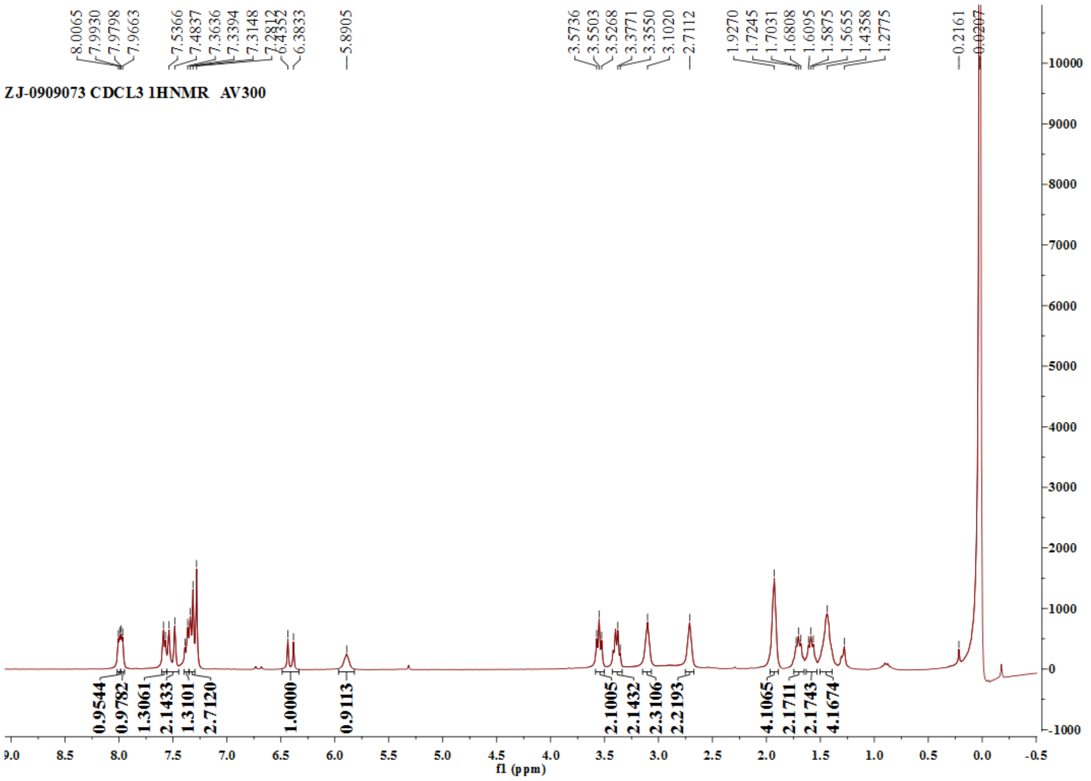

26 ^{13}C-NMR spectrum

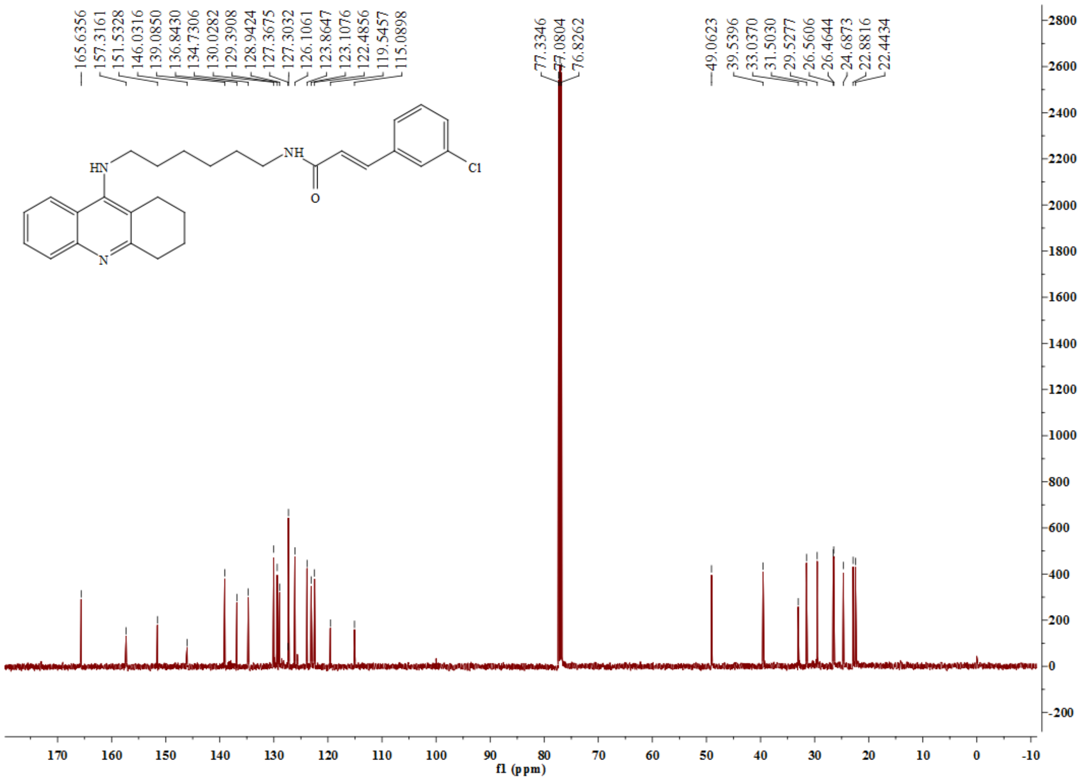

27 ^{1}H-NMR spectrum

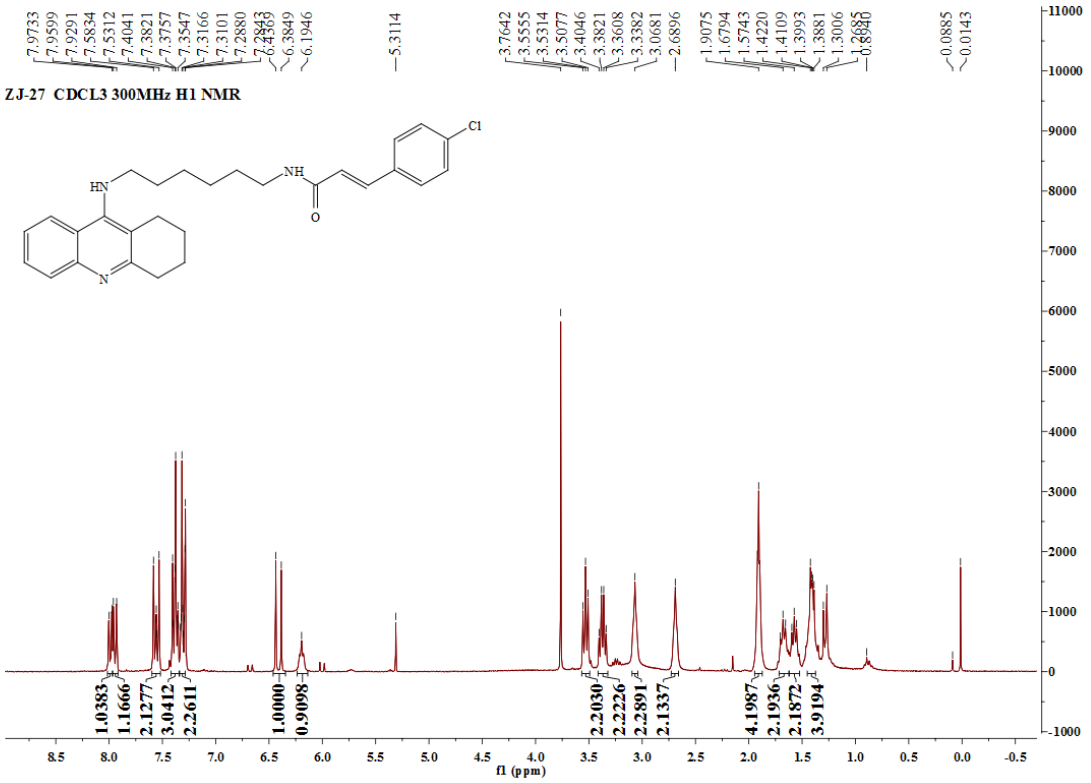

27 ^{13}C-NMR spectrum

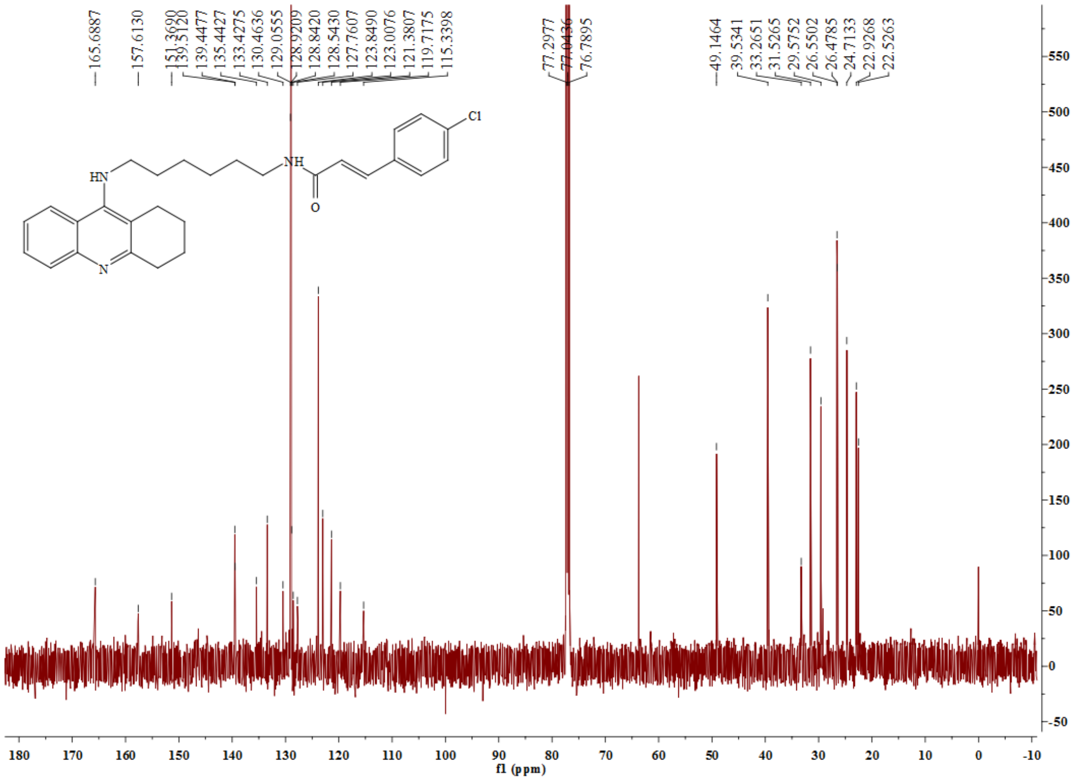

28 ^{1}H-NMR spectrum

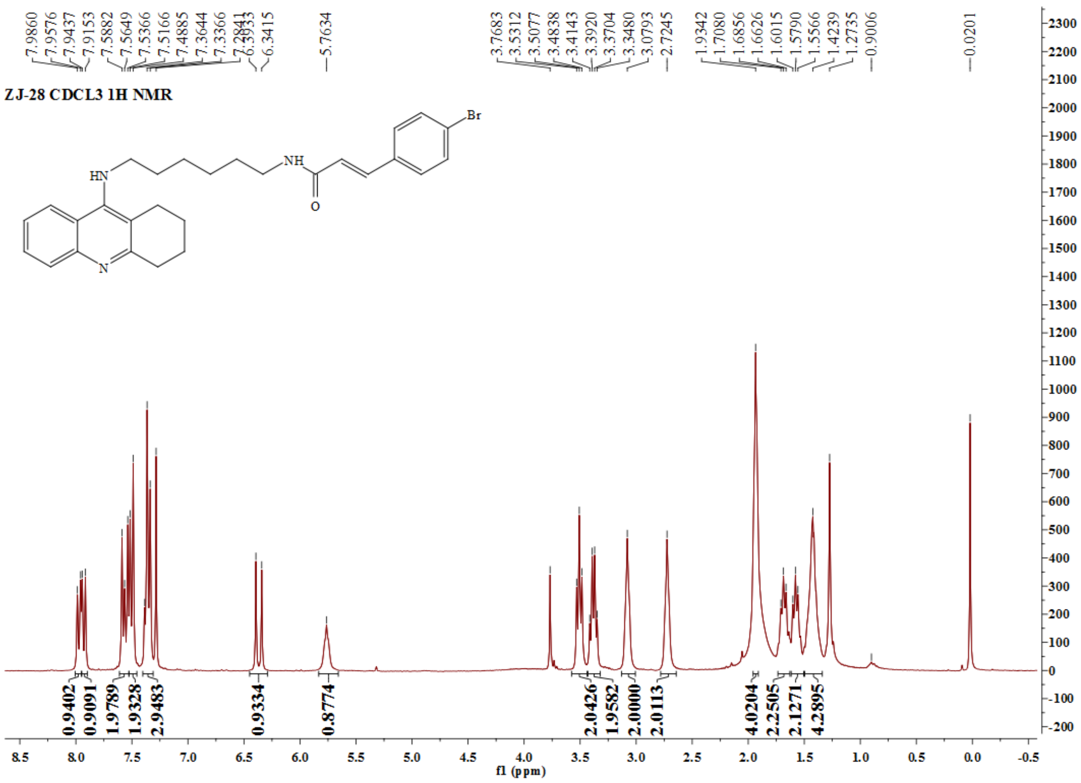

28 ^{13}C-NMR spectrum

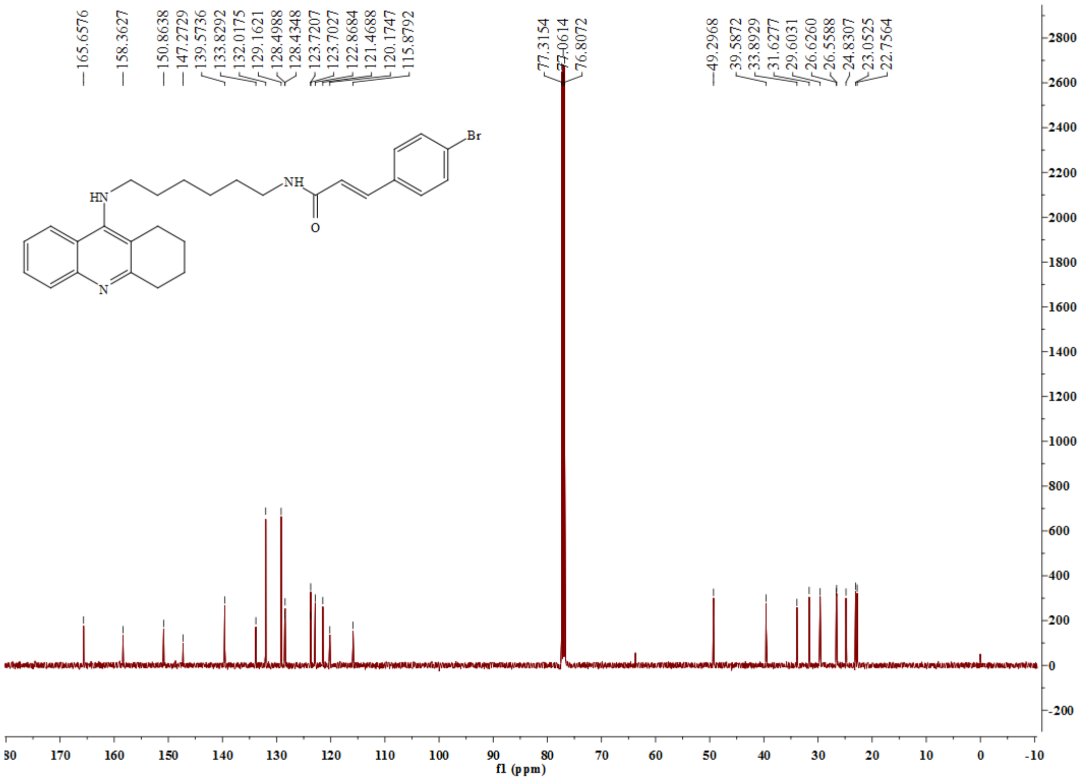

29 ¹H-NMR spectrum

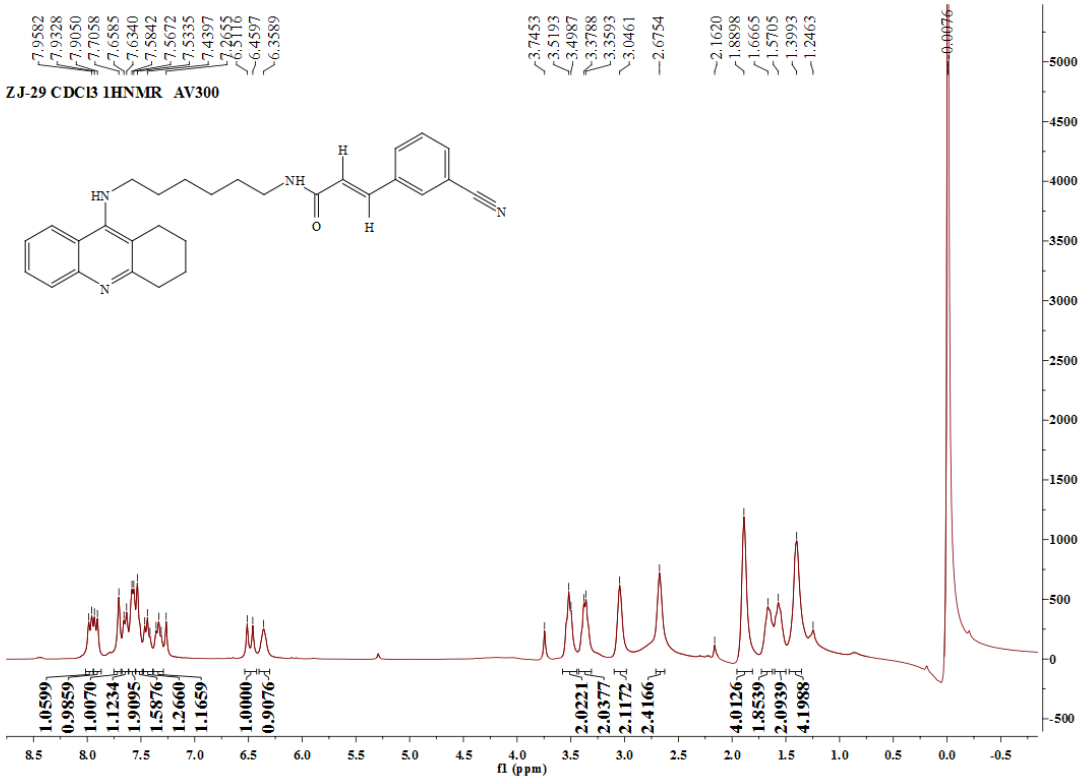

29 ¹³C-NMR spectrum

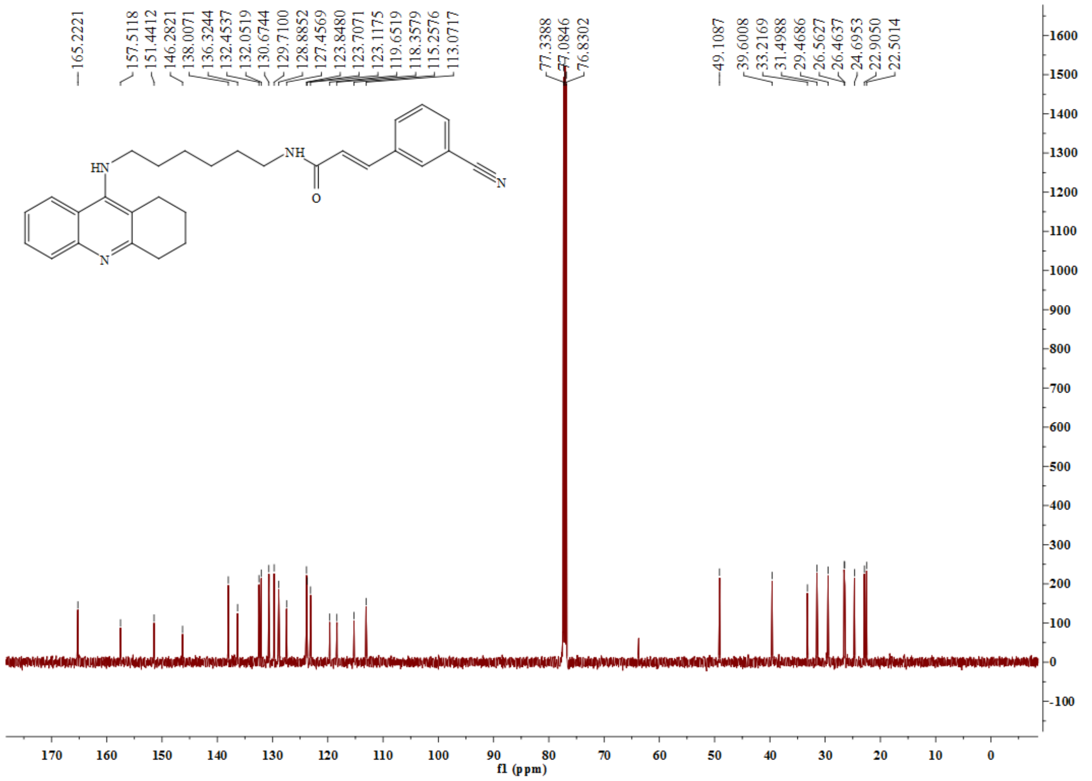

30 ^{1}H-NMR spectrum

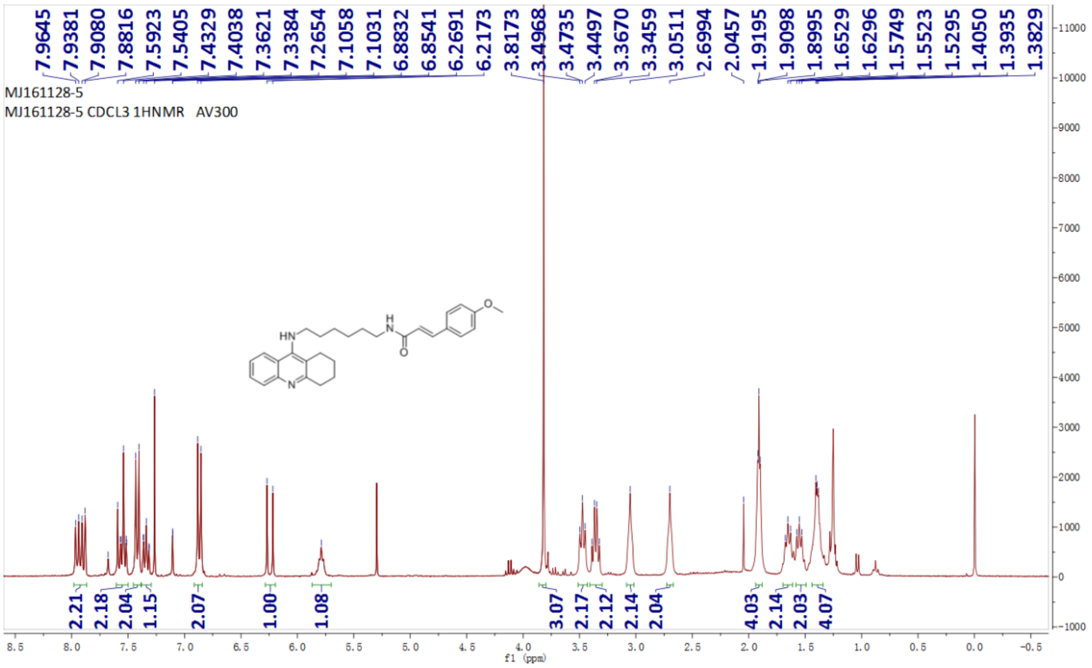

30 ^{13}C-NMR spectrum

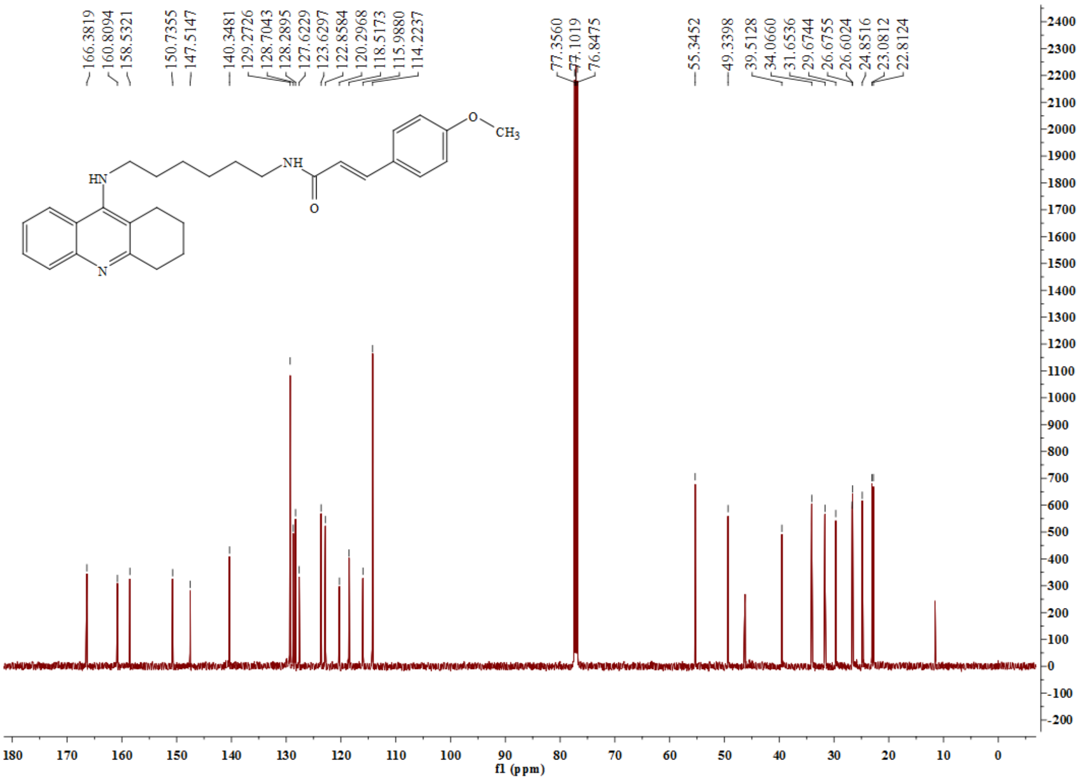

31 ^{1}H-NMR spectrum

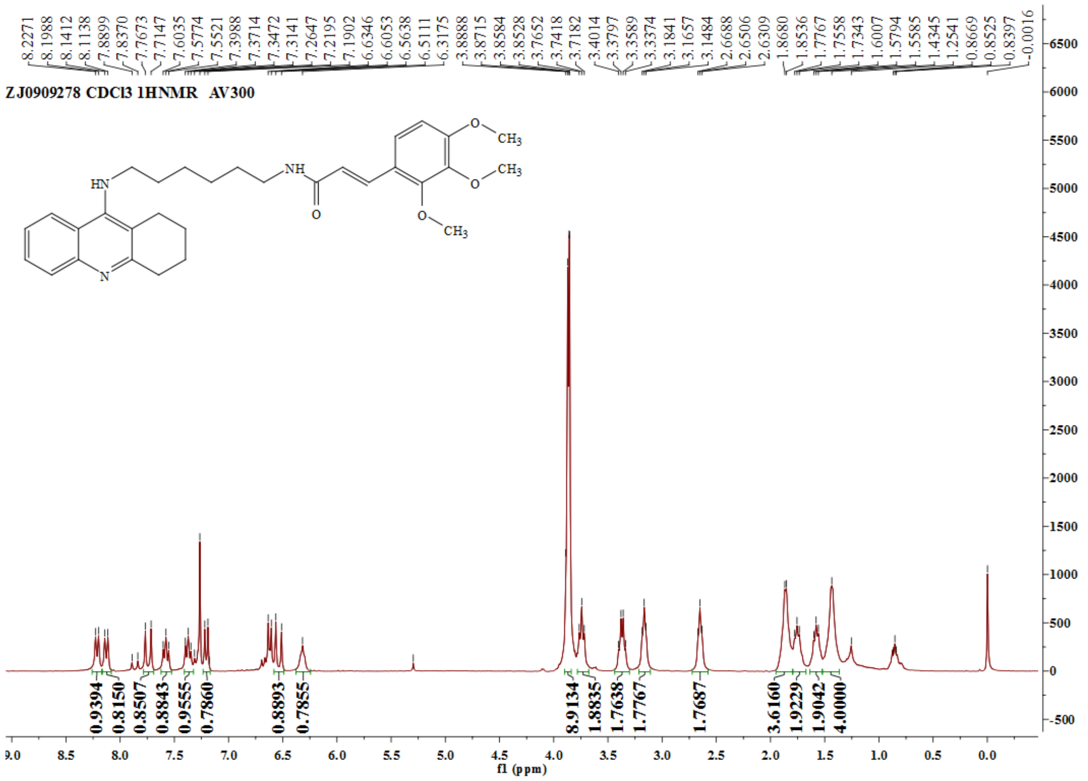

31 ^{13}C-NMR spectrum

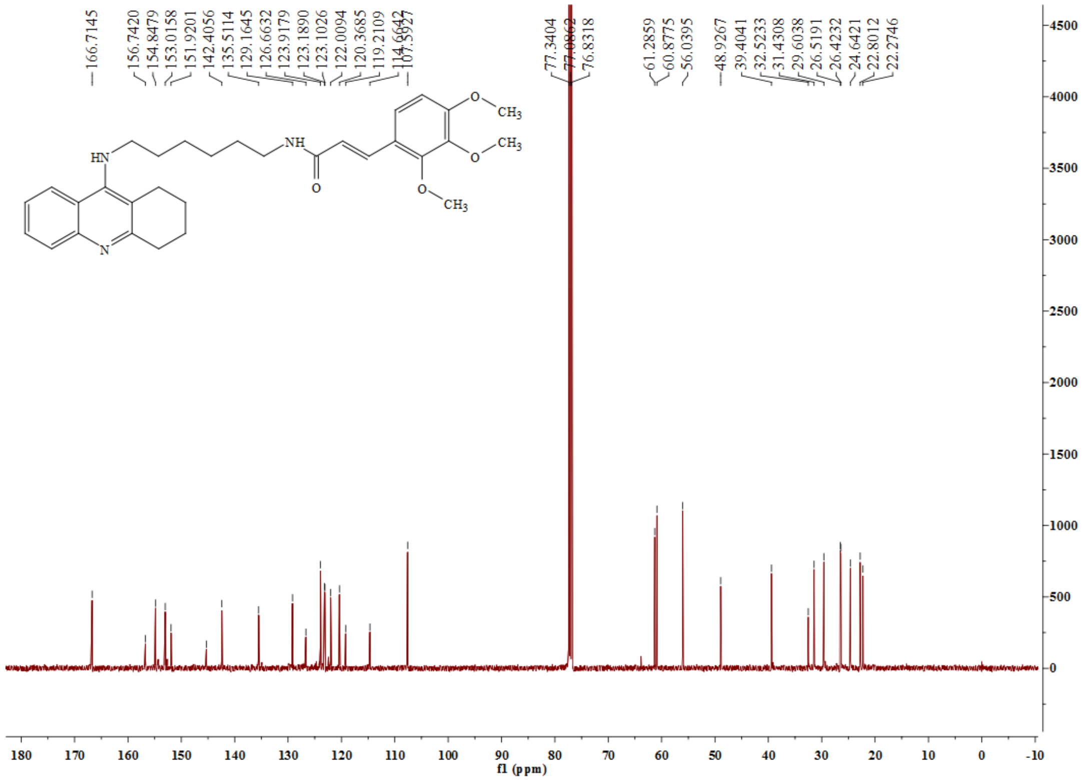

32 ^{1}H-NMR spectrum

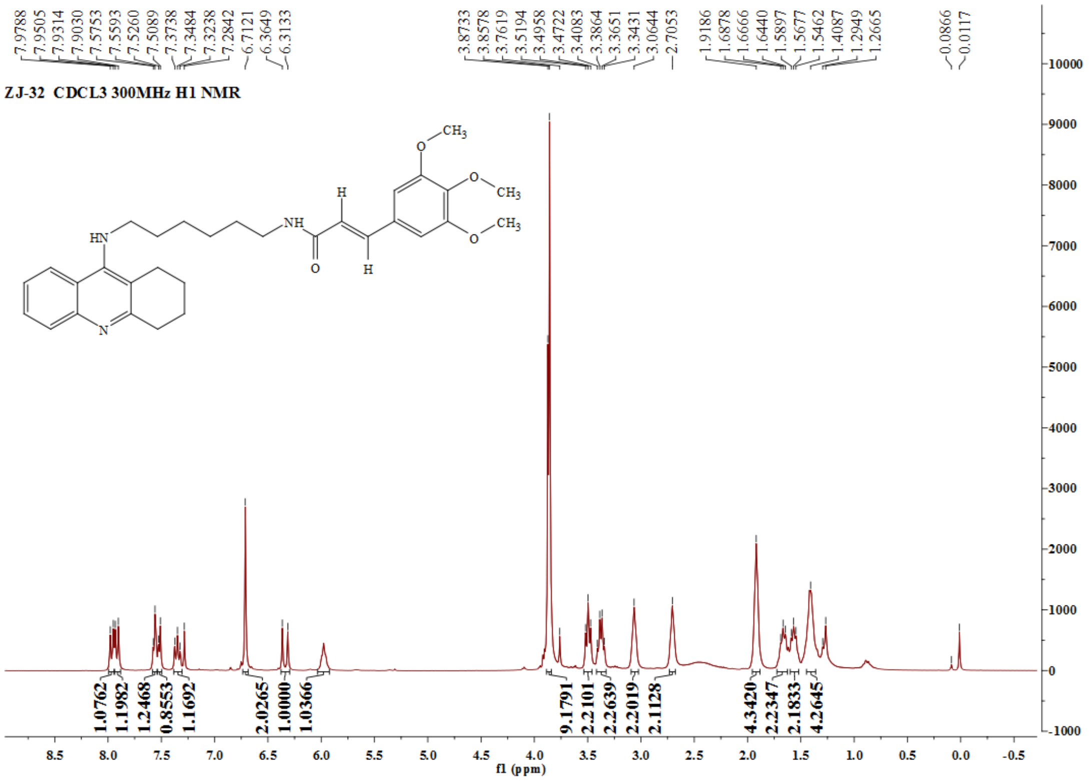

32 ^{13}C-NMR spectrum

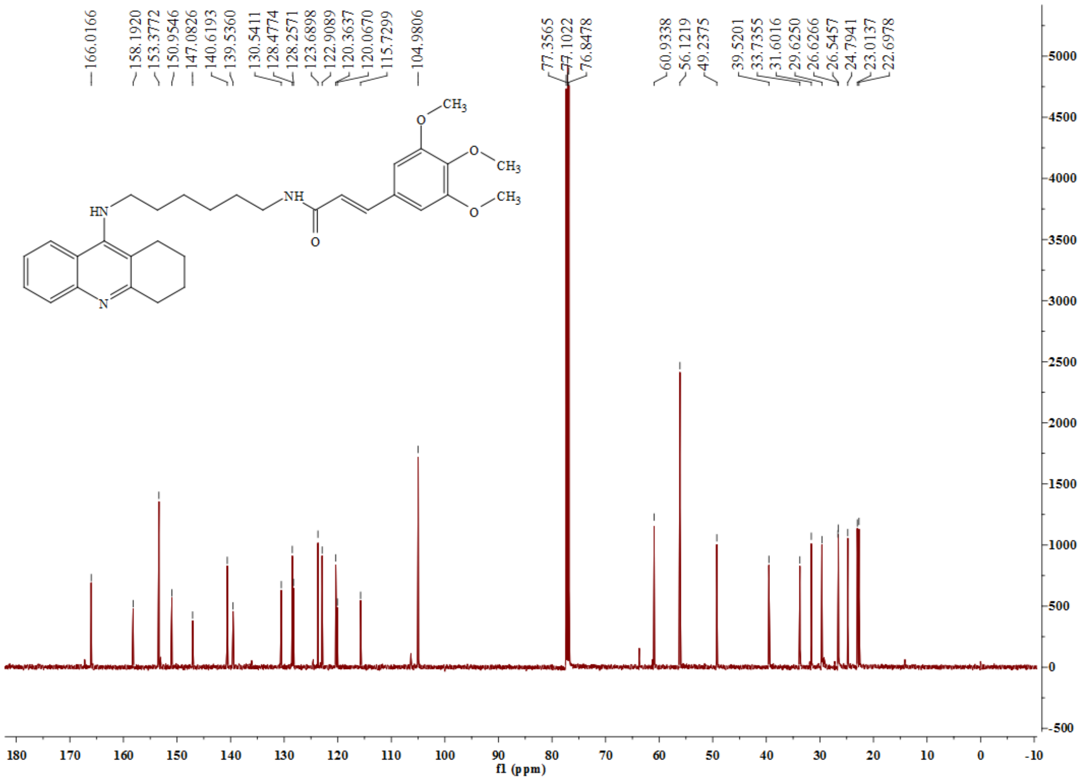

33 ^{1}H-NMR spectrum

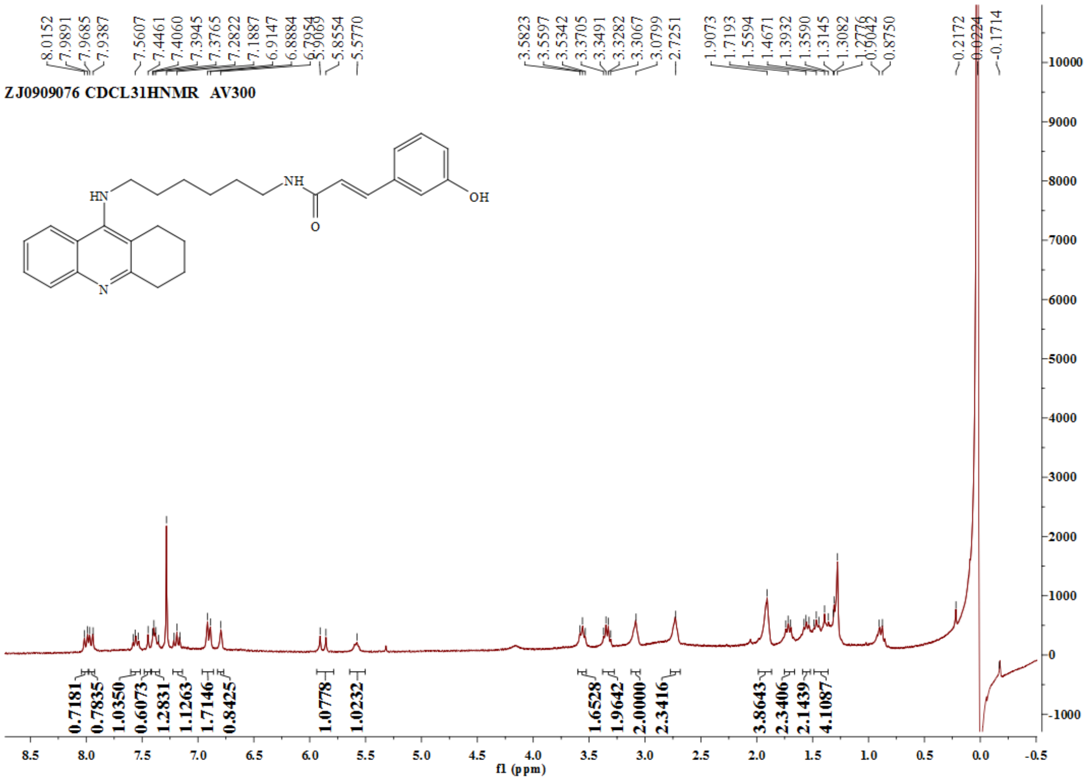

33 ^{13}C-NMR spectrum

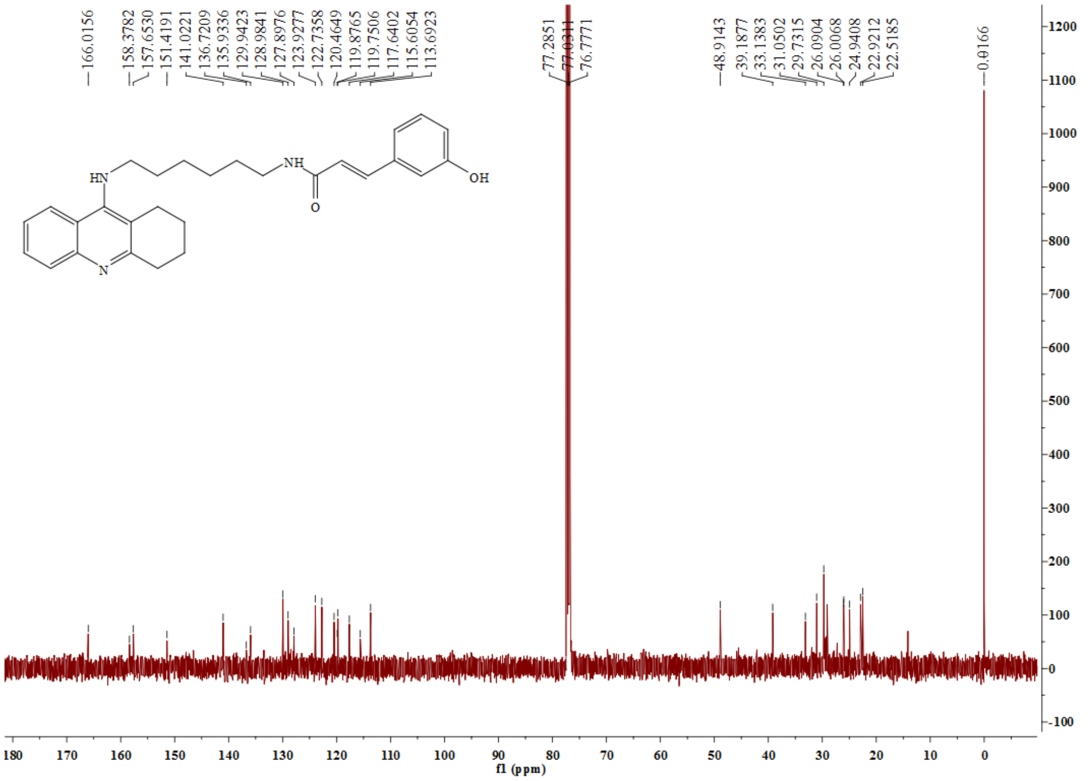

34 ^{1}H-NMR spectrum

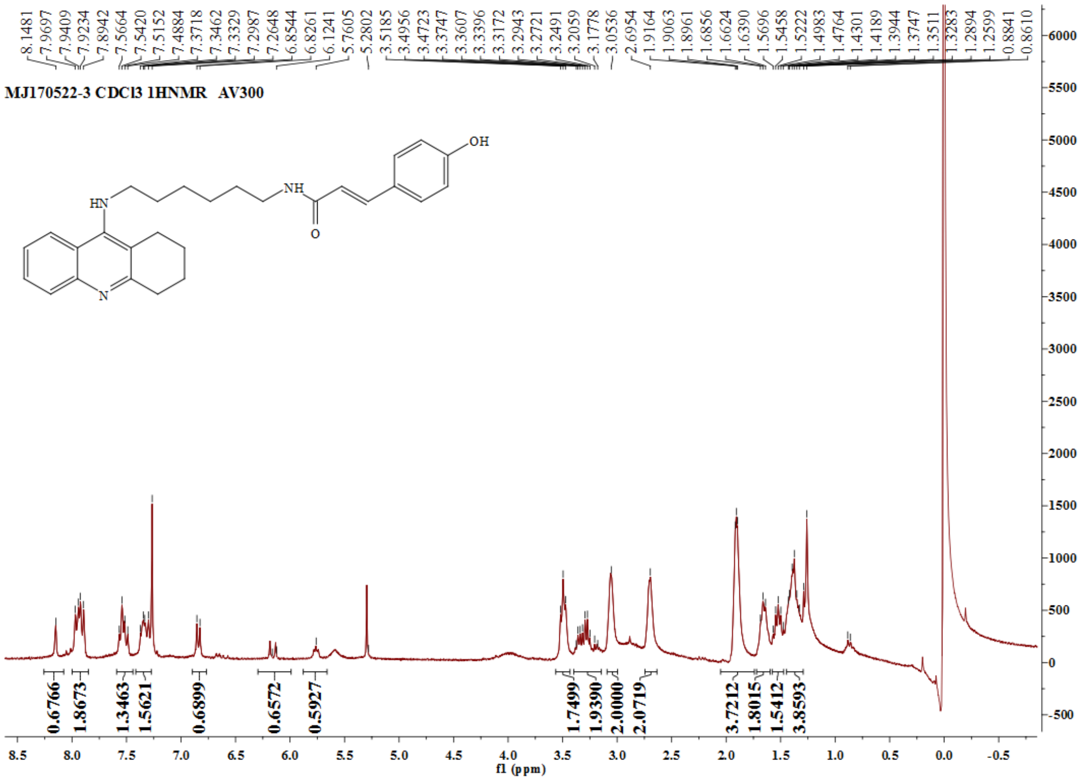

34 ^{13}C-NMR spectrum

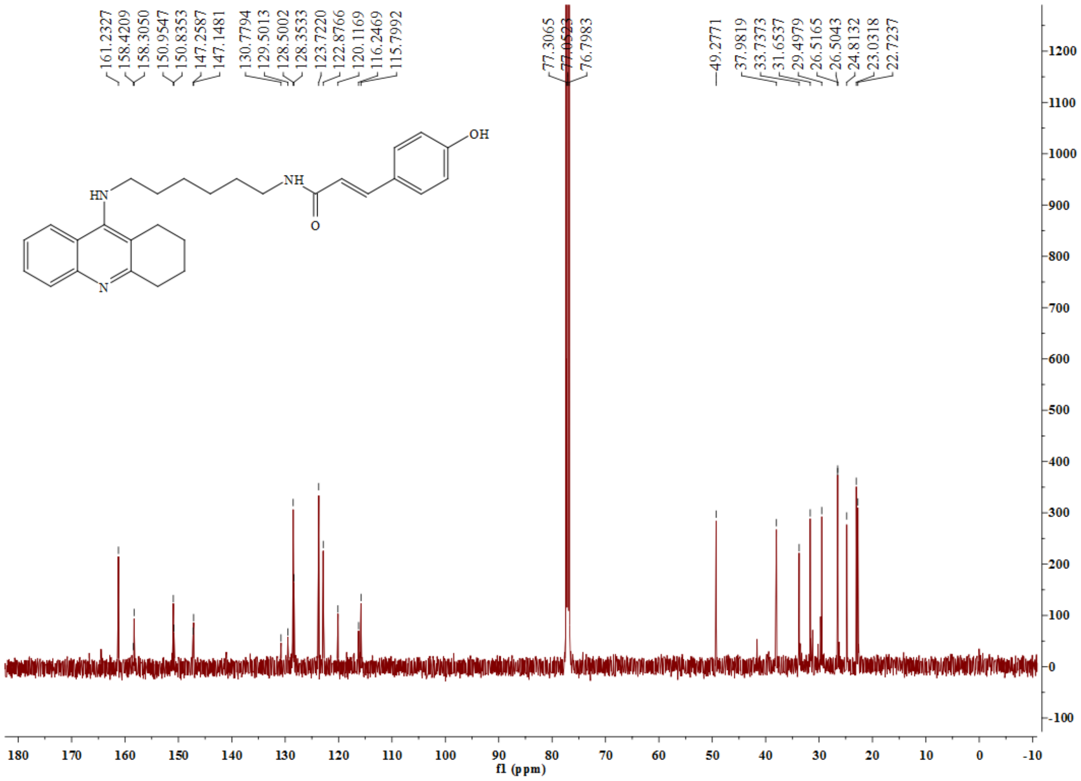

35 ^{1}H-NMR spectrum

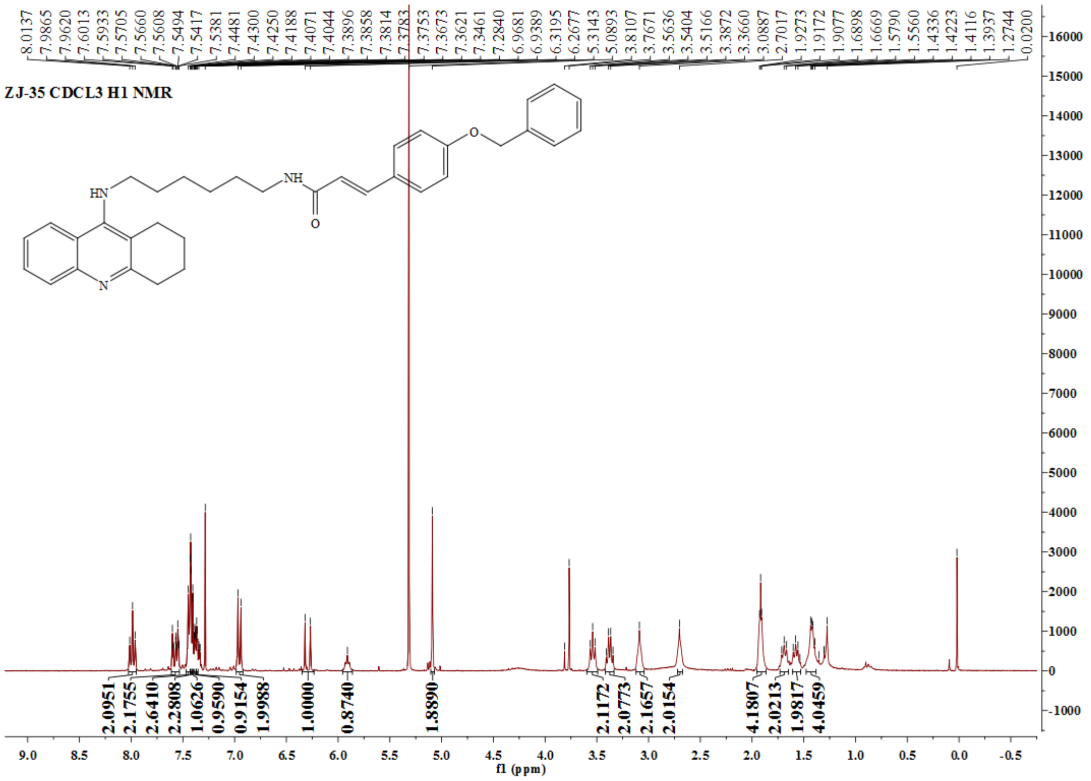

35 ^{13}C-NMR spectrum

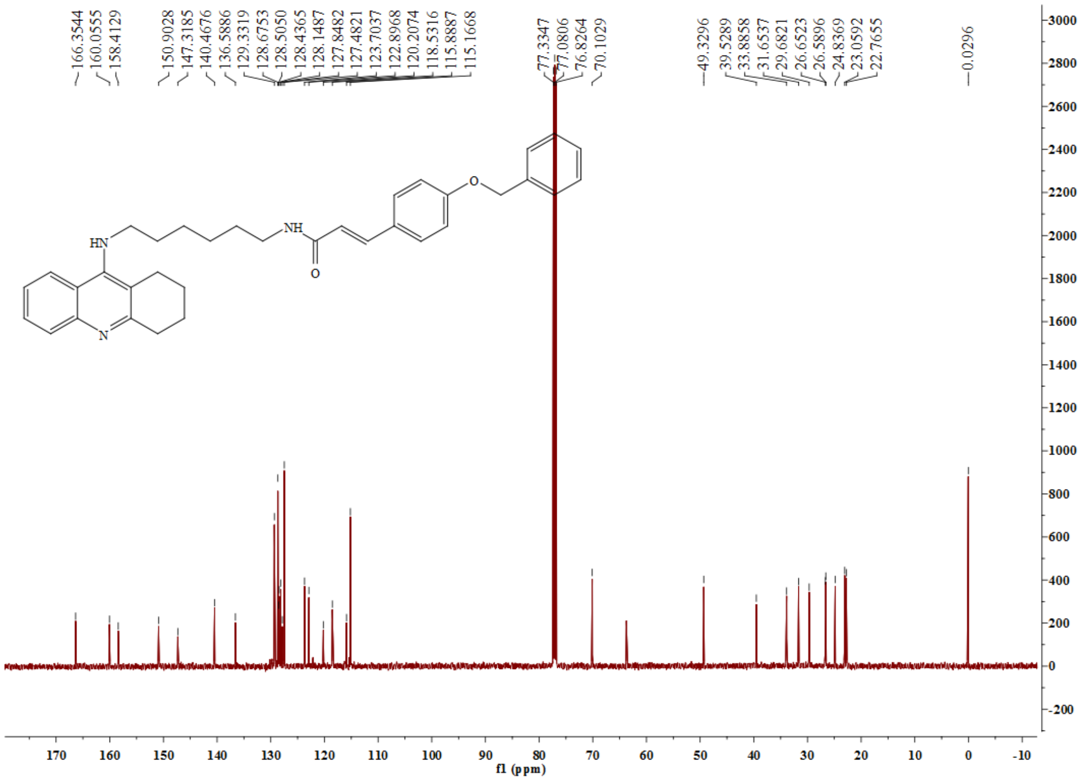

36 ^{1}H-NMR spectrum

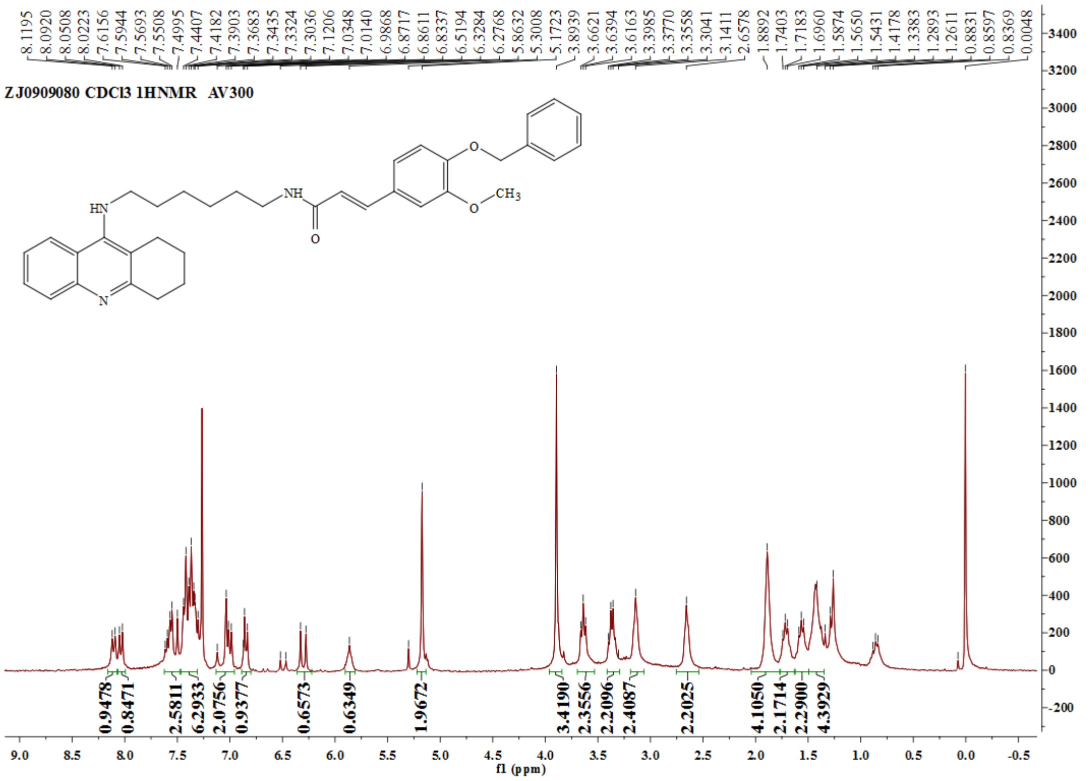

36 ^{13}C-NMR spectrum

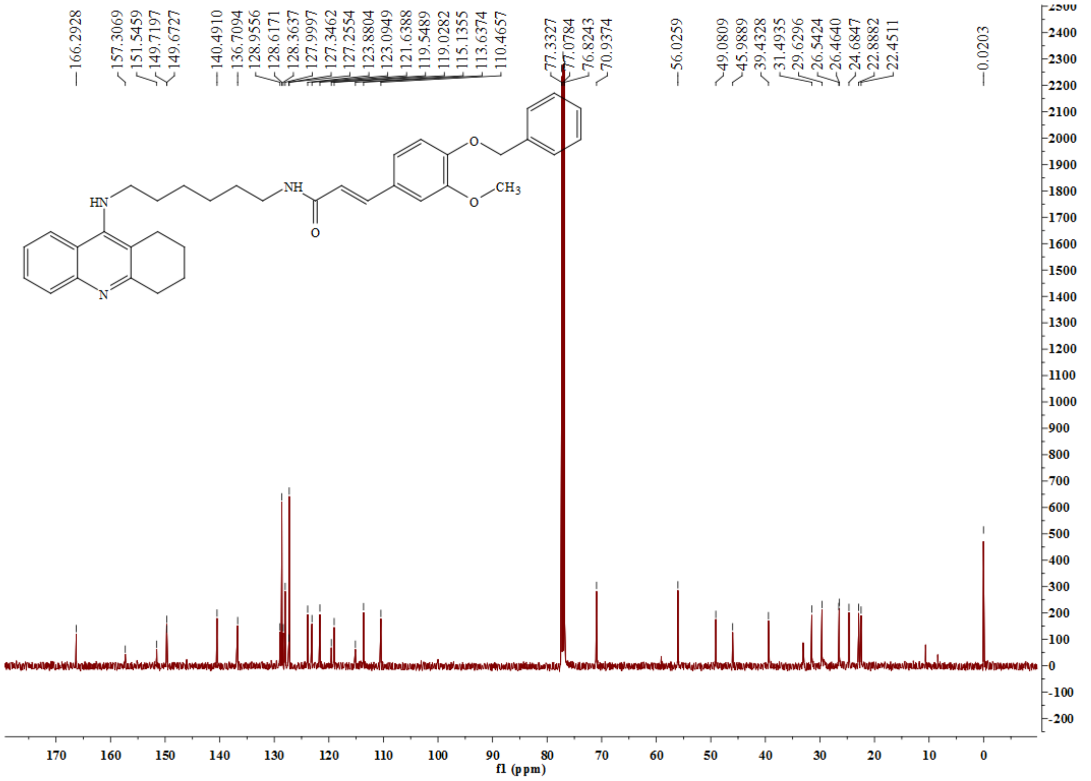

37 ^{1}H-NMR spectrum

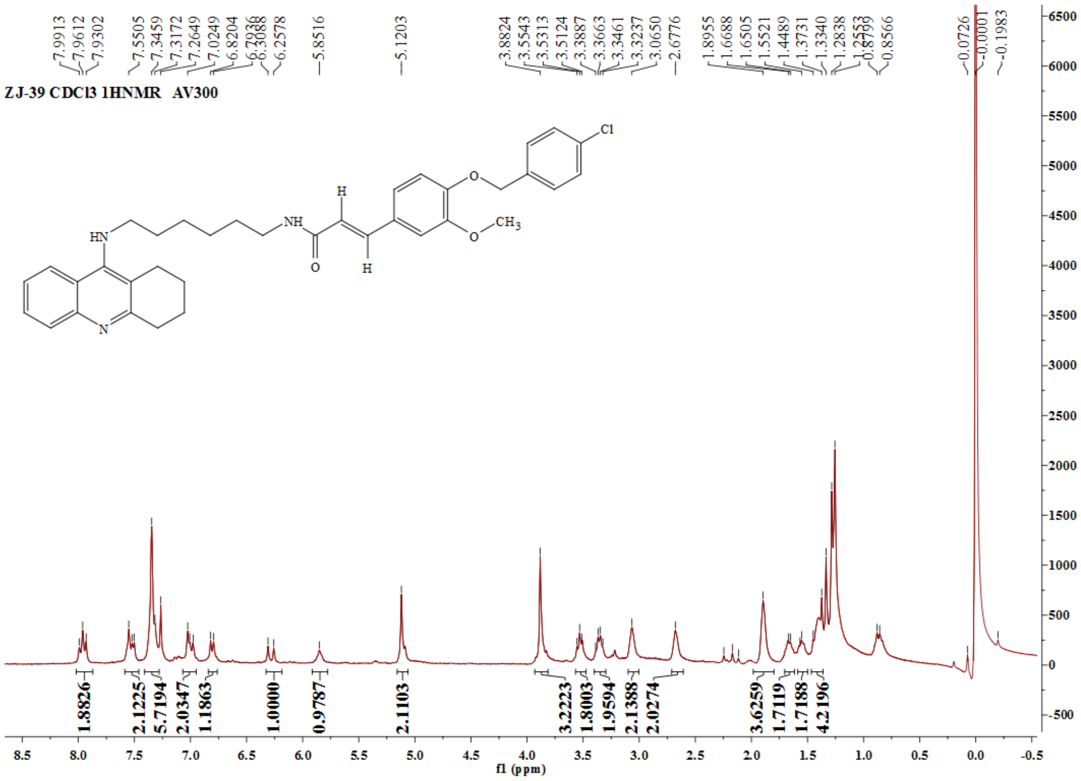

37 ^{13}C-NMR spectrum

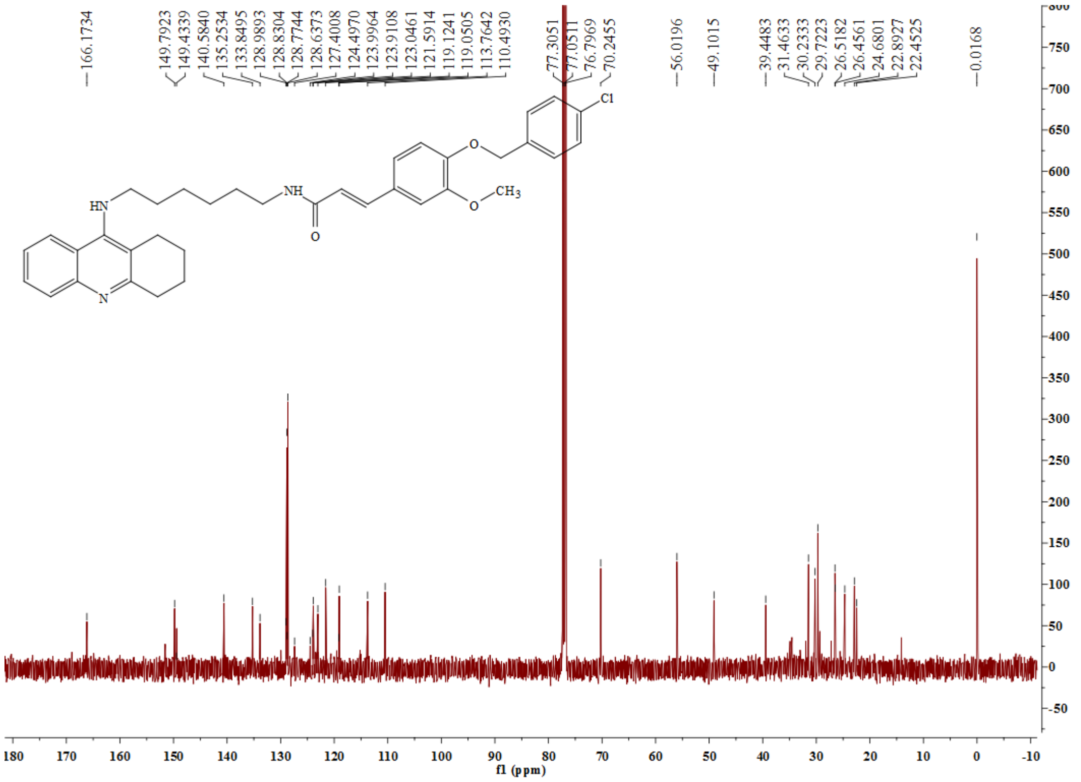

38 ^{1}H-NMR spectrum

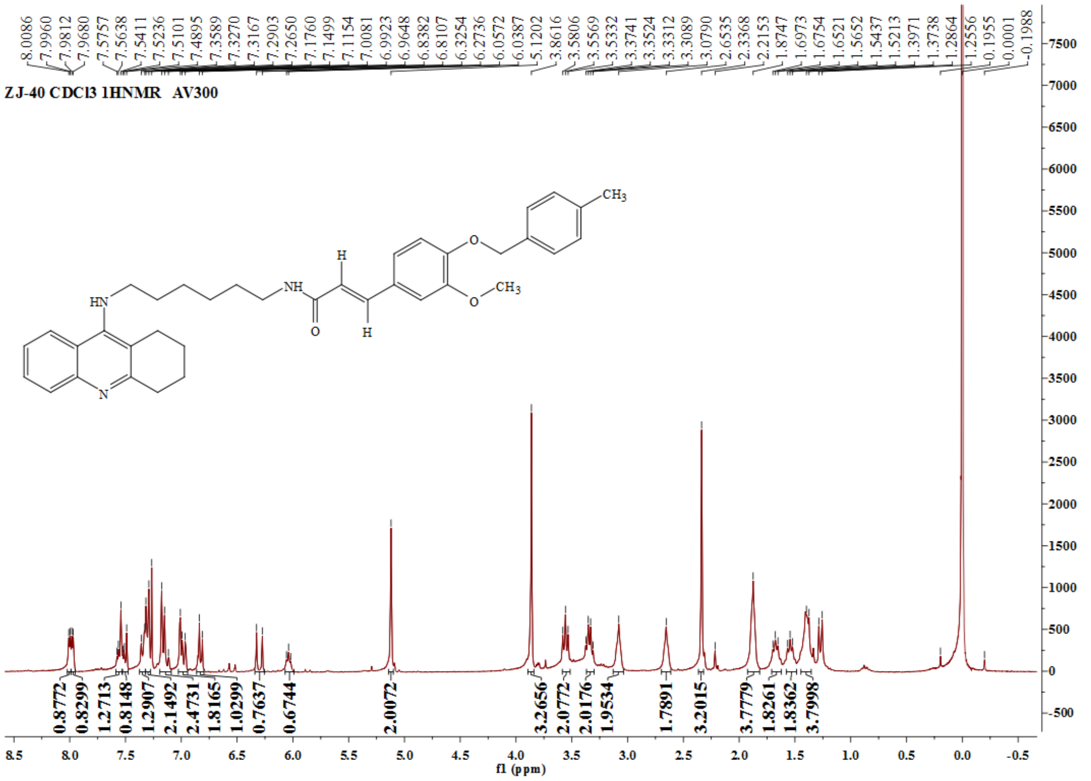

38 ^{13}C-NMR spectrum

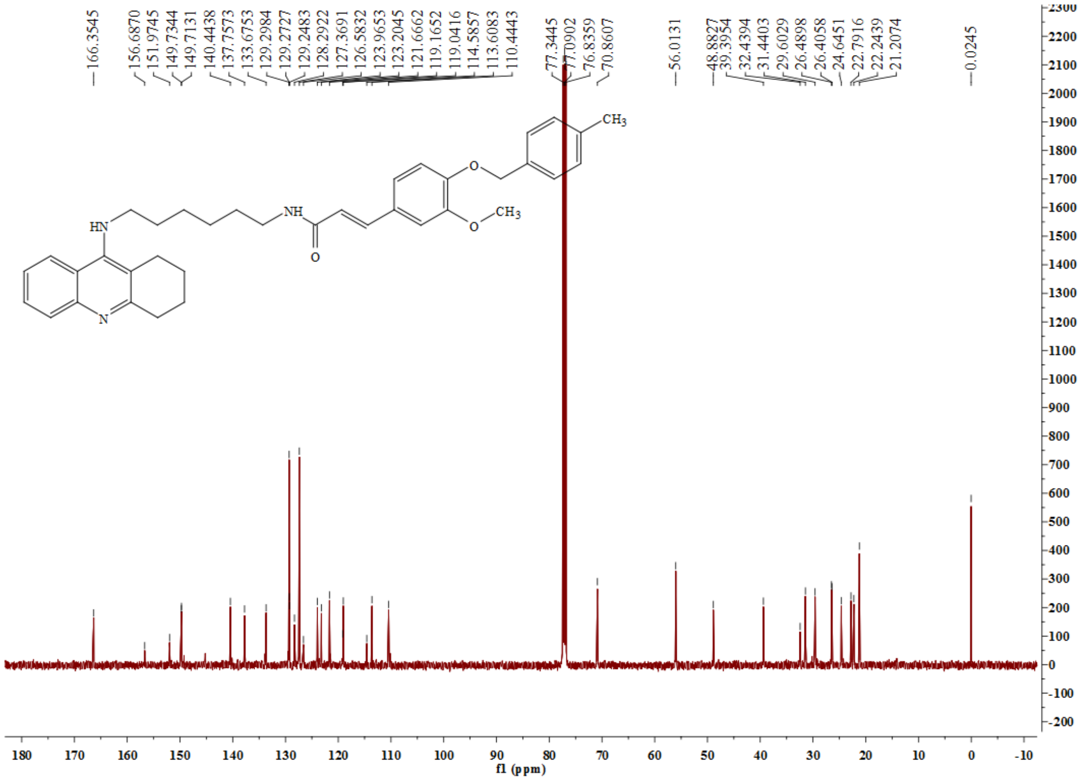

Table S1. Effects of oral administration of tacrine, **19**, **27** and **30** on scopolamine-induced cognitive impairment in ICR mice in the five days for learning and training.

Day	Latency to target (second)					
	control	model	tacrine	**19**	**27**	**30**
5	35.3 ± 6.8	96.9 ± 31.2	69.2 ± 11.0	54.1 ± 8.9	57.1 ± 6.8	59.1 ± 7.0
6	26.4 ± 4.5	80.6 ± 18.3	58.3 ± 7.9	36.5 ± 4.2	41.5 ± 5.0	47.3 ± 6.6
7	25.6 ± 3.7	81.4 ± 14.8	50.3 ± 6.3	36.6 ± 4.3	39.2 ± 4.3	46.4 ± 6.8
8	14.2 ± 2.1	62.6 ± 11.2	37.1 ± 5.1	35.2 ± 4.7	27.6 ± 2.5	32.4 ± 5.3
9	12.0 ± 1.6	47.3 ± 5.5	38.2 ± 2.6	26.1 ± 1.4	28.3 ± 1.7	33.2 ± 4.0
